# Synthesis and structure-activity relationships of novel 5-(hydroxamic acid)methyl oxazolidinone derivatives as 5-lipoxygenase inhibitors

**DOI:** 10.1080/14756366.2020.1786082

**Published:** 2020-07-08

**Authors:** Oludotun A. Phillips, Mira A. Bosso, Charles I. Ezeamuzie

**Affiliations:** a Faculty of Pharmacy, Department of Pharmaceutical Chemistry, Kuwait University, Safat, Kuwait; b Faculty of Medicine, Department of Pharmacology & Toxicology, Kuwait University, Safat, Kuwait

**Keywords:** Hydroxamic acid derivatives, oxazolidinone-hydroxamates, 5-lipoxygenase inhibitors, leukotrienes

## Abstract

Oxazolidinone hydroxamic acid derivatives were synthesised and evaluated for inhibitory activity against leukotriene (LT) biosynthesis in three *in vitro* cell-based test systems and on direct inhibition of recombinant human 5-lipoxygenase (5-LO). Thirteen of the 19 compounds synthesised were considered active ((50% inhibitory concentration (IC_50_) ≤ 10 µM in two or more test systems)). Increasing alkyl chain length on the hydroxamic acid moiety enhanced activity and morpholinyl-containing derivatives were more active than *N*-acetyl-piperizinyl derivatives. The IC_50_ values in cell-based assay systems were comparable to those obtained by direct inhibition of 5-LO activity, confirming that the compounds are direct inhibitors of 5-LO. Particularly, compounds **PH-249** and **PH-251** had outstanding potencies (IC_50_ < 1 µM), comparable to that of the prototype 5-LO inhibitor, zileuton. Pronounced *in vivo* activity was demonstrated in zymosan-induced peritonitis in mice. These novel oxazolidinone hydroxamic acid derivatives are, therefore, potent 5-LO inhibitors with potential application as anti-allergic and anti-inflammatory agents.

## 1. Introduction

The biosynthesis of bioactive leukotrienes (LTs) from arachidonic acid (AA) is catalysed by the crucial enzyme 5-Lipoxygenase (5-LO) with the help of 5-LO-activating protein (FLAP), the accessory protein that presents AA to 5-LO^1–4^. LTs are pro-inflammatory mediators that are implicated in a variety of human inflammatory and allergic diseases, including, asthma, allergic rhinitis, cardiovascular diseases (e.g. atherosclerosis and myocardial infarction), arthritis, inflammatory bowel diseases and certain forms of cancer^3^^,^^5–8^. Since 5-LO and its isoforms have been implicated in the pathophysiology and progression of several human diseases, it is therefore identified as a viable therapeutic target. Therefore, discovery and development of selective inhibitors of 5-LO for therapeutic intervention have been subjects of active research, which are presented in patents and scientific publications^3^^,^^7^^,^^9^^,^^10^.

Generally, 5-LO inhibitors can be classified into 4 main types based on their mechanism of action: (i) substrate or competitive analogues, (ii) inhibitors of 5-LO activating protein (FLAP), (iii) redox-active inhibitors that could interfere with the free radical chemistry, (iv) iron-chelating (Fe^3+^ ion) inhibitors – bind the putative iron at the active site^11^. Several compounds based on these four different types have been synthesised, isolated as natural products and investigated as potent selective inhibitors of 5-LO with promising therapeutic usefulness^3^^,^^7^. However, only few 5-LO inhibitors progress to clinical trials due to insufficient bioavailability, pharmacokinetics and/or toxicity related problems^12^.

**Figure 1.**
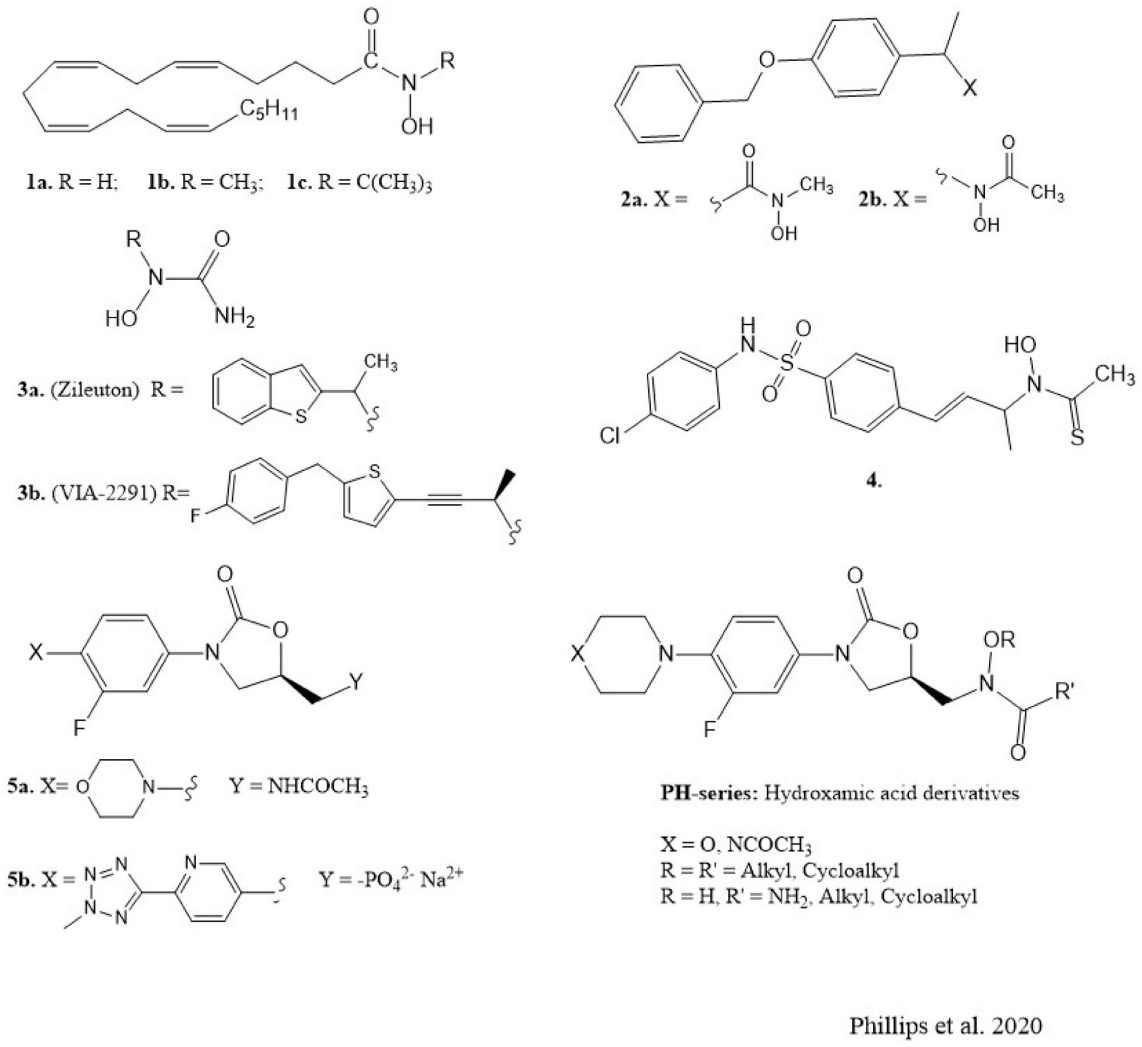
Chemical structures of 5-lipoxygenase inhibitors and antibacterial agents.

The scientific literature contains several reports on the discovery and synthesis of iron-complexing hydroxamates and hydroxyureas as 5-LO inhibitors with therapeutic potentials. The *N*-hydroxy-arachidonamides (**1a–c**, Figure 1) and the arylhydroxamic acids (**2a**, Figure 1) containing a terminal *N*-hydroxyl group (designated as “type A hydroxamic acids”) were the most potent inhibitors of 5-LO *in vitro* in their series but suffered rapid metabolic hydrolysis *in vivo*^13^^,^^14^. However, further studies identified the arylhydroxamic acids of structure (**2b**, Figure 1) having terminal small *N*-acyl groups like acetyl, which are designated as “type B hydroxamic acids” containing reversed substitution pattern. These “type B hydroxamic acids” are potent 5-LO inhibitors that are orally active and less prone to rapid *in vivo* metabolic hydrolysis. In addition, they showed higher plasma bioavailability, longer duration, and are more potent orally active inhibitors of leukotriene biosynthesis compared with the type A hydroxamic acids^15^. Further intensive investigations led to the discovery of Zileuton, (±)1–(1-(benzo[b] thiophen-2-yl)-ethyl)-1-hydroxyurea (**3a**, Figure 1), a hydroxyurea derivative of the Fe^3+^-chelating type inhibitor as the only 5-LO inhibitor currently in clinical use. Zileuton is commercially available as a racemate (*R* and *S* enantiomers), with both enantiomers exhibiting *in vitro* 5-LO inhibitory activity. However, it is plagued with significant drawbacks such as liver toxicity, weak potency and short half-life^7^, thus requiring higher frequency of administration (an extended-release dosage form has been introduced) accompanied by liver enzyme test. Based on these disadvantages, the need for intensive research to discover newer and potentially more effective 5-LO inhibitors with favourable pharmacokinetic, pharmacodynamic and safety profiles for treating related human diseases is highly imperative. In this light, the *N*-hydroxy urea atreleuton (VA-2291, **3b**, Figure 1) is currently in clinical trials for the treatment of cardiovascular diseases and vascular inflammation^7^, while the orally active hydroxamate containing a sulphonamide linker (**4**, Figure 1) has been reported as a 5-LO inhibitor with potential use in 5-LO mediated cancers^3^.

The oxazolidinone scaffold is an important pharmacophore in some clinically used antibacterial, psychoactive and anticoagulant agents^16–20^. In addition, structural modifications around the oxazolidinone 5-membered ring have resulted in novel compounds with antiepileptic, anticancer and antithyroid activities^19^^,^^21–23^. Based on the clinical success of drug molecules containing oxazolidinone scaffold, namely the antibacterial drugs linezolid (**5a**, Figure 1) and tedizolid phosphate (**5b**, Figure 1) and the well-documented pharmacological properties of the hydroxamic acid derivatives we decided to investigate a series of novel oxazolidinones containing type B hydroxamic acid functionality as inhibitors of 5-LO.

We hereby report the synthesis and structure-activity relationships of novel oxazolidinone hydroxamic acid (**PH-series** intermediates and **PH-series**, Figure 1) derivatives with potent *in vitro* 5-LO inhibitory activity based on the structural modifications around the oxazolidinone heterocycle as a scaffold for drug discovery.

## 2. Results and discussion

### 2.1. Chemistry

**Scheme 1.**
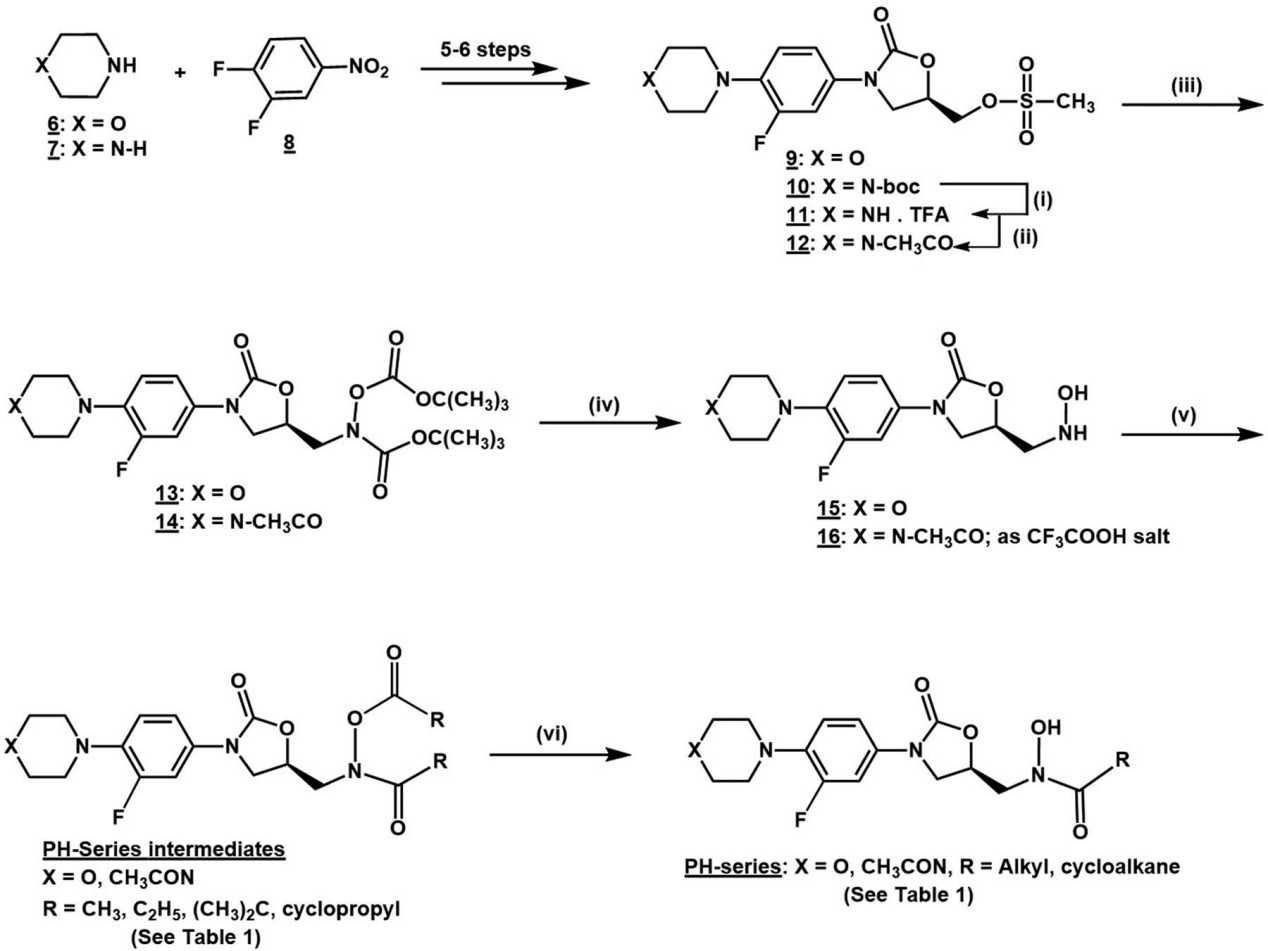
Synthetic route for the oxazolidinone hydroxamic acid derivatives. (i) DCM/TFA/0 °C r.t.; (ii) DCM/TEA/acetic anhydride/0 °C r.t.; (iii) DMF/NaH/tert-Butyl-N-(tertbutoxycarbonyl)carbamate/0 °C r.t. or 0–60 °C.; (iv) DCM/TFA/0 °C r.t.; (v) DCM/TEA/RCOCl or (RCO)_2_O/0 °C r.t.; (vi) MeOH/THF/NaOH/0 °C.

The oxazolidinone hydroxamic acid derivatives (**PH-series** intermediates and **PH-series**, Table 1) evaluated in this study were prepared as outlined in the chemical reaction sequence in Scheme 1 according to previously reported experimental methods with some modifications^24^^,^^25^. Starting from the commercially available starting materials morpholine **6**, piperazine **7** and 3,4-difluoronitrobenzene **8**, the key intermediate hydroxylamine derivatives **15** and **16** (TFA salt) were prepared in seven and ten chemical reaction steps, respectively. The nucleophilic acylation reactions of the hydroxylamine derivatives **15** and **16** (TFA salt) with different acyl anhydrides or acid chlorides yielded the respective 5-(*N*-alkanoxy-*N*-alkanamide) methyl oxazolidinone intermediate derivatives, **PH-series** intermediates. The final hydroxamic acid derivatives **PH-series** were obtained from base-promoted hydrolysis of the 5-*N*-alkanoxy-*N*-alkanamide oxazolidinones, **PH-series** intermediates. Ten of the compounds reported in this study were previously reported by our laboratory and were shown to be devoid of significant antibacterial or monoamine oxidases inhibitory activities^24^^,^^25^. Six of the previously reported compounds are morpholinyl derivatives (**PH-23**, **PH-199**, **PH-204**, **PH-205**, **PH-206**, and **PH-211**), and others are *N*-acetyl piperazinyl derivatives (**PH-201**, **PH-212**, **PH-208**, and **PH-213**). While the novel oxazolidinone hydroxamic acid derivatives, namely **PH-237**, **PH-239**, **PH-241**, **PH-244**, **PH-245**, **PH-246**, **PH-247**, **PH-249**, and **PH-251**) and their **PH-series** intermediates were prepared and reported for the first time in the present study. These novel compounds were fully characterised by appropriate spectroscopic and analytical methods as described in the experimental section. The characteristic signals for the *N*-hydroxamic acid N–OH appeared between 9.90 and 10.20 ppm, which is exchangeable with D_2_O and as broadband around 3171–3467 cm^−1^, in the nuclear magnetic resonance and infra-red spectra, respectively. The calculated log of the partition coefficient (Clog P), which is an indication of the lipophilicity of the compounds was estimated using the PerkinElmer ChemBioDraw Ultra 19.0 Computer Software and was reported as Clog P values in Table 1.

**Table 1.** Clog P values and *in vitro* inhibitory activity of oxazolidinone hydroxamates.

Compd. code	Structure of compounds	Clog P values	Human whole blood LTB_4_IC_50_ (CI)* μM	Human monocyte LTC_4_IC_50_ (CI)* μM	Mouse mast cell LTC_4_IC_50_ (CI)* μM	Direct inhibition of 5-LO activity. IC_50_ (CI)* μM
**PH-23**		0.7148	3.3 (1.2–9.2)	18.7 (8.9–39.4)	20.7 (13.7–31.2)	>30
**PH-199**		1.2438	1.4 (0.9–2.3)	8.2 (5.4–12.6)	6.3 (3.1–12.6)	11.8 (0.8–37.8)
**PH-204**		2.9859	10.7 (5.3–21.8)	>50	21.8 (10.9–43.5)	>30
**PH-205**		1.5528	2.3 (1.2–4.4)	14.1 (10.0–19.8)	13.8 (8.5–22.6)	3.9 (1.4–11.8)
**PH-206**		2.4779	7.9 (3.5–17.8)	>50	37.3 (16.8–82.9)	>30
**PH-211**		1.2988	1.3 (0.8–1.9)	7.5 (5.7–9.8)	8.7 (6.7–11.3)	3.5 (0.6–12.3)
**PH-237**		4.2239	>50	>50	>50	>30
**PH-239**		2.1718	3.5 (1.9–6.3)	8.0 (5.8–11.0)	3.1 (1.9–5.2)	4.7 (1.6–14.2)
**PH-241**		4.3018	2.2 (1.4–3.5)	3.2 (2.0–5.1)	2.8 (2.3–3.4)	3.3 (1.8–6.3)
**PH-245**		1.6278	3.4 (1.9–6.1)	ND	ND	ND
**PH-244**		2.1868	5.5 (3.5–8.5)	ND	ND	ND
**PH-246**		5.5419	1.5 (1.1–2.2)	7.1 (4.1–12.4)	3.3 (2.5–4.3)	21.6 (6.9–62.5)
**PH-247**		2.8308	2.3 (1.6–3.4)	2.5 (1.8–3.4)	2.9 (2.1–4.0)	3.4 (1.4–5.7)
**PH-249**		3.3598	0.7 (0.3–1.6)	0.9 (0.7–1.2)	1.3 (1.0–1.8)	1.9 (1.3–2.9)
**PH-251**		3.8888	1.9 (1.2–3.0)	2.4 (1.8–3.4)	0.2 (0.1–0.5)	1.6 (0.6–2.8)
**PH-201**		0.2758	38.0 (13.8–70.5)	>50	>50	>30
**PH-212**		1.1338	>50	32.7 (16.8–63.4)	>50	14.8 (3.2–38.5)
**PH-208**		2.0589	38.2 (7.5–84.5)	>50	>50	>30
**PH-213**		0.8739	22.5 (3.2–77.4)	23.3 (11.3–48.0)	>50	7.8 (1.0–38.7)
**Zileuton**		2.4830	0.7 (0.5–0.9)	0.5 (0.3–0.6)	0.4 (0.2–0.6)	0.8 (0.2–3.2)

*CI = 95% Confidence Interval; ND = not determined.

### 2.2. Pharmacology

Nineteen synthesised oxazolidinones containing the hydroxamic acid functionality were evaluated in three different cell-based *in vitro* assay systems for inhibition against the release of LTs, and by direct inhibition of 5-LO activity in a cell-free assay, using zileuton as a reference drug. The cell-based test systems included inhibition of 5-LO-dependent generation of LTB_4_ from activated human whole blood, inhibition of 5-LO product LTC_4_ from isolated human monocytes and inhibition of 5-LO product LTC_4_ from allergen/Immunoglobulin E (IgE)-activated bone marrow-derived mouse mast cells (BMMC). Furthermore, in order to confirm that the *in vitro* inhibitory activity of the compounds was not due to the direct toxic effects on the cells, the effect of the compounds on the viability of isolated human monocytes after 3 h and 24 h treatment was performed. Finally, *in vivo* anti-inflammatory studies were performed using the zymosan-induced peritoneal inflammation model in mice, which is a well-known model in which the LTs are known to play a critical role^26^.

The inhibitory activity data for the tested compounds obtained in three cell-based *in vitro* test systems - LTB_4_ release from human whole blood, LTC_4_ release from isolated human monocytes and LTC_4_ release from IgE/antigen-activated mouse mast cells, are shown in Table 1. Similar data on the direct inhibitory effect on the enzymatic activity of recombinant human 5-LO, together with the calculated Clog P (log of partition coefficient) values, which is an indication of the lipophilicity of each compound, are also shown in Table 1. In each case, the 50% inhibitory concentration (IC_50_) (95% confidence interval) values of the compounds were compared with those of the reference drug, and the only clinically available 5-LO inhibitor, zileuton.

Of the 19 compounds tested, 13 were found to have good (IC_50_ < 10 µM) to excellent (IC_50_ < 1 µM) inhibitory activity in at least two of the test systems, while six compounds (**PH-211**, **PH-239**, **PH-241**, **PH-247**, **PH-249**, and **PH-251**) were active in all four test systems. On human whole blood and isolated human monocytes, compound **PH-249** (IC_50_ = 0.7 µM and 0.9 µM, respectively), containing the heptanoyl moiety on the hydroxamate nitrogen, had potencies that were similar to those of zileuton (IC_50_ = 0.7 µM and 0.5 µM, respectively, Table 1), whereas on mast cells that were activated by an allergic mechanism, compound **PH-251,** containing the octanoyl moiety on the hydroxamate nitrogen, was the most active (IC_50_ = 0.2 µM). In this later test system, compound PH-251 was slightly more potent than zileuton (IC_50_ = 0.4 µM). In the cell-free system, both **PH-249** and **PH-251** were also the most active (IC_50_ = 1.9 µM and 1.6 µM, respectively, Table 1).

Structure-activity relationship revealed that the inhibitory activities of the compounds were highly dependent on the substitution pattern around the phenyloxazolidinone moiety, whereby the morpholinyl-containing derivatives were more active compared with the *N*-acetyl-piperizinyl-containing derivatives. Also, the N–OH hydroxamate-containing oxazolidinones were significantly more active than their respective precursors, with the exception of the *N*-(hexanoyloxy)hexanamide derivative **PH-246**, which was also quite active in cell-based systems (IC_50_ values of 1.5 µM, 7.1 µM and 3.3 µM, for whole blood, isolated monocytes and mast cells, respectively, Table 1). The reason for this discrepancy is not immediately clear given that the compound contains an *N*-hexanoyloxy functionality which might make it liable to hydrolysis by plasma esterases. Moreover, it is possible that the observed activity in these systems may reflect that of its hydrolytic metabolite, **PH-247**. However, since **PH-246** is the only 5-*N*-alkanyloxy-*N*-alkanamide derivative with reasonable activity compared to that of **PH-247**, this may suggest that the *N*-hexanoyloxy moiety is more readily hydrolysed by hydrolases than the other *N*-alkanoyloxy groups. Hence, **PH-246** may be serve as a pro-drug for the release of **PH-247** *in vivo*. In addition, the absence of cellular esterase activity may explain why **PH-246** is very weak in inhibiting the activity of isolated 5-LO enzyme in cell-free experiments. Further studies are planned to investigate the stability of these compounds *in vitro* at different pH conditions and in the presence of hydrolases in plasma.

The finding that most of the active compounds were generally more active in human whole blood than on isolated human monocytes is very interesting, given that in drug discovery studies, compound instability in plasma is a well-recognised problem. One possible explanation could be that the compounds are more active on granulocytes than monocytes or that a plasma factor enhances their entry into cells. These possibilities are currently being investigated.

It was further observed that all the *N*-acetyl-piperizinyl containing oxazolidinone derivatives tested showed either weak activity (IC_50_ values in the ranges of 10.0–30 µM) or were essentially inactive (IC_50_ values > 30 µM, Table 1). This demonstrates that the presence of the morpholine heterocycle is essential for activity in these compounds.

Further analysis also revealed that among the hydroxamates, activity generally increased with the length of the straight-chain hydrocarbon moiety on the hydroxamate nitrogen, until the heptanoyl group. The increase in chain length to the octanoyl group resulted in a slight decrease in potency. On the other hand, cyclic hydrocarbon-containing analogues (cyclobutyl and cyclopentyl), were generally less potent, with the exception of the cyclopropyl derivative, **PH-211**. In the morpholine containing hydroxamic acids, IC_50_ values seem to correlate with the Clog P values (indicative of the lipophilicity of the compounds), thus suggesting that the optimal Clog P values are in the range of 3.3598 for **PH-249** and 3.8888 for **PH-251**, (Table 1).

Data from studies with an isolated 5-LO enzyme (Table 1) showed that the inhibitory effect of the compounds paralleled their effects in cell-based assays. For example, compounds **PH-249** and **PH-251**, which were the most active in inhibiting 5-LO activity in the cell-free system were also the most active in inhibiting LT biosynthesis in cell-based systems, whereas the inactive analogue, **PH-237** (containing the isovaleryloxy and isovaleryl moieties on the hydroxamate nitrogen), was inactive in both systems. These results indicate that the mechanism of inhibition of LT biosynthesis by the active compounds is by direct inhibition of 5-LO, and that their potencies are comparable to that of zileuton. In addition, one of the most active compounds, **PH-251** demonstrated an outstanding potency in inhibiting LT biosynthesis in mouse mast cells (IC_50_ = 0.2 µM), which was slightly better than that of zileuton (IC_50_ = 0.4 µM, Table 1). Since mast cell activation through IgE/allergen interaction is the basis of all allergic diseases^27^, compounds like **PH-251** have a particularly high potential of being developed into useful drugs for the treatment of allergic diseases, including asthma.

From these results, it can be concluded that these novel oxazolidinones, especially the morpholinyl-containing derivatives, are potent inhibitors of 5-LO that affect cells from both humans and mice. Their action is also independent of the mode of cell activation, as they equally affect cells activated by lipopolysaccharide (LPS)/N-formyl methyl-leucyl-phenylalanine (FMLP), calcium ionophore and antigen-antibody immune complex. These findings are very important in that they show that the active compounds have the potential of being useful, not only in allergic diseases, such as asthma, allergic rhinitis, atopic dermatitis, but also in many other inflammatory diseases in which LTs, induced by a variety of stimuli, are known to be involved^6^^,^^7^.

**Figure 2.**
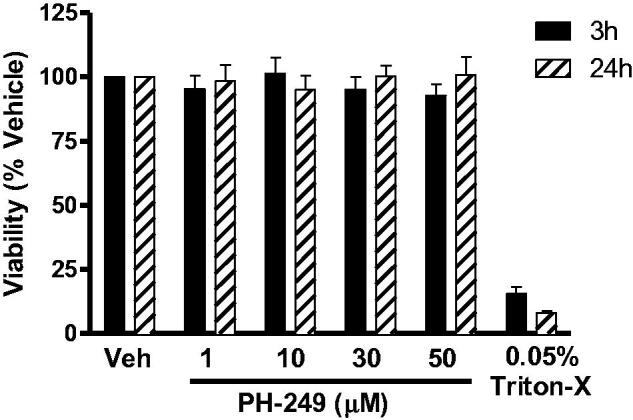
Effect of compound **PH-249** on the viability of isolated human monocytes. Cells were exposed to the test compound or triton-X, as positive control, for 3 h or 24 h, and viability was assessed by the MTT method, *n* = 3.

In order to exclude the possibility that the *in vitro* inhibitory activity of the compounds, was a result of direct toxicity of the compound on the cells, we studied the effect of the most active compound (**PH-249**) on the viability of isolated human monocytes after 3 h and 24 h treatment. As shown in Figure 2, at concentrations up to 50 μM (a concentration far beyond that required for 100% inhibition of LT release), no significant effect on cell viability was detected, whether cells were cultured with the drug for 3 h or 24 h. This shows that the compounds are not cytotoxic.

**Figure 3.**
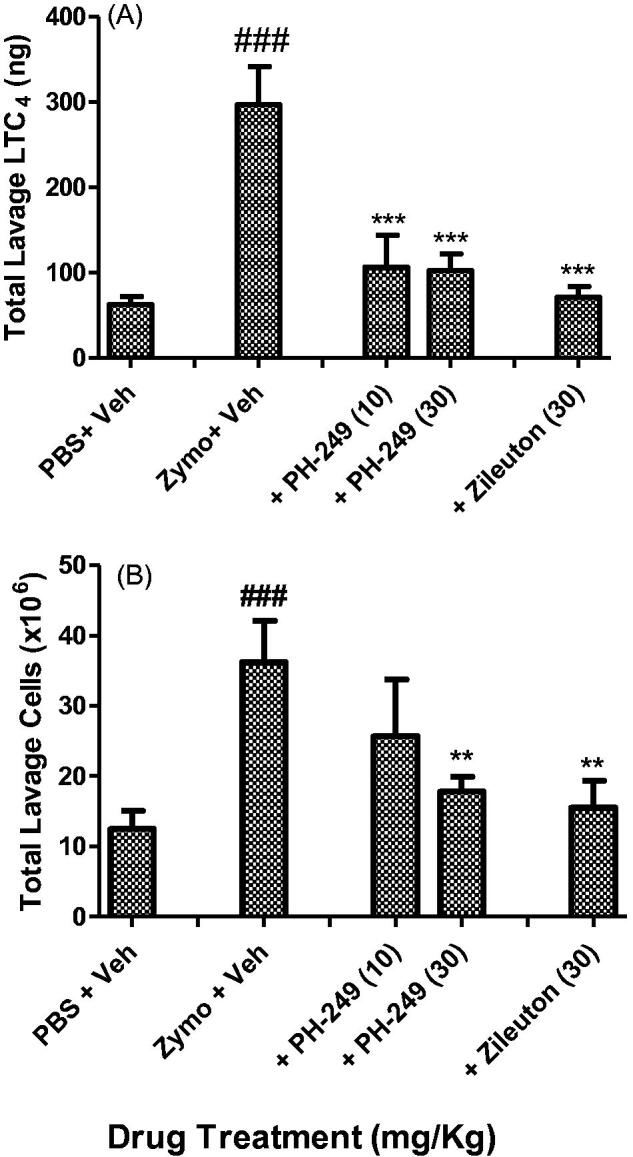
*In vivo* inhibitory effect of compound **PH-249** on zymosan-induced peritoneal inflammation in mice. Animals were pre-treated subcutaneously with PH-249, zileuton or vehicle, 30 min before induction of peritoneal inflammation with intraperitoneal injection of 0.2 ml activated zymosan (2 mg/ml). Peritoneal lavage fluids obtained after 2 h were analysed for total LTC_4_ content (A) and total cellular content (B). Values are means ± sem, *n* = 6–7. ###*p* < 0.001 with respect to PBS plus vehicle; ***p < 0.001; **p < 0.01, with respect to zymosan plus vehicle.

To determine if the compounds were active *in vivo*, the representative compound **PH-249** was also tested in the zymosan-induced peritoneal inflammation, a well-known model in which LT is known to play a critical role^26^. As shown in Figure 3, intra-peritoneal administration of zymosan (plus vehicle) resulted in a highly significant increase in the cellular influx and LTC_4_ content of the recovered peritoneal lavage fluid after 3 h. Pre-treatment with 10 or 30 mg/kg of **PH-249** resulted in highly significant inhibition of total LTC_4_ content of the lavage fluid (*p* < 0.001), Figure 3(A), but for cellular infiltration, only the inhibition by the higher dose reached statistical significance, *p* < 0.01, (Figure 3(B)). At 30 mg/kg, zileuton achieved a similar effect with respect to both parameters. These results show that the representative compound **PH-249** containing the heptanoyl moiety has *in vivo* biological activity that is comparable to that of the reference drug, zileuton and thus suggests that these novel oxazolidinone hydroxamic acid derivatives have potential anti-inflammatory actions *in vivo*.

## 3. Conclusion

In conclusion, the synthesised novel oxazolidinone hydroxamic acid derivatives were screened in four *in vitro* test systems for inhibitory activity against the release of LTs or direct inhibition of 5-LO enzyme activity. Thirteen of the compounds had good or excellent activity in at least two test systems, while six compounds were active in all four systems. The most active compounds had activities that were comparable to those of zileuton. One compound, **PH-251**, was particularly effective on mast cell LT release induced by allergen/IgE interaction (IC_50_ = 0.2 µM). Structure-activity relationship studies revealed that oxazolidinone derivatives containing the (*N*-(OH)COR) hydroxamate functionality with relatively longer straight-chain alkyl groups (-R) along with the morpholine heterocycle demonstrated the best activity. The optimal alkyl chain length appeared to be C = 6 (**PH-249**, R = hexyl), as extending the length to C = 7 (**PH-251**, R = heptyl) resulted in either a slight loss or no change in activity, except for mast cells in which a further increase in activity was observed. Results also suggest that the active compounds are non-toxic and possess strong *in vivo* anti-inflammatory activity. Hence, they have the potential for development as drugs for the treatment of allergic and inflammatory diseases.

## 4. Experimental

### 4.1. Synthesis

#### 4.1.1. Materials and methods

The starting materials 3,4-difluoronitrobenzene, morpholine, piperazine, n-butyllithium, sodium hydride, and other common reagents and solvents used for the synthesis of the oxazolidinones, including, dichloromethane (DCM), diethyl ether, dimethylformamide (DMF), ethyl acetate, methanol, tetrahydrofuran (THF) were purchased from Merck (formerly Sigma-Aldrich) Germany. Among the previously reported compounds, six are morpholinyl derivatives (**PH-23**, **PH-199**, **PH-204**, **PH-205**, **PH-206**, and **PH-211**), and the others are *N*-acetyl piperazinyl derivatives (**PH-201**, **PH-212**, **PH-208** and **PH-213**). These were previously reported from our laboratory and synthesised according to literature methods^24^^,^^25^. The remaining nine oxazolidinone hydroxamic acid derivatives, namely **PH-237**, **PH-239**, **PH-241**, **PH-244**, **PH-245**, **PH-246**, **PH-247**, **PH-249** and **PH-251** and their respective **PH-series** intermediates are reported for the first time (Table 1). Purification of compounds was performed on silica gel column chromatography using silica gel (Kieselgel 60, 70–230 mesh; Aldrich-Sigma, Germany) and thin-layer chromatography (TLC) was conducted on 0.25 mm pre-coated silica gel plates (60F_254_, Merck, Darmstadt, Germany). Solid products were recrystallised from a suitable solvent and or solvent mixtures. Melting points were determined on a Stuart Scientific melting point apparatus (SMP1, Stuart, Stone, UK) and were uncorrected. Further structural elucidation was performed using ^1^H- and ^13 ^C-NMR (decoupled experiments) spectra in DMSO-d_6_ using solvent peaks as reference signals and were recorded on Bruker DPX 400 MHz and Bruker Avance II 600 NMR spectrometers. Two-dimensional NMR spectra experiments, namely 2 D COSY (COrrelated SpectroscopY) and 2 D HSQC (Heteronuclear Signal Quantum Coherence) experiments were also performed on representative compounds (**PH-242** (containing the cyclobutylcarbonyloxy and the cyclobutylcarbonyl moieties on the hydroxamate nitrogen), **PH-245** (containing the cyclobutylcarbonyl group on the hydroxamate nitrogen), **PH-246** (containing hexanoyloxy and the hexanoyl moieties on the hydroxamate nitrogen), **PH-247** (containing the hexanoyl moiety on the hydroxamate nitrogen) to assist in proton and carbon assignments. Chemical shifts and coupling constants (J, Hz) of protons and carbons are reported in parts per million (ppm) downfield and up-field from solvent DMSO-d_6_ (*δ* = 2.5; 39.7) peak as reference. Mass spectra data were recorded on a Thermo Scientific DFS Gas Chromatography/Mass Spectrometer (DFS GC-MS, Thermo Fisher Scientific, Bremen, Germany) and Waters QToF/Mass Spectrometer (LC-MS/MS ESI, Waters Corporation, Milford, MA). Infra-red (IR) spectra were recorded on JASCO FT-IR-6300 (JASCO, Tokyo, Japan) Spectrometer. Elemental analyses were performed on an Elementar Vario Micro Cube CHN Analyser apparatus (Elementar, Langenselbold, Germany), and analyses indicated by the symbols of the elements (CNH) were within ±0.4% of the theoretical values. Elemental analyses (CHN) were used to confirm the purity of all newly synthesised compounds (>95%), Analyses were performed by the General Facility-Science (GF-S), Faculty of Science, Kuwait University, Kuwait. The structures of the oxazolidinones and their Clog P values were sketched and estimated, respectively using the PerkinElmer ChemBioDraw Ultra 19.0 Software.

#### 4.1.2. (R)-tert-butyl (tert-butoxycarbonyl)oxy ((3-3-fluoro-4-morpholinophenyl)-2-oxooxazolidin-5-yl) methyl)carbamate (13)

An ice-cooled (0 °C) solution of tert-butyl *N*-(*tert*-butoxycarbonyloxy) carbamate (4.97 g, 21.31 mmol) in anhydrous DMF (30 ml) under nitrogen was treated portion-wise with 60% sodium hydride in mineral oil (770 mg, 22.75 mmol) and stirred for 30 min. The reaction mixture was treated with drop-wise addition of a solution of [*N*-3-(3-fluoro-4-morpholinylphenyl)-2-oxo-5-oxazolidinyl] methyl methanesulfonate (6.00 g, 16.02 mmol) in DMF (70 ml). The reaction mixture was stirred to room temperature for 60 h, quenched by addition of water (200 ml) and extracted with ethyl acetate (3 × 100 ml). The ethyl acetate (EtOAc) extract was diluted with hexane (60 ml) and washed with water, brine, dried (Na_2_SO_4_), filtered and concentrated to give the crude as a brownish oil. Silica gel column chromatography eluted with EtOAc-Hexane 2:1 gave the title compound **13**^24^^,^^25^ as a pale-yellow viscous oil (7.0 g, yield 85%), solidifies upon cooling in the fridge to a yellow solid after a long period of time. ^1^H-NMR (DMSO-d_6_, 600 MH_Z_): *δ* 7.51 (dd, 1H, *J* = 15.0 Hz, 2.5 Hz, phenyl H), 7.19 (dd, 1H, *J* = 8.8 Hz, 2.5 Hz, phenyl H), 7.09 (t, 1H *J* = 9.6 Hz, phenyl H), 4.87 (m, 1H, oxazolidinone H), 4.15 (t, 1H, *J* = 4.6 Hz, oxazolidinone H), 3.97 (m, 1H, oxazolidinone H), 3.82 (m, 1H, methylene H), 3.72–3.76 (br. t, 5H, morpholine H and methylene H), 2.97 (br. t, 4H, morpholine H), 1.41–1.48 (br,18H). MS 511.2 (M^+^)^24^^,^^25^

#### 4.1.3. (R)-3-(3-fluoro-4-morpholinophenyl)-5-((hydroxyamino)methyl)oxazolidin-2-one (15)

Compound **13** (14.69 g, 28.71 mmol) was dissolved in anhydrous DCM (25 ml) and cooled to 0 °C in an ice-bath. This solution was treated with rapid dropwise addition of trifluoroacetic acid (20 ml) and the reaction mixture was stirred overnight. The reaction mixture was concentrated to give a gummy residue, which was treated with a 10% potassium carbonate solution in water (100 ml) to give a basic solution. The resulting gelatinous precipitate was collected by filtration to give an off-white solid (8.40 g), the filtrate was extracted with DCM and the DCM layer was dried (Na_2_SO_4_) and concentrated to give a second crop (0.240 g). The titled compound **15** was obtained as an off-white solid (8.64 g, yield 94%)^24^^,^^25^, mp 134–137 °C. This product was used for further reactions without further purification. ^1^H-NMR (DMSO-d_6_, 400 MH_Z_): *δ* 7.50 (dd, 1H, *J* = 15.1 Hz, 2.5 Hz, phenyl H), 7.45 (s, N–OH, 1H), 7.20 (dd, 1H, *J* = 2.1 Hz, 8.7 Hz, phenyl H), 7.06 (t, 1H, *J* = 9.6 Hz, phenyl H), 6.02 (br. s, 1H, N–H), 4.70–4.83 (m, 1H, oxazolidinone H), 4.09 (t, 1H, *J* = 8.9 Hz, oxazolidinone H), 3.82 (dd, 1H, *J* = 6.8 Hz, 8.9 Hz, oxazolidinone H), 3.73 (br. t, 4H, *J* = 4.6 Hz, morpholine H), 2.97–3.10 (br. m, 6H, CH_2_N(OH)H, partially overlaps with the morpholine H triplet signal at 2.96, *J* = 4.6 Hz), ^13 ^C-NMR (DMSO-d_6_, 600 MH_Z_): *δ* 165.28, 155.35, 154.21, 153.74, 135.42, 135.37, 133.64, 133.57, 119.19, 119.17, 114.04, 114.02, 106.69, 106.52, 70.66, 70.04, 66.11, 56.31, 50.68, 48.12. MS 311.1 (M^+^)^24^^,^^25^.

#### 4.1.4. General procedure for the synthesis of the (R)-N-((3-(3-fluoro-4-morpholinophenyl)-2-oxoxazolidin-5-yl)methyl)-N-hydroxyalkanamide (PH-series)

A solution of the *N*-hydroxylamine derivative, compound **15** (1.0 eq.) in anhydrous DCM or CH_3_CN (30–50 ml) was treated with triethylamine (6.0 eq.) and dropwise addition of the respective acid anhydride or acid chloride (3.0 eq.) at 0 °C and stirred to room temperature overnight. The reaction mixture was diluted with a 10% solution of potassium carbonate (20 ml) and the DCM layer was separated and washed with water, brine, dried (Na_2_SO_4_), filtered and concentrated to give the crude product. Purification by normal phase silica gel column chromatography and/or recrystallised using suitable organic solvent or mixtures to give the intermediate products *N*-alkanoxy-*N*-((3-3-fluoro-4-morpholinophenyl)-2-oxooxazolidin-5-yl) methyl)alkanamide **PH-series** intermediates (**PH-237**, **PH-240**, **PH-242**, **PH-243**, **PH-246**, **PH-248** and **PH-250**). A solution of the **PH-series** intermediates (1 eq.) in methanol:THF (4:1, v/v) was stirred at 0 °C and treated with a 1.0 eq. NaOH solution in water (∼20 ml). The reaction mixture was stirred for 1 h 10 min and neutralised to pH of ∼7 by addition of a solution of 1.0 eq. HCl in water (30 ml). The reaction mixture was concentrated to remove THF and methanol, and the aqueous residue was saturated with NaCl and extracted with DCM (2 × 30 ml) and the organic layer was separated, dried (Na_2_SO_4_), filtered and concentrated to give the crude product. Purification was performed either by silica gel column chromatography and/or recrystallisation using suitable organic solvent or mixtures to give the (*R*)-*N*-((3-(3-fluoro-4-morpholinophenyl)-2-oxoxazolidin-5-yl)methyl)-*N*-hydroxy-alkanamide derivatives (**PH-239**, **PH-241**, **PH-244**, **PH-245**, **PH-247**, **PH-249**, and **PH-251**) as the final products.

##### 4.1.4.1. (R)-N-((3-(3-fluoro-4-morpholinophenyl)-2-oxooxazolidin-5-yl)methyl)-N-hydroxy-3-methylbutanamide (PH-239)

The intermediate compound **PH-237**, was prepared via the general procedure from compound **15** (1.50 g, 4.82 mmol), isovaleric anhydride (2.83 ml, 14.46 mmol), triethylamine (4.05 ml; 28.41 mmol) in anhydrous DCM (30 ml) to give crude product. Purification by silica gel column chromatography (EtOAc-Hexane, 1:2 to 1:1) gave the intermediate (*R*)-*N*-((3-(3-fluoro-4-morpholinophenyl)-2-oxooxazolidin-5-yl)methyl)-3-methyl-*N*-((3-methylbutanoyl)oxy)butanamide **PH-237**, as a white solid 0.815 g, yield 35%, mp 89–90 °C. IR (KBr, cm^−1^): *ν* 2960, 2871, 2822, 1797, 1759, 1672, 1520, 1471, 1445, 1404, 1329, 1289, 1169, 1136, 1119, 1065. ^1^H-NMR (CDCl_3_, 600 MH_Z_): *δ* 7.46 (dd, 1H, *J* = 2.6 Hz, 14.3 Hz, phenyl H), 7.11 (m, 1H, phenyl H), 6.94 (t, 1H, *J* = 9.1 Hz, phenyl H), 4.80–4.84 (br. m, 1H, oxazolidinone H), 4.10 (d, 2H, *J* = 4.6 Hz, methylene CH_2_), 4.05 (t, 1H, *J* = 8.9 Hz, oxazolidinone H), 3.88–3.91 (m, 5H, morpholine H and oxazolidinone H), 3.07 (t, 4H, *J* = 4.7 Hz, morpholine H), 2.39 (d, 2H, *J* = 7.1 Hz, NOCOCH_2_CH(CH_3_)_2_), 2.19–2.51 (m, 1H, NOCOCH_2_CH(CH_3_)_2_), 2.06–2.12 (m, 3H, NCOCH_2_CH (CH_3_)_2_ and NCOCH_2_CH (CH_3_)_2_), 1.05 (dd, 6H, *J* = 1.4 Hz, 6.6 Hz, NOCOCH_2_CH(CH_3_)_2_), 0.93 (dd, 6H, *J* = 6.1 Hz, 13.0 Hz, NCOCH_2_CH(CH_3_)_2_). ^13 ^C-NMR (DMSO-d_6_, 600 MH_Z_): *δ* 155.33 (d, *J* = 243.20 Hz), 153.70, 135.55 (d, *J* = 8.76 Hz), 133.32 (d, *J* = 10.80 Hz), 119.22 (d, *J* = 4.16 Hz), 114.13, (d, *J* = 2.80 Hz), 106.67 (d, *J* = 25.88 Hz), 70.10, 66.10, 50.66, 50.30, 47.15, 24.95, 22.11. MS 479.4 (M^+^). Anal. Calcd. for C_24_H_34_FN_3_O_6_: C: 60.11, H: 7.15, N: 8.76; found C: 59.71, H: 7.41, N: 8.55. A solution of the intermediate **PH-237** (0.80 g, 1.67 mmol) in MeOH:THF (28:7 ml) was treated with NaOH solution (133 mg in 20 ml water). Purification by recrystallisation (EtOAc-hexane 2:1) gave the titled compound **PH-239** as a white solid (328 mg, yield, 55%), mp 122–124.5 °C. IR (KBr, cm^−1^): *ν* 3384, 3196, 2955, 2869, 1752, 1729, 1633, 1610, 1521, 1447, 1429, 1333, 1272, 1233, 1173, 1119, 1068. ^1^H-NMR (DMSO-d_6_, 600 MH_Z_): *δ* 9.93 (s, 1H, N–OH, exchangeable with D_2_O), 7.48 (dd, 1H, *J* = 2.5 Hz, 14.9 Hz, phenyl H), 7.17 (dd, 1H, *J* = 2.3 Hz, 8.7 Hz, phenyl H), 7.06 (t, 1H, *J* = 8.4 Hz, phenyl H), 4.85–4.90 (br. m, 1H, oxazolidinone H), 4.13 (t, 1H, *J* = 8.9 Hz, oxazolidinone H), 4.05 (dd, 1H, *J* = 6.5 Hz, 13.7 Hz, oxazolidinone H), 3.72–3.76 (m, 5H, morpholine H and methylene H), 3.67 (dd, 1H, *J* = 4.5 Hz, 14.6 Hz, methylene H), 2.96 (t, 4H, *J* = 4.6 Hz, morpholine H), 2.25–2.30 (m, 2H, NCOCH_2_CH(CH_3_)_2_), 1.99–2.04 (m, 1H, NCOCH_2_CH(CH_3_)_2_), 0.88 (t, 6H, *J* = 5.9 Hz, NCOCH_2_CH(CH_3_)_2_). ^13 ^C-NMR (DMSO-d_6_, 600 MH_Z_): *δ* 173.22, 154.55 (d, *J* = 243.45 Hz), 153.95, 135.53 (d, *J* = 8.77 Hz), 133.43 (d, *J* = 10.83 Hz), 119.24 (d, *J* = 4.11 Hz), 114.11 (d, *J* = 2.79 Hz), 106.66 (d, *J* = 26.24 Hz), 69.87, 66.13, 50.69, 50.27, 47.48, 24.42, 22.48. MS ES+ (*m/z*): 396.1874 (M^+^ + H), MS (*m/z*): 395.2 (M^+^). Anal. Calcd. for C_19_H_26_FN_3_O_5_: C: 57.71, H: 6.63, N: 10.63; found C: 57.28, H: 6.94, N: 10.32.

##### 4.1.4.2. (R)-N-((3-(3-fluoro-4-morpholinophenyl)-2-oxooxazolidin-5-yl)methyl)-N-hydroxypentanamide (PH-241)

The intermediate compound 0**PH-240**, was prepared via the general procedure from compound **15** (2.20 g, 7.07 mmol), valeric anhydride (4.18 ml, 21.20 mmol), triethylamine (5.94 ml; 42.40 mmol) in anhydrous DCM (30 ml) to give crude product. Purification by silica gel column chromatography (EtOAc-Hexane, 1:2 to 1:1) gave the intermediate (*R*)-*N*-((3-(3-fluoro-4-morpholinophenyl)-2-oxooxazolidin-5-yl)methyl)-*N*-(pentanoyloxy)pentamide **PH-240** as a white solid 1.10 g, yield 33%, mp 75–77.5 °C. IR (KBr, cm^−1^): *ν* 2963, 2930, 2857, 1797, 1739, 1683, 1627, 1572, 1518, 1447, 1409, 1327, 1236, 1214, 1138, 1120, 1057. ^1^H-NMR (DMSO-d_6_, 600 MH_Z_): *δ* 7.48 (dd, 1H, *J* = 2.5 Hz, 14.9 Hz, phenyl H), 7.18 (dd, 1H, *J* = 2.2 Hz, 8.8 Hz, phenyl H), 7.07 (t, 1H, *J* = 9.4 Hz, phenyl H), 4.84–4.88 (br. m, 1H, oxazolidinone H), 4.10–4.19 (br. t, 2H, oxazolidinone H, overlaps with oxazolidinone H triplet, at 4.11 ppm, *J* = 9.0 Hz), 3.84–3.92 (br., 1H, methylene H), 3.74 (t, 5H, *J* = 4.6 Hz, morpholine H and methylene H), 2.96 (t, 4H, *J* = 4.6 Hz, morpholine H), 2.50 (br., 2H, methylene –CH_2_– overlapping with DMSO signal), 2.11–2.27 (br., 2H, methylene H), 1.56–1.61 (m, 2H, methylene H), 1.40–1.50 (br., 2H, methylene –CH_2_–), 1.31–1.37 (m, 2H, methylene H) 1.20–1.29 (br., 2H, methylene H), 0.88 (t, 3H, *J* = 7.4 Hz, methyl H), 0.82 (br., t, 6H, *J* = 6.5 Hz, methyl H). ^13 ^C-NMR (DMSO-d_6_, 600 MH_Z_): *δ* 154.56 (d, *J* = 243.26 Hz), 153.75, 135.56 (d, *J* = 8.72 Hz), 133.36 (d, *J* = 10.62 Hz), 119.24 (d, *J* = 4.16 Hz), 114.13 (d, *J* = 2.78 Hz), 106.66 (d, *J* = 25.88 Hz), 70.16, 66.13, 50.69, 50.67, 47.15, 30.85, 30.75, 25.97, 21.55, 21.49, 13.62, 13.49. MS ES+ (*m/z*): 480.2300 (M^+^ + H), MS (*m/z*): 479.3 (M^+^). Anal. Calcd. for C_24_H_34_FN_3_O_6_: C: 60.11; H: 7.15; N: 8.76; found C: 60.06; H, 6.87; N, 8.74. A solution of the intermediate **PH-240** (0.900 g, 1.88 mmol) in MeOH:THF (28:7 ml) was treated with NaOH solution (150 mg in 20 ml water). Purification by recrystallisation (EtOAc-hexane 2:1) gave the titled compound **PH-241** as an off-white solid 700 mg, yield 94%, mp 121–122.5 °C. IR (KBr, cm^−1^): *ν* 3187, 2959, 2931, 2860, 1743, 1720, 1626, 1522, 1447, 1425, 1331, 1271, 1234, 1196, 1115, 1071. ^1^H-NMR (DMSO-d_6_, 600 MH_Z_): *δ* 9.94 (s, 1H, N–OH, exchangeable with D_2_O), 7.48 (dd, 1H, *J* = 2.5 Hz, 15.0 Hz, phenyl H), 7.17 (dd, 1H, *J* = 2.3 Hz, 8.9 Hz, phenyl H), 7.06 (t, 1H, *J* = 9.4 Hz, phenyl H), 4.85–4.89 (br. m, 1H, oxazolidinone H), 4.12 (t, 1H, *J* = 8.9 Hz, oxazolidinone H), 4.04 (dd, 1H, *J* = 6.6 Hz, 15.6 Hz, oxazolidinone H), 3.72–3.76 (m, 5H, morpholine H and methylene H), 3.67 (dd, 1H, *J* = 4.6 Hz, 14.9 Hz, methylene H), 2.96 (t, 4H, *J* = 4.7 Hz, morpholine H), 2.36–2.39 (m, 2H, NCOCH_2_CH_2_CH_2_CH_3_), 1.44–1.48 (m, 2H, NCOCH_2_CH_2_CH_2_CH_3_), 1.24–1.30 (m, 2H, NCOCH_2_CH_2_CH_2_CH_3_), 0.85 (t, 2H, *J* = 7.4 Hz, NCOCH_2_CH_2_CH_2_CH_3_). ^13 ^C-NMR (DMSO-d_6_, 600 MH_Z_): *δ* 173.93, 154.53 (d, *J* = 244.06 Hz), 153.90, 135.49 (d, *J* = 8.78 Hz), 133.40 (d, *J* = 10.79 Hz), 119.20 (d, *J* = 4.06 Hz), 114.10 (d, *J* = 2.84 Hz), 106.64 (d, *J* = 26.00 Hz), 69.87, 66.09, 50.66, 50.64, 50.30, 47.45, 31.22, 26.21, 21.81, 13.68. MS ES+ (*m/z*): 396.2037 (M^+^ + H), MS (*m/z*): 395.2 (M^+^). Anal. Calcd. for C_19_H_26_FN_3_O_5_: C: 57.71, H: 6.63, N: 10.63; found C: 58.10, H: 6.61, N: 10.39.

##### 4.1.4.3. (R)-N-((3-(3-fluoro-4-morpholinophenyl)-2-oxooxazolidin-5-yl)methyl)-N-hydroxycyclobutanecarboxamide (PH-245)

The intermediate compound **PH-242** was prepared via the general procedure from compound **15** (2.20 g, 7.07 mmol), cyclobutanecarbonylchloride (2.42 ml, 21.20 mmol), triethylamine (5.94 ml; 42.40 mmol) in anhydrous DCM (30 ml) to give a brown-yellowish gummy crude product. Purification by silica gel column chromatography (EtOAc-Hexane, 1:2 to 1:1) gave the intermediate (*R*)-*N*-((cyclobutanecarbonyl)oxy)-*N*-((3-(3-fluoro-4-morpholino phenyl) −2-oxooxazolidin-5-yl)methyl)cyclobutanecarboxamide **PH-242** as a white solid 0.487 g, yield 15%, mp 122–124 °C. IR (KBr, cm^−1^): *ν* 2963, 2857, 1788, 1741, 1678, 1517, 1445, 1410, 1329, 1261, 1096, 1021. ^1^H-NMR (DMSO-d_6_, 600 MH_Z_): *δ* 7.48 (dd, 1H, *J* = 2.4 Hz, 14.9 Hz, phenyl H), 7.17 (dd, 1H, *J* = 2.3 Hz, 8.8 Hz, phenyl H), 7.07 (t, 1H, *J* = 9.3 Hz, phenyl H), 4.83–4.87 (br. m, 1H, oxazolidinone H), 4.09–4.15 (br. t, 2H, oxazolidinone H, overlaps with oxazolidinone H, triplet, at 4.10 ppm, *J* = 9.0 Hz), 3.87–3.90 (br., 1H, methylene H), 3.73–3.75 (m, 5H, morpholine H and methylene H), 3.32–3.35 (br., 1H, cyclobutane H), 3.09–3.19 (br., 1H, cyclobutane H), 2.96 (t, 4H, *J* = 4.7 Hz, morpholine H), 1.17–2.34 (m, 12H, cyclobutane H). ^13 ^C-NMR (DMSO-d_6_, 600 MH_Z_): *δ* 154.54 (d, *J* = 243.26 Hz), 153.75, 135.57 (d, *J* = 8.72 Hz), 133.34 (d, *J* = 10.75 Hz), 119.23 (d, *J* = 4.13 Hz), 114.16, 106.69 (d, *J* = 26.26 Hz), 70.20, 66.12, 50.68, 50.67, 47.15, 34.94, 24.43, 24.32, 24.21, 24.11, 17.99, 17.44. MS ES+ (*m/z*): 498.2177 (M^+^ + Na), MS (*m/z*): 475.2 (M^+^). A solution of the intermediate **PH-242** (0.487 g, 1.02 mmol) in MeOH:THF (28:7 ml) was treated with NaOH solution (84 mg in 20 ml water). Purification by recrystallisation (EtOAc-hexane 2:1) gave the titled compound **PH-245** as off-white solid 248 mg, yield 92%, mp 132–133.5 °C. IR (KBr, cm^−1^): *ν* 3224, 2953, 2858, 1740, 1722, 1629, 1523, 1476, 1425, 1331, 1233, 1198, 1114, 1081. ^1^H-NMR (DMSO-d_6_, 600 MH_Z_): *δ* 9.80 (s, 1H, N–OH, exchangeable with D_2_O), 7.49 (dd, 1H, *J* = 2.5 Hz, 14.9 Hz, phenyl H), 7.18 (dd, 1H, *J* = 2.3 Hz, 8.9 Hz, phenyl H), 7.06 (t, 1H, *J* = 9.4 Hz, phenyl H), 4.84–4.89 (br. m, 1H, oxazolidinone H), 4.12 (t, 1H, *J* = 8.9 Hz, oxazolidinone H), 4.02 (dd, 1H, *J* = 6.8 Hz, 14.6 Hz, oxazolidinone H), 3.73–3.76 (m, 5H, morpholine H and methylene H), 3.66 (dd, 1H, *J* = 4.5 Hz, 14.6 Hz, methylene H), 3.46–3.52 (m, 1H, cyclobutane H), 2.96 (t, 4H, *J* = 4.7 Hz, morpholine H), 1.75–1.93 (m, 6H, cyclobutane H). ^13 ^C-NMR (DMSO-d_6_, 600 MH_Z_): *δ* 175.29, 155.55 (d, *J* = 243.92 Hz), 153.95, 135.52 (d, *J* = 8.77 Hz), 133.42 (d, *J* = 10.69 Hz), 119.23 (d, *J* = 3.77 Hz), 114.11 (d, *J* = 2.89 Hz), 106.66 (d, *J* = 26.03 Hz), 69.80, 66.13, 50.69, 50.67, 50.56, 47.50, 35.72, 24.39, 17.73. MS ES+ (*m/z*): 394.1899 (M^+^ + H), MS (*m/z*): 393.2 (M^+^). Anal. Calcd. for C_19_H_24_FN_3_O_5_: C: 58.01, H: 6.15, N: 10.68; found C: 57.61, H: 6.02, N: 10.42.

##### 4.1.4.4. (R)-N-((3-(3-fluoro-4-morpholinophenyl)-2-oxooxazolidin-5-yl)methyl)-N-hydroxycyclopentanecarboxamide (PH-244)

The intermediate compound **PH-243** was prepared via the general procedure from compound **15** (2.20 g, 7.07 mmol), cyclopentanecarbonylchloride (2.58 ml, 21.20 mmol), triethylamine (5.94 ml; 42.40 mmol) in anhydrous DCM (30 ml) to give a brown-yellowish gummy crude product. Purification by silica gel column chromatography (EtOAc-Hexane, 1:2 to 1:1) gave the intermediate (*R*)-*N*-((cyclopentanecarbonyl)oxy)-*N*-((3-(3-fluoro-4-morpholino phenyl)-2-oxooxazolidin-5-yl)methyl)cyclopentanecarboxamide **PH-243** as a white solid 1.20 g, yield 34%, recrystallised from EtOAc-Et_2_O, mp 114.5–116 °C. IR (KBr, cm^−1^): *ν* 2960, 2853, 1779, 1754, 1671, 1515, 1449, 1415, 1327, 1225, 1193, 1117, 1087. ^1^H-NMR (DMSO-d_6_, 600 MH_Z_): *δ* 7.48 (dd, 1H, *J* = 2.3 Hz, 14.9 Hz, phenyl H), 7.17 (dd, 1H, *J* = 2.3 Hz, 8.9 Hz, phenyl H), 7.07 (t, 1H, *J* = 9.4 Hz, phenyl H), 4.83–4.88 (br. m, 1H, oxazolidinone H), 4.09–4.16 (br. t, 2H, oxazolidinone H, overlaps with oxazolidinone H triplet, at 4.12 ppm, *J* = 9.0 Hz), 3.84–3.95 (br., 1H, methylene H), 3.74 (t, 5H, *J* = 4.6 Hz, morpholine H and methylene H), 2.96 (t, 5H, *J* = 4.6 Hz, morpholine H and cyclopentane H), 2.64–2.79 (br., 1H, cyclopentane H), 1.44–1.96 (m, 16H, cyclopentane H). ^13 ^C-NMR (DMSO-d_6_, 600 MH_Z_): *δ* 154.52 (d, *J* = 243.39 Hz), 153.71, 135.36 (d, *J* = 8.85 Hz), 133.33 (d, *J* = 10.77 Hz), 119.22 (d, *J* = 4.16 Hz), 114.10 (d, *J* = 2.37 Hz), 106.64 (d, *J* = 26.22 Hz), 70.24, 66.10, 50.66, 50.65, 47.15, 29.49, 29.22, 29.08, 29.05, 25.53, 25.26. MS ES+ (*m/z*): 504.2700 (M^+^ + H), MS (*m/z*): 503.2 (M^+^). Anal. Calcd. for C_26_H_34_FN_3_O_6_: C: 62.01; H: 6.81; N: 8.34; found C: 62.25; H: 6.64; N: 8.08. A solution of the intermediate **PH-243** (0.900 g, 1.79 mmol) in MeOH:THF (28:7 ml) was treated with NaOH solution (143 mg in 20 ml water). Purification by recrystallisation (EtOAc-hexane 2:1) gave the titled compound **PH-244** as off-white solid 578 mg, yield 76%, recrystallised from EtOAc-Et_2_O; mp 164.5–166.5 °C. IR (KBr, cm^−1^): *ν* 3301, 2964, 2869, 1752, 1655, 1640, 1520, 1477, 1444, 1398, 1328, 1239, 1132, 1107. ^1^H-NMR (DMSO-d_6_, 600 MH_Z_): *δ* 9.90 (s, 1H, N–OH, exchangeable with D_2_O), 7.49 (dd, 1H, *J* = 2.5 Hz, 15.00 Hz, phenyl H), 7.18 (dd, 1H, *J* = 2.3 Hz, 8.8 Hz, phenyl H), 7.06 (t, 1H, *J* = 9.4 Hz, phenyl H), 4.84–4.89 (br. m, 1H, oxazolidinone H), 4.13 (t, 1H, *J* = 8.9 Hz, oxazolidinone H), 4.02 (dd, 1H, *J* = 6.5 Hz, 14.2 Hz, oxazolidinone H), 3.73–3.76 (m, 5H, morpholine H and methylene H), 3.68 (dd, 1H, *J* = 4.2 Hz, 14.6 Hz, methylene H), 3.10–3.20 (m, 1H, cyclopentane H), 2.96 (t, 4H, *J* = 4.6 Hz, morpholine H), 1.45–1.80 (m, 8H, cyclopentane H). ^13 ^C-NMR (DMSO-d_6_, 600 MH_Z_): *δ* 176.77, 154.55 (d, *J* = 243.43 Hz), 153.96, 135.51 (d, *J* = 8.75 Hz), 133.43 (d, *J* = 10.72 Hz), 119.23 (d, *J* = 4.18 Hz), 114.10 (d, *J* = 2.87 Hz), 106.64 (d, *J* = 26.14 Hz), 69.90, 66.13, 50.69, 50.68, 50.58, 47.48, 29.27, 29.20, 25.58, 25.56. MS ES+ (*m/z*): 408.2039 (M^+^ + H), MS (*m/z*): 407.2 (M^+^). Anal. Calcd. for C_20_H_26_FN_3_O_5_: C, 58.96; H, 6.43; N, 10.31; found C, 58.57; H, 6.31; N, 10.01.

##### 4.1.4.5. (R)-N-((3-(3-fluoro-4-morpholinophenyl)-2-oxooxazolidin-5-yl) methyl)-N-hydroxyhexanamide (PH-247)

The intermediate compound **PH-246** was prepared via the general procedure from compound **15** (6.0 g, 19.27 mmol), hexanoyl chloride (8.08 ml, 57.82 mmol), triethylamine (16.20 ml; 115.64 mmol) in anhydrous DCM (90 ml) to give crude product. Purification by silica gel column chromatography (EtOAc-Hexane, 1.3:2) gave the intermediate (*R*)-*N*-((3-(3-flouro-4-morpholino phenyl)methyl)-*N*-(hexanoyloxy)-hexanamide **PH-246** as a white solid 4.50 g, yield 46%, mp 80–82.8 °C. IR (KBr, cm^−1^): *ν* 2958, 2930, 2856, 1794, 1740, 1685, 1517, 1446, 1408, 1329, 1237, 1216, 1140, 1119, 1063. ^1^H-NMR (DMSO-d_6_, 600 MH_Z_): *δ* 7.48 (dd, 1H, *J* = 2.5 Hz, 14.9 Hz, phenyl H), 7.18 (dd, 1H, *J* = 2.3 Hz, 8.8 Hz, phenyl H), 7.06 (t, 1H, *J* = 9.3 Hz, phenyl H), 4.84–4.88 (br. m, 1H, oxazolidinone H), 4.10–4.18 (br. t, 2H, oxazolidinone H, overlaps with oxazolidinone H triplet at 4.11, *J* = 9.0 Hz), 3.84–3.94 (br., 1H, methylene H), 3.74 (t, 5H, *J* = 4.6 Hz, morpholine H and methylene H), 2.96 (t, 4H, *J* = 4.6 Hz, morpholine H), 2.50 (br., 2H, methylene –CH_2_– overlapping with DMSO signal), 2.10–2.28 (br., 2H, methylene H), 1.16–1.64 (m, 12H, methylene H), 0.82–0.88 (m, 6H, two methyl H). ^13 ^C-NMR (DMSO-d_6_, 600 MH_Z_): *δ* 154.49 (d, *J* = 246.37 Hz), 153.67, 135.50 (d, *J* = 8.78 Hz), 133.27 (d, *J* = 10.43 Hz), 119.17 (d, *J* = 4.13 Hz), 114.10 (d, *J* = 2.71 Hz), 106.64 (d, *J* = 26.06 Hz), 70.09, 66.06, 50.62, 50.61, 47.11, 31.07, 30.94, 30.53, 30.40, 23.50, 21.68, 21.57, 13.64, 13.60. MS ES+ (*m/z*): 508.3000 (M^+^ + H), MS (*m/z*): 507.3 (M^+^). Anal. Calcd. for C_26_H_38_FN_3_O_6_: C: 61.52; H: 7.55; N: 8.28; found C: 61.44; H, 7.53; N, 7.95. A solution of the intermediate compound **PH-246** (4.50 g, 8.87 mmol) in MeOH:THF (84:21 ml) was treated with NaOH solution (709 mg in 20 ml water). Purification by recrystallisation (EtOAc-hexane 2:1) gave the titled compound **PH-247** as an off-white solid 3.23 g, yield 89%, mp 118.5–120.5 °C. IR (KBr, cm^−1^): *ν* 3187, 2957, 2930, 2858, 1745, 1719, 1626, 1524, 1475, 1426, 1332, 1270, 1258, 1234, 1196, 1114, 1073. ^1^H-NMR (DMSO-d_6_, 600 MH_Z_): *δ* 9.92 (s, 1H, N–OH, exchangeable with D_2_O), 7.48 (dd, 1H, *J* = 2.5 Hz, 15.0 Hz, phenyl H), 7.17 (dd, 1H, *J* = 2.2 Hz, 8.8 Hz, phenyl H), 7.06 (t, 1H, *J* = 9.4 Hz, phenyl H), 4.85–4.89 (br. m, 1H, oxazolidinone H), 4.12 (t, 1H, *J* = 8.9 Hz, oxazolidinone H), 4.04 (dd, 1H, *J* = 6.7 Hz, 14.7 Hz, oxazolidinone H), 3.73–3.76 (m, 5H, morpholine H and methylene H), 3.67 (dd, 1H, *J* = 4.3 Hz, 14.8 Hz, methylene H), 2.96 (t, 4H, *J* = 4.7 Hz, morpholine H), 2.35–2.38 (m, 2H, NCOCH_2_CH_2_CH_2_CH_2_CH_3_), 1.45–1.49 (m, 2H, NCOCH_2_CH_2_CH_2_CH_2_CH_3_), 1.22–1.30 (m, 4H, NCOCH_2_CH_2_CH_2_CH_2_CH_3_), 0.85 (t, 3H, *J* = 7.0 Hz, NCOCH_2_CH_2_CH_2_CH_2_CH_3_). ^13 ^C-NMR (DMSO-d_6_, 600 MH_Z_): *δ* 173.90, 154.51 (d, *J* = 244.10 Hz), 153.88, 135.46 (d, *J* = 8.79 Hz), 133.38 (d, *J* = 10.70 Hz), 119.17 (d, *J* = 4.18 Hz), 114.07 (d, *J* = 2.64 Hz), 106.62 (d, *J* = 26.24 Hz), 69.86, 66.07, 50.64, 50.62, 50.26, 47.42, 31.46, 30.88, 23.70, 21.80, 13.71. MS ES+ (*m/z*): 410.2000 (M^+^ + H), MS (*m/z*): 409.2 (M^+^). Anal. Calcd. for C_20_H_28_FN_3_O_5_: C: 58.67; H: 6.89; N: 10.26; found C: 58.32, H: 6.87, N: 10.61.

##### 4.1.4.6. (R)-N-((3-(3-fluoro-4-morpholinophenyl)-2-oxooxazolidin-5-yl) methyl)-N-hydroxyheptanamide (PH-249)

The intermediate compound **PH-248** was prepared via the general procedure from compound **15** (6.00 g, 19.27 mmol), heptanoyl chloride (8.95 ml, 57.81 mmol), triethylamine (16.21 ml; 115.62 mmol) in anhydrous DCM (30 ml) to give crude product. Purification by silica gel column chromatography (EtOAc-Hexane, 3:4) gave the intermediate (*R*)-*N*-(3-(3-fluoro-4-morpholinophenyl)-2-oxooxazolidin-5-yl) methyl)-*N*-(heptanoyloxy) heptanamide **PH-248** as a white solid 6.82 g, yield 66%, mp 68–70.5 °C. IR (KBr, cm^−1^): *ν* 2960, 2929, 2855, 1794, 1740, 1685, 1519, 1446, 1410, 1329, 1236, 1217, 1140, 1119, 1066. ^1^H-NMR (DMSO-d_6_, 600 MH_Z_): *δ* 7.48 (dd, 1H, *J* = 2.5 Hz, 14.7 Hz, phenyl H), 7.17 (dd, 1H, *J* = 2.2 Hz, 8.8 Hz, phenyl H), 7.06 (t, 1H, *J* = 9.3 Hz, phenyl H), 4.84–4.88 (br. m, 1H, oxazolidinone H), 4.10–4.18 (br. t, 2H, oxazolidinone H, overlaps with oxazolidinone H triplet at 4.11, *J* = 9.0 Hz), 3.84–3.94 (br., 1H, methylene H), 3.73 (t, 5H, *J* = 4.6 Hz, morpholine H and methylene H), 2.96 (t, 4H, *J* = 4.6 Hz, morpholine H), 2.50 (br., 2H, methylene –CH_2_– overlapping with DMSO signal), 2.10–2.26 (br., 2H, methylene H), 1.15–1.64 (m, 16H, methylene H), 0.86–0.87 (m, 6H, two methyl H). ^13 ^C-NMR (DMSO-d_6_, 600 MH_Z_): *δ* 154.53 (d, *J* = 244.26 Hz), 153.72, 135.54 (d, *J* = 8.77 Hz), 133.31 (d, *J* = 10.73 Hz), 119.19 (d, *J* = 4.01 Hz), 114.11 (d, *J* = 2.43 Hz), 106.66 (d, *J* = 26.01 Hz), 70.13, 66.10, 50.66, 50.65, 47.13, 31.03, 30.87, 30.74, 28.04, 27.94, 23.83, 21.87, 21.83, 13.79. MS ES+ (*m/z*): 535.3498 (M^+^ + H), 538.3384 (M^+^ + Na), MS (*m/z*): 535.4 (M^+^). Anal. Calcd. for C_28_H_42_FN_3_O_6_: C: 62.78; H: 7.90; N: 7.84; found C: 62.72; H, 7.90; N, 7.51. A solution of the intermediate **PH-248** (6.28 g, 11.72 mmol) in MeOH:THF (84:21 ml) was treated with NaOH solution (928 mg in 20 ml water). Purification by recrystallisation (EtOAc-hexane 2:1) gave the titled compound **PH-249** as an off-white solid 4.30 g, yield 87%, mp 123–125 °C. IR (KBr, cm^−1^): *ν* 3188, 2957, 2923, 2855, 1743, 1719, 1626, 1525, 1473, 1426, 1332, 1271, 1235, 1196, 1115, 1073. ^1^H-NMR (DMSO-d_6_, 600 MH_Z_): *δ* 9.92 (s, 1H, N–OH, exchangeable with D_2_O), 7.48 (dd, 1H, *J* = 2.5 Hz, 14.9 Hz, phenyl H), 7.18 (dd, 1H, *J* = 2.2 Hz, 8.8 Hz, phenyl H), 7.06 (t, 1H, *J* = 9.3 Hz, phenyl H), 4.85–4.89 (br. m, 1H, oxazolidinone H), 4.12 (t, 1H, *J* = 8.9 Hz, oxazolidinone H), 4.04 (dd, 1H, *J* = 6.7 Hz, 14.7 Hz, oxazolidinone H), 3.73–3.75 (m, 5H, morpholine H and methylene H), 3.67 (dd, 1H, *J* = 4.2 Hz, 14.9 Hz, methylene H), 2.96 (t, 4H, *J* = 4.7 Hz, morpholine H), 2.35–2.38 (m, 2H, NCOCH_2_CH_2_CH_2_CH_2_CH_2_CH_3_), 1.44–1.49 (m, 2H, NCOCH_2_CH_2_CH_2_CH_2_CH_2_CH_3_), 1.21–1.27 (m, 6H, NCOCH_2_CH_2_CH_2_CH_2_CH_2_CH_3_), 0.85 (t, 3H, *J* = 7.0 Hz, NCOCH_2_CH_2_CH_2_CH_2_CH_2_CH_3_). ^13 ^C-NMR (DMSO-d_6_, 600 MH_Z_): *δ* 173.93, 154.53 (d, *J* = 244.01 Hz), 153.92, 135.49 (d, *J* = 8.73 Hz), 133.41 (d, *J* = 10.27 Hz), 119.19 (d, *J* = 4.15 Hz), 114.07 (d, *J* = 2.80 Hz), 106.63 (d, *J* = 26.27 Hz), 69.90, 66.10, 50.67, 50.65, 50.27, 47.43, 31.54, 31.02, 28.36, 24.02, 21.91, 13.85. MS ES+ (*m/z*): 424.2521 (M^+^ + H), MS (*m/z*): 423.3 (M^+^). Anal. Calcd. for C_21_H_30_FN_3_O_5_: C: 59.56; H: 7.14; N: 9.92; found C: 59.58, H: 7.52, N: 10.05.

##### 4.1.4.7. (R)-N-((3-(3-fluoro-4-morpholinophenyl)-2-oxooxazolidin-5-yl) methyl)-N-hydroxyoctanamide (PH-251)

The intermediate compound **PH-250** was prepared via the general procedure from compound **15** (2.43 g, 7.80 mmol), octanoyl chloride (3.27 ml, 23.40 mmol), triethylamine (6.60 ml; 46.80 mmol) in anhydrous DCM (35 ml) to give crude product. Purification by silica gel column chromatography (EtOAc-hexane, 1:2; 3:4) gave the intermediate(*R*)-*N*-((3-(-Fluoro-4-morpholinophenyl)-2-oxooxazolidin-5-yl)methyl)-*N*-(octanoyloxy)octanamide **PH-250** as a white solid 2.20 g, yield 50%, mp 49–53 °C. IR (KBr, cm^−1^): *ν* 2954, 2925, 2853, 1790, 1750, 1673, 1520, 1442, 1417, 1377, 1328, 1272, 1225, 1193, 1120, 1075. ^1^H-NMR (DMSO-d_6_, 600 MH_Z_): *δ* 7.48 (dd, 1H, *J* = 2.5 Hz, 14.9 Hz, phenyl H), 7.17 (dd, 1H, *J* = 2.3 Hz, 8.8 Hz, phenyl H), 7.06 (t, 1H, *J* = 9.3 Hz, phenyl H), 4.84–4.88 (br. m, 1H, oxazolidinone H), 4.09–4.18 (br. t, 2H, oxazolidinone H, overlaps with oxazolidinone H triplet at 4.11, *J* = 9.1 Hz), 3.84–3.94 (br., 1H, methylene H), 3.73 (t, 5H, *J* = 4.6 Hz, morpholine H and methylene H), 2.96 (t, 4H, *J* = 4.6 Hz, morpholine H), 2.50 (br., 2H, methylene –CH_2_– overlapping with DMSO signal), 2.10–2.24 (br., 2H, methylene H), 1.20–1.64 (m, 20H, methylene H), 0.83–0.87 (m, 6H, two methyl H). ^13 ^C-NMR (DMSO-d_6_, 600 MH_Z_): *δ* 154.53 (d, *J* = 244.26 Hz), 153.72, 135.56 (d, *J* = 8.77 Hz), 133.31 (d, *J* = 10.72 Hz), 119.18 (d, *J* = 4.01 Hz), 114.08 (d, *J* = 2.43 Hz), 106.66 (d, *J* = 26.01 Hz), 70.14, 66.10, 50.66, 50.65, 47.11, 31.07, 31.03, 28.34, 28.33, 28.25, 28.23, 23.89, 21.97, 13.86. MS ES+ (*m/z*): 564.3386 (M^+^ + H), MS (*m/z*): 563.3 (M^+^). Anal. Calcd. for C_30_H_46_FN_3_O_6_: C: 63.92; H: 8.23; N: 7.45; found C: 64.01; H, 8.26; N, 7.43. A solution of the intermediate **PH-250** (2.15 g, 3.81 mmol) in MeOH:THF (56:14 ml) was treated with NaOH solution (305 mg in 20 ml water). Purification by recrystallisation (EtOAc-hexane 2:1) gave the titled compound **PH-251** as an off-white solid 1.30 g, yield 78%, mp 119–122 °C. IR (KBr, cm^−1^): *ν* 3184, 2956, 2923, 2854, 1743, 1720, 1626, 1524, 1471, 1446, 1427, 1332, 1272, 1235, 1197, 1115, 1075. ^1^H-NMR (DMSO-d_6_, 600 MH_Z_): *δ* 9.93 (s, 1H, N–OH, exchangeable with D_2_O), 7.48 (dd, 1H, *J* = 2.5 Hz, 14.9 Hz, phenyl H), 7.17 (dd, 1H, *J* = 2.2 Hz, 8.8 Hz, phenyl H), 7.06 (t, 1H, *J* = 9.4 Hz, phenyl H), 4.85–4.89 (br. m, 1H, oxazolidinone H), 4.12 (t, 1H, *J* = 8.9 Hz, oxazolidinone H), 4.04 (dd, 1H, *J* = 6.7 Hz, 14.7 Hz, oxazolidinone H), 3.73–3.75 (m, 5H, morpholine H and methylene H), 3.67 (dd, 1H, *J* = 4.2 Hz, 14.9 Hz, methylene H), 2.96 (t, 4H, *J* = 4.6 Hz, morpholine H), 2.34–2.38 (m, 2H, NCOCH_2_CH_2_CH_2_CH_2_CH_2_CH_2_CH_3_), 1.44–1.50 (m, 2H, NCOCH_2_CH_2_CH_2_CH_2_CH_2_CH_2_CH_3_), 1.18–1.30 (m, 8H, NCOCH_2_CH_2_CH_2_CH_2_CH_2_CH_2_CH_3_), 0.85 (t, 3H, *J* = 7.0 Hz, NCOCH_2_CH_2_CH_2_CH_2_CH_2_CH_2_CH_3_). ^13 ^C-NMR (DMSO-d_6_, 600 MH_Z_): *δ* 173.96, 154.56 (d, *J* = 244.06 Hz), 153.95, 135.51 (d, *J* = 8.78 Hz), 133.43 (d, *J* = 10.79 Hz), 119.21 (d, *J* = 4.06 Hz), 114.08 (d, *J* = 2.84 Hz), 106.64 (d, *J* = 26.00 Hz), 69.94, 66.13, 50.69, 50.68, 50.26, 47.44, 31.57, 31.14, 28.70, 28.49, 24.10, 22.04, 13.93.

MS ES+ (*m/z*): Calcd for C_22_H_32_FN_3_O_5_ + H: 438.2404, found: 438.2402 (M^+^ + H), Calcd. for C_22_H_32_FN_3_O_5_ + Na: 460.2224, found: 460.2214 (M^+^ + Na), MS (*m/z*): 437.2 (M^+^). Anal. Calcd. for C_22_H_32_FN_3_O_5_: C: 60.40; H: 7.37; N: 9.60; found C: 60.54; H, 7.10; N, 9.43.

### 4.2. Biological activity

#### 4.2.1. General

The solvents, drugs and other reagents, including, polyethylene glycol (PEG), dimethylsulphoxide (DMSO), *N*-formyl methyl-leucyl-phenylalanine (FMLP), zileuton, zymosan, mouse anti-dinitrophenyl (DNP) IgE, dinitrophenyl-conjugated bovine serum albumin (DNP-BSA), foetal bovine serum (FBS), heparin, calcium ionophore A23187, LPS, DTT and glutathione peroxidase (GPx) were obtained from Sigma-Aldrich (St. Louis, MO). All compounds for *in vitro* experiments were solubilised in DMSO and diluted down in PBS. The final concentration of the solvent did not exceed 0.05%. For *in vivo* experiments, the compounds were made up in drug vehicle (4% DMSO/67.2% PEG/28.8% PBS).

#### 4.2.2. In vitro assay for the inhibition of 5-LO product LTB_4_ from human whole blood

All compounds were evaluated for inhibitory activity against the 5-LO-dependent generation of LTB_4_ from activated human whole blood. With ethical approval from the Health Sciences Centre Ethical Committee of Kuwait University, heparinised fresh human blood samples from apparently healthy individuals of both sexes were obtained from the Kuwait Central Blood Bank. All donors gave informed consent and the work was carried out in accordance with the “Declaration of Helsinki” for experiments involving human subjects. Aliquots of 185 µl of whole blood were dispensed into each well of a 96-well culture plate containing 5 µl of the priming agent, LPS (1 µg/ml final concentration). After a 15 min incubation at 37 °C, 5 µl of the test compounds (0.001–30 µM) or the reference drug, zileuton (as positive control) or the vehicle (0.05% DMSO), was added. After further incubation for 15 min at 37 °C, 5 µl of FMLP (1 µM final concentration) was added to stimulate LT production. The reaction was stopped 15 min later, and the supernatants were recovered by centrifugation and stored at −40 °C pending analysis of LTB_4_ content.

#### 4.2.3. In vitro assay for the inhibition of 5-LO product LTC_4_ from isolated human monocytes

Mononuclear cells were first isolated from heparinised blood using the Ficoll-Hypaque gradient centrifugation method. Monocytes were subsequently purified by adherence to plastic according to standard protocols. The purity of the adherent monocytes (CD14+) was routinely confirmed by flow cytometry to be >95% and viability (by trypan blue exclusion method) was routinely >97%. Adherent monocytes were then washed and incubated in 190 µl culture medium RPMI-1640 supplemented with 100 U/ml penicillin, 100 µg/ml streptomycin and 10% heat-inactivated foetal bovine serum (Sigma-Aldrich, St Louis, MO). Cells were then incubated with the various test compounds (0.001–30 μM) or vehicle (0.03% DMSO) or zileuton (positive control) for 15 min before being stimulated with 5 µl of the calcium ionophore A23187 at a final concentration of 2.5 µM. After further incubation for 15 min, the culture supernatants were recovered by centrifugation and stored at −40 °C pending LTC_4_ determination.

#### 4.2.4. In vitro assay for the inhibition of 5-LO product LTC_4_ from allergen/IgE-activated bone marrow-derived mouse mast cells (BMMC)

Bone marrow-derived mast cells (BMMC) were generated from pathogen-free 5 to 7-weeks-old male Balb/c mice according to the method of Davis et al.^28^. Essentially, bone marrow cells were obtained by flushing the femoral bone marrow and the recovered cells cultured in RPMI 1640 medium supplemented with 10% FBS, 100 U/ml penicillin, and 100 µg/ml streptomycin, 25 mM HEPES, 1.0 mM sodium pyruvate, 0.1 mM nonessential amino acids, 0.0035% 2-mercaptoethanol, and 30 ng/ml mouse recombinant IL-3, with culture medium, replaced every 2 days. Cells were used after 4–8 weeks of culture, by which time at least 97% of the cells would have differentiated into mast cells, as routinely obtained in our laboratory.

The generated BMMCs were seeded at 5 × 10^4^ cells/well in a 96-well flat-bottom culture plate and passively sensitised overnight with 0.5 µg/ml anti-DNP monoclonal IgE antibody. The cells were then washed twice to remove any unbound antibody and subsequently resuspended in reaction buffer (135 mM NaCl, 5 mM KCl, 1.8 mM CaCl_2_, 1 mM MgCl_2_, 5.6 mM glucose, 0.05% BSA and 20 mM HEPES, pH 7.4). They were then pre-incubated with the test compounds (0.001 − 30 μM) or the solvent (0.05% DMSO) for 15 min before being stimulated with the specific antigen, DNP-BSA (10–30 ng/ml). After 30 min incubation at 37 °C, the amount of LTC_4_ released into the supernatant was determined by ELISA as described below.

#### 4.2.5. Assay of released leukotrienes

Appropriately diluted supernatants were assayed for the released products – LTs (LTB_4_ and LTC_4_), by the enzyme immunoassay (EIA) method using assay kits supplied by R&D Systems (Minneapolis, MN) and following the manufacturer’s instructions.

#### 4.2.6. Evaluation of the direct inhibition of 5-LO activity in a cell-free assay

The assay was based on the oxidation of the dye 2′,7′-dichlorodihydrofluorescein diacetate (H2DCF-DA) to a highly fluorescent product by 5-LO enzymatic products^29^. H2DCF-DA (60 µM) was first pre-cleaved by incubating with 450 mU/ml recombinant human 5-LO enzyme in Tris buffer (containing 50 mM Tris, pH = 7.5, 2 mM EDTA, 2 mM CaCl_2_, 1 mM DTT and 0.6 U/ml glutathione peroxidase) for 10 min at room temperature. Then, to each well of a black 96-well plate was added 25 µl of the above enzyme/dye solution, followed by 25 µl of the test compounds or zileuton (0.001–30 µM) or drug vehicle, in duplicates. After 10 min incubation at room temperature, the reaction was started with the addition of 50 µl of substrate solution (Tris buffer containing 20 µM ATP and 20 µM arachidonic acid (AA). After a further 20 min, the reaction was terminated with 100 µl acetonitrile. Fluorescence was read at 500 nm excitation and 520 nm emission, with Novostar^R^ microplate reader (BMG Labtech, Offenburg, Germany). Appropriate controls, including 100 µM nordihydroguaiaretic acid (NDGA), were included to isolate only the 5-LO-attributable, NDGA-inhibitable RFU values.

#### 4.2.7. In vitro toxicity testing

Adherent human monocytes, prepared as described above were cultured with various concentrations of the test compounds or vehicle, or with 0.05% Triton-X as a positive control for 3 h or 24 h. At the end of the culture, cell viability was determined using the standard MTT assay method. Briefly, 15 μL of the MTT solution (5 mg/ml) was added to each well and incubated at 37 °C for 4 h. After removing the supernatant, 200 μL of DMSO was added to dissolve the crystals. Absorbance at 570 nm was then measured in a microplate reader. Viability was expressed as a percentage of the vehicle-treated cells.

#### 4.2.8. Evaluation of in vivo activity in zymosan-induced peritoneal inflammation model in mice

Female Balb/c mice 6 to 8-weeks-old obtained from the Animal Resources Centre of the Health Sciences Centre, Kuwait University, were used. They were maintained under temperature-controlled conditions with an artificial 12-h light/dark cycle and allowed standard chow and water *ad libitum*. The study was carried out in compliance with the Regulations for the Use of Laboratory Animals in the Health Sciences Centre, Kuwait University, and complied with the National Institute of Health guide for the care and use of laboratory animals (NIH Publication # 8023, revised 1978).

The zymosan-induced peritonitis model - a recognised LT-mediated inflammatory reaction^26^, was used. Five groups of mice, 7 per group, were treated subcutaneously with either **PH-249** (the most active compound on human cells) at doses of 10 or 30 mg/kg or drug vehicle alone or zileuton for comparison. After 30 min, all groups were injected intraperitoneally with 0.2 ml of activated zymosan (2 mg/ml) except the control group that received 0.2 ml PBS. After 2 h, all animals were killed, and the peritoneal exudate collected by washing the cavity with 3 ml of heparinised (10 IU/ml) PBS. Cells in the exudate were recovered by centrifugation and counted in a haemocytometer while the supernatant volume was recorded and then stored frozen at −78 °C until used for the determination of LTC_4_ by ELISA as detailed above.

#### 4.2.9. Statistical analysis

All data were analysed using GraphPad Prism software (GraphPad Software, San Diego, CA). The 50% inhibition concentration (IC_50_) values were determined from the concentration-response curves by non-linear regression analysis (normalised variable). Differences between experimental groups were first analysed by one-way ANOVA, followed by Bonferroni’s post-hoc test. A *p*-value of less than 0.05 was taken as statistically significant.

## Supplementary Material

Supplemental Material
