# Supplementary material for: Synthesis and structure-activity relationships of novel 5-(hydroxamic acid)methyl oxazolidinone derivatives as 5-lipoxygenase inhibitors
Source: J Enzyme Inhib Med Chem. 2020 Jul 8;35(1):1471–82. doi: 10.1080/14756366.2020.1786082 (PMC7470027; doi:10.1080/14756366.2020.1786082)
Supplement: Supplemental Material [file IENZ_A_1786082_SM0436.pdf]

SUPPLEMENTARY DATA

Synthesis and structure-activity relationships of novel 5-(hydroxamic acid)methyl oxazolidinone derivatives as 5-Lipoxygenase inhibitors

Oludotun A. Phillips[a], Mira A. Bosso[b], Charles I. Ezeamuzie[b*]

[a]Department of Pharmaceutical Chemistry, Faculty of Pharmacy, Kuwait University, P. O. Box 24923, Safat 13110, Kuwait.

[b]Department of Pharmacology & Toxicology, Faculty of Medicine, Kuwait University, P.O. Box 24923, Safat 13110, Kuwait.

[*]Author to whom correspondence should be addressed; Tel: +(965) 2463-6070

Fax: +(965) 2463-68940; e-mail: dphillips@hsc.edu.kw, ezeamuzie@hsc.edu.kw,

101-SL

C:\Xcalibur\Data\phillips\MAB16-110-DCI
1/19/2017 12:50:36 PM

GC MS DFS- Thermo
Project No: GS01/03

MAB16-110
MAB-16-101-1

MAB16-110-DCI #93 RT: 4.22 AV: 1 NL: 4.11E5
T: + c EI Full ms [49.50-1000.50]

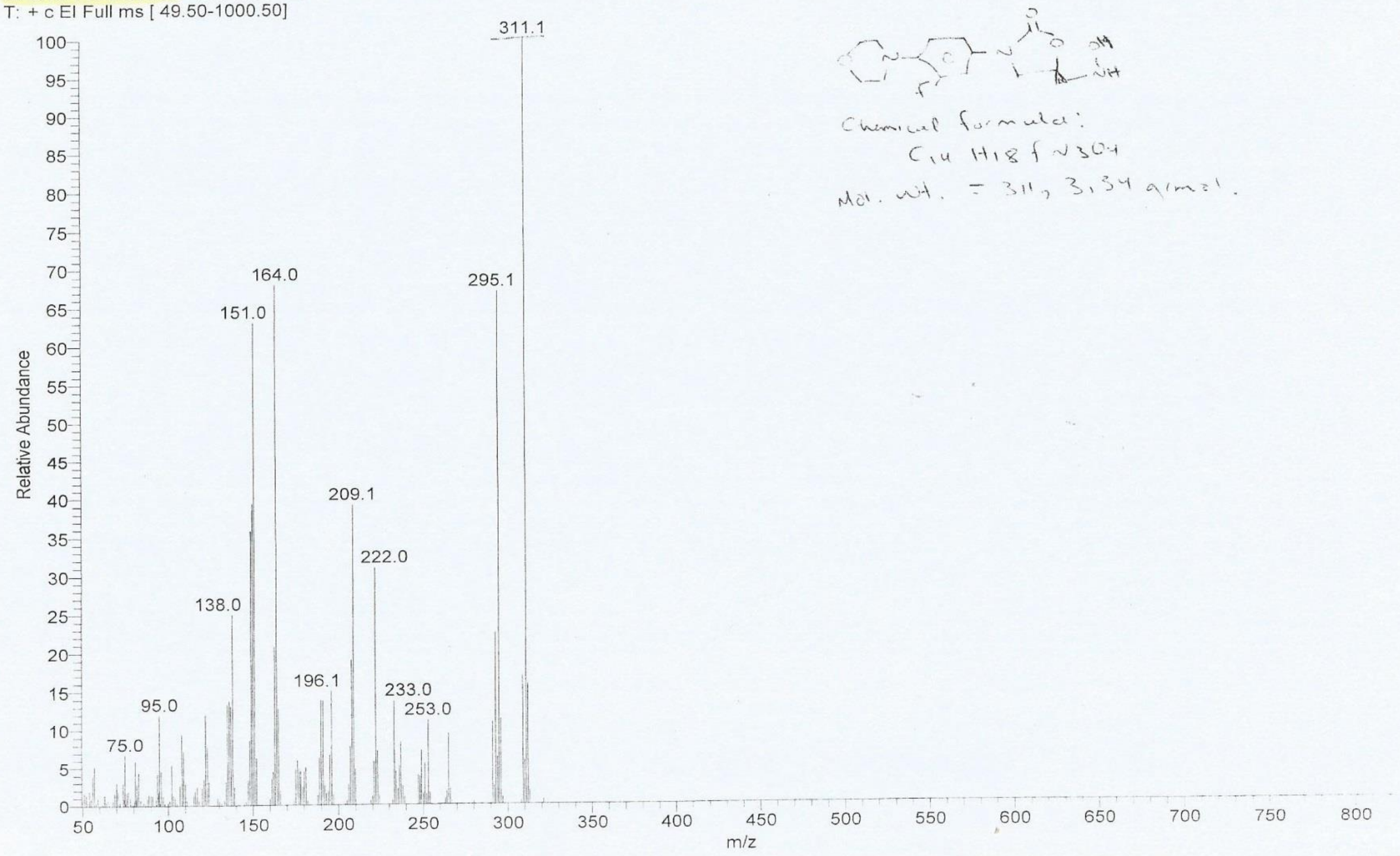

Chemical formula:
$C_{14} H_{18} F N_3 O_4$
Mol. wt. = 311,3,34 g/mol.

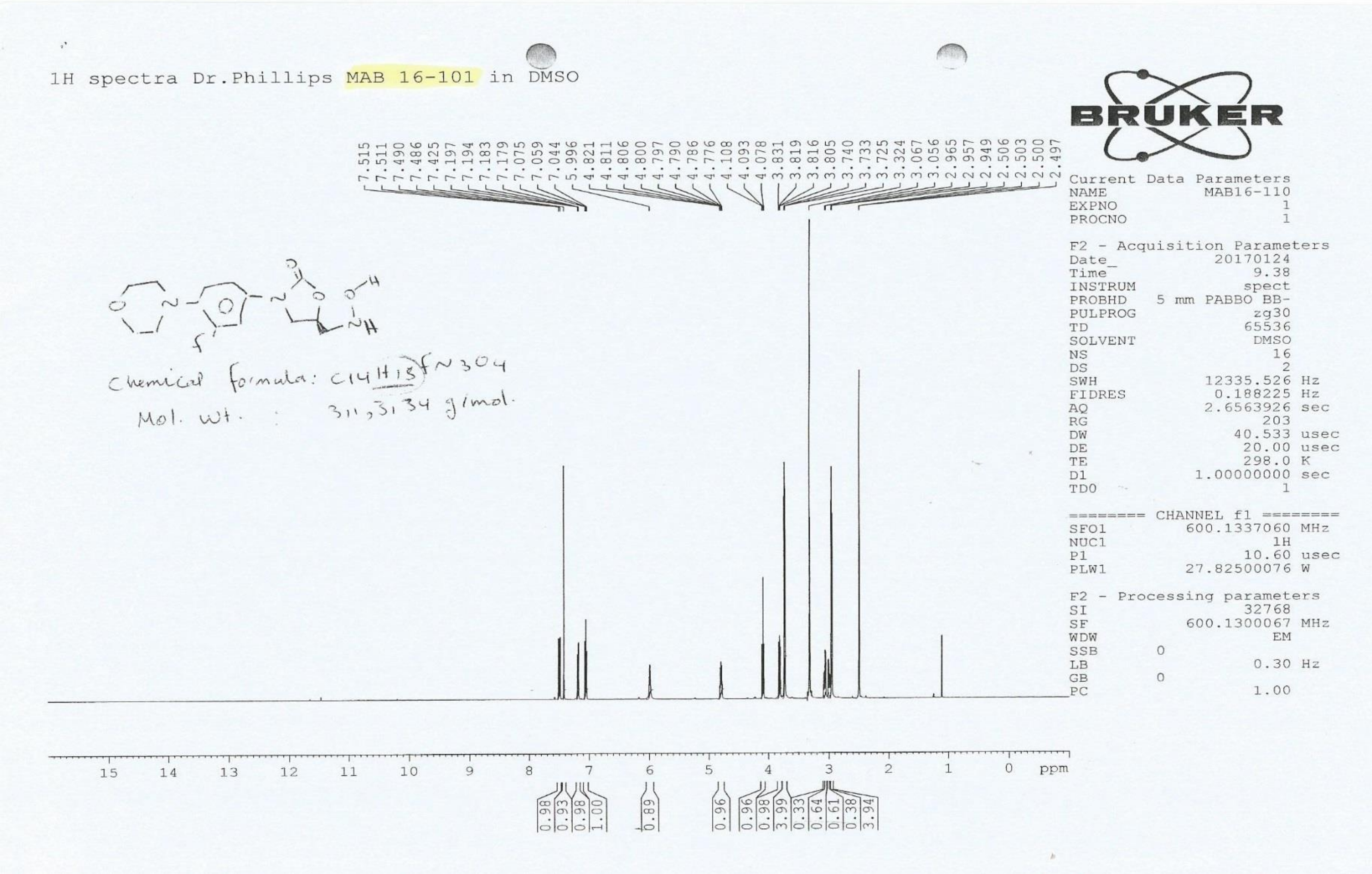

1H spectra Dr.Phillips MAB 16-101 in DMSO
BRUKER
7.515
7.511
7.490
7.486
7.425
7.197
7.194
7.183
7.179
7.075
7.059
7.044
5.996
4.821
4.811
4.806
4.800
4.797
4.790
4.786
4.776
4.108
4.093
4.078
3.831
3.819
3.816
3.805
3.740
3.733
3.725
3.324
3.067
3.056
2.965
2.957
2.949
2.506
2.503
2.500
2.497
Chemical formula: C14H18FN3O4
Mol. wt. : 311,3134 g/mol.
Current Data Parameters
NAME MAB16-110
EXPNO 1
PROCNO 1
F2 - Acquisition Parameters
Date_ 20170124
Time 9.38
INSTRUM spect
PROBHD 5 mm PABBO BB-
PULPROG zg30
TD 65536
SOLVENT DMSO
NS 16
DS 2
SWH 12335.526 Hz
FIDRES 0.188225 Hz
AQ 2.6563926 sec
RG 203
DW 40.533 usec
DE 20.00 usec
TE 298.0 K
D1 1.00000000 sec
TD0 1
======== CHANNEL f1 ========
SFO1 600.1337060 MHz
NUC1 1H
P1 10.60 usec
PLW1 27.82500076 W
F2 - Processing parameters
SI 32768
SF 600.1300067 MHz
WDW EM
SSB 0
LB 0.30 Hz
GB 0
PC 1.00
15 14 13 12 11 10 9 8 7 6 5 4 3 2 1 0 ppm
0.98
0.93
0.98
1.00
0.89
0.96
0.96
0.98
3.99
0.33
0.64
0.61
0.38
3.94

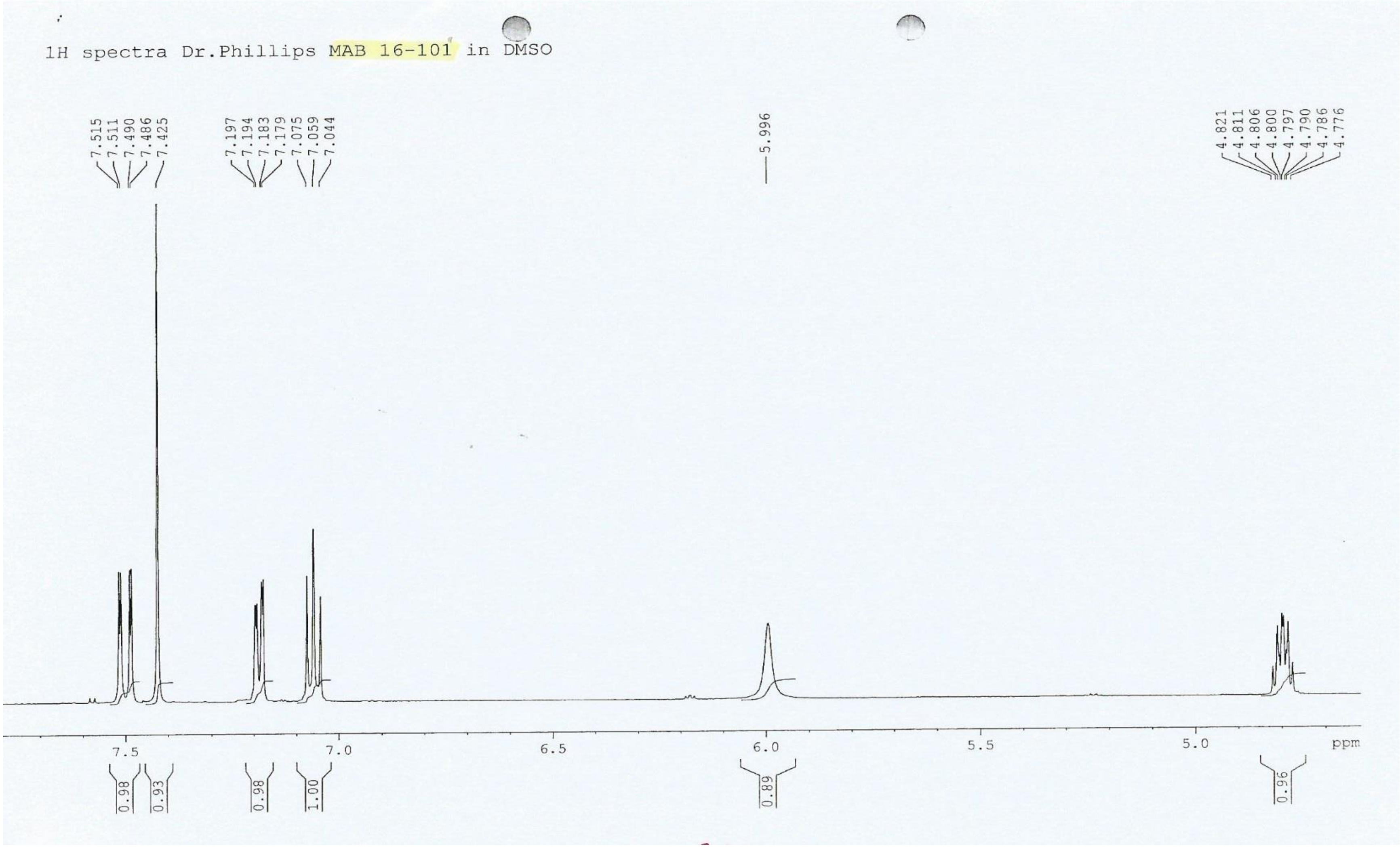
1H spectra Dr.Phillips MAB 16-101 in DMSO
7.515
7.511
7.490
7.486
7.425
7.197
7.194
7.183
7.179
7.075
7.059
7.044
5.996
4.821
4.811
4.806
4.800
4.797
4.790
4.786
4.776
7.5
7.0
6.5
6.0
5.5
5.0
ppm
0.98
0.93
0.98
1.00
0.89
0.96

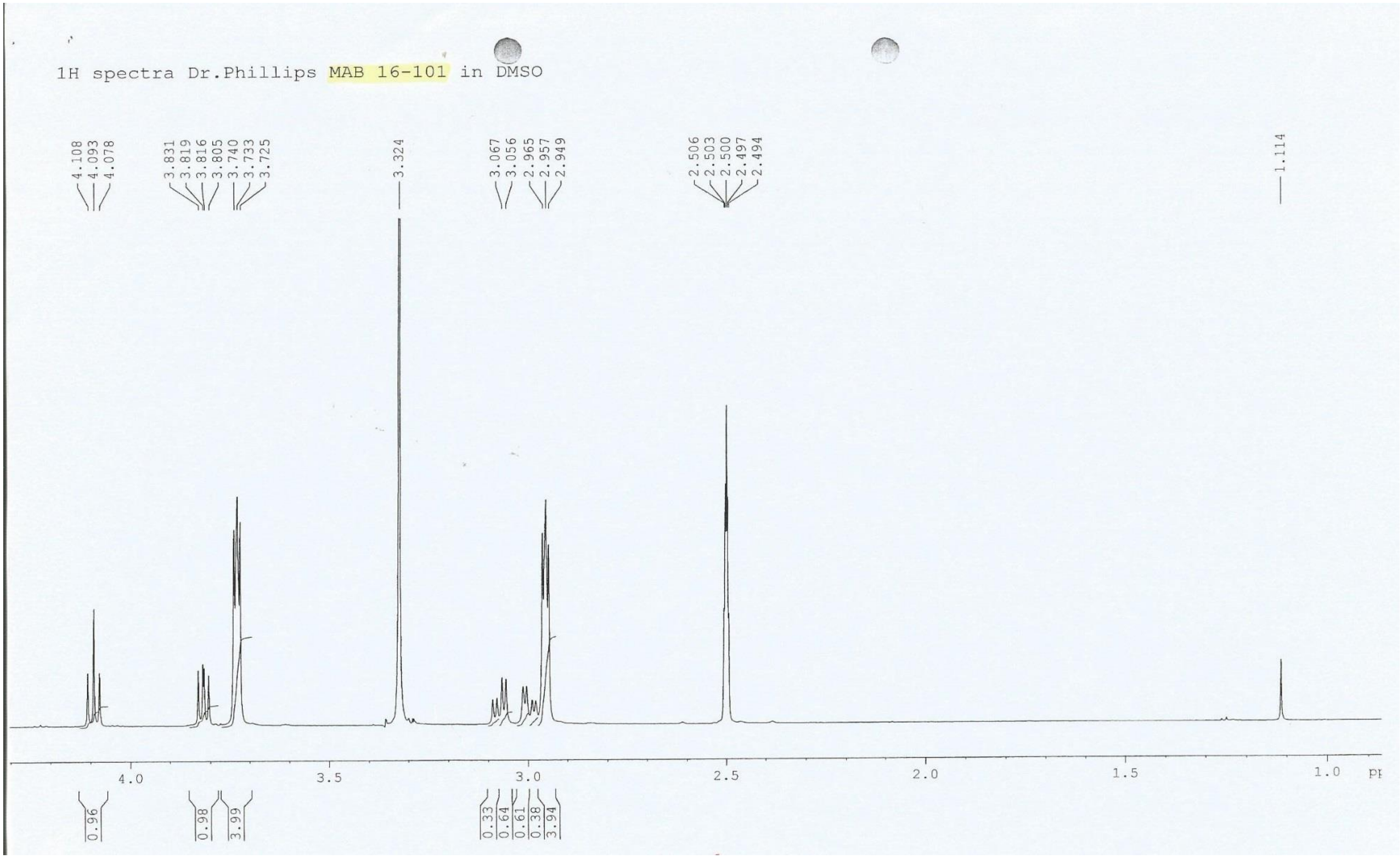
1H spectra Dr.Phillips MAB 16-101 in DMSO
4.108
4.093
4.078
3.831
3.819
3.816
3.805
3.740
3.733
3.725
3.324
3.067
3.056
2.965
2.957
2.949
2.506
2.503
2.500
2.497
2.494
1.114
4.0
3.5
3.0
2.5
2.0
1.5
1.0
0.96
0.98
3.99
0.33
0.64
0.61
0.38
3.94

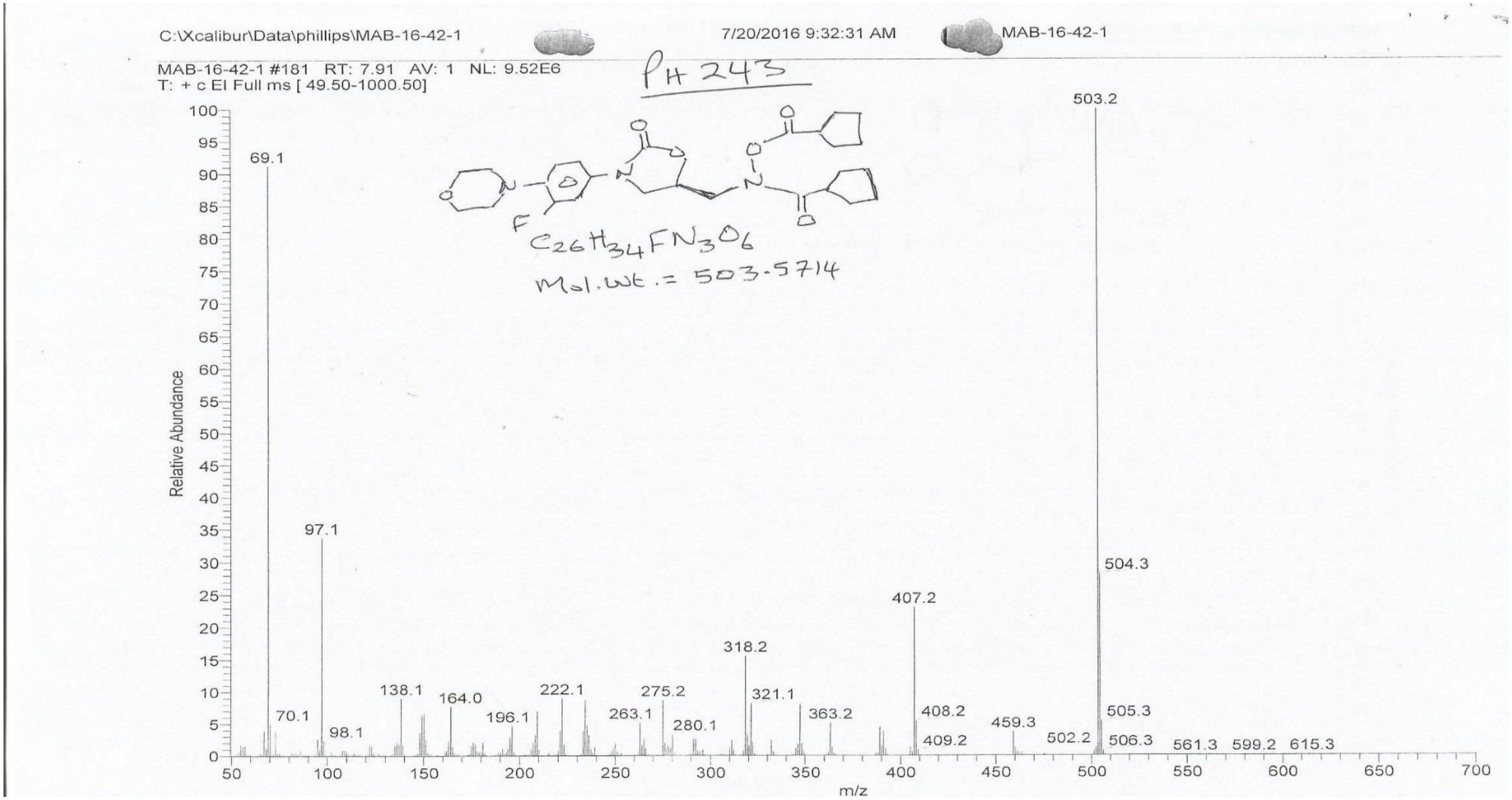
C:\Xcalibur\Data\phillips\MAB-16-42-1
7/20/2016 9:32:31 AM
MAB-16-42-1
MAB-16-42-1 #181 RT: 7.91 AV: 1 NL: 9.52E6
T: + c EI Full ms [49.50-1000.50]
PH 243
F
$C_{26}H_{34}FN_3O_6$
Mol. Wt. = 503.5714
Relative Abundance
m/z
69.1
70.1
97.1
98.1
138.1
164.0
196.1
222.1
263.1
275.2
280.1
318.2
321.1
363.2
407.2
408.2
409.2
459.3
502.2
503.2
504.3
505.3
506.3
561.3
599.2
615.3

[redacted]Research Sector Projects Unit[redacted] 01/05
Kuwait University

RSPU
Research Sector Projects Unit
KUWAIT UNIVERSITY

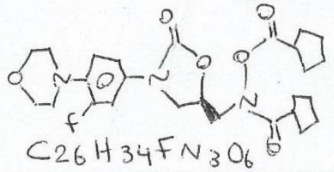

$C_{26}H_{34}FN_3O_6$

FT- IR Spectral Data

Mol. wt = 503.5714 g/mol.

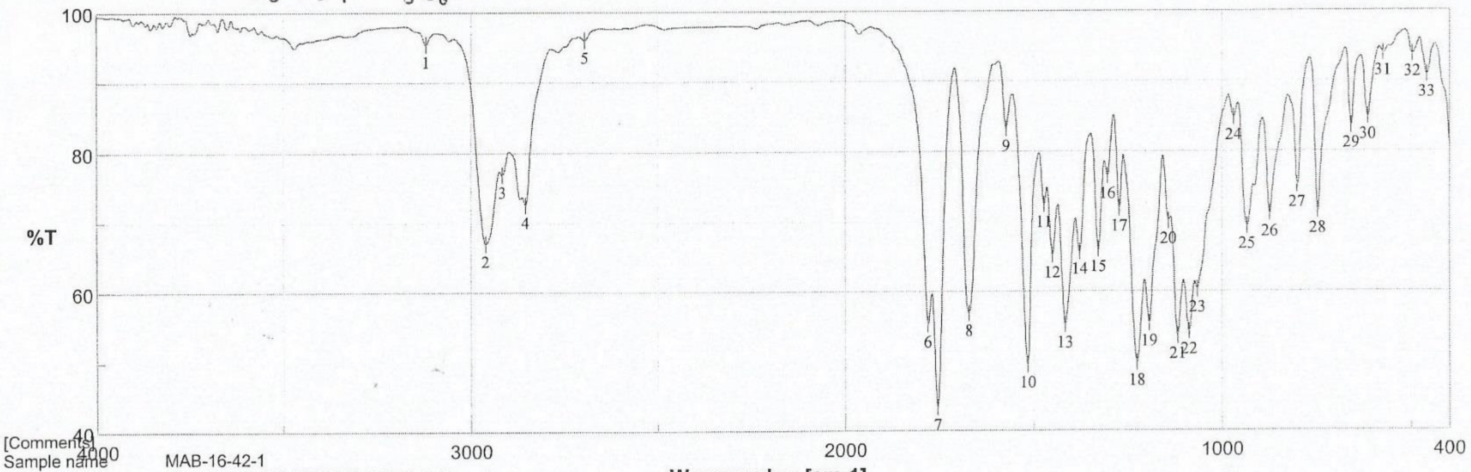

[Comment]
Sample name MAB-16-42-1
Comment PREPARED BY SHOBHA ON 21.7.16
User PROF. O. A. PHILIPS
Division RSPU GS01/05
Company Kuwait University

[Measurement Information]
Model Name FT/IR-6300typeA
Serial Number A009861024

Light Source Standard
Detector TGS
Accumulation Auto (38)
Resolution 4 cm-1
Zero Filling On
Apodization Cosine
Gain Auto (4)
Aperture Auto (7.1 mm)
Scanning Speed Auto (2 mm/sec)
Filter Auto (10000 Hz)

Result of Peak Picking

No.	Position	Intensity	No.	Position	Intensity	No.	Position	Intensity	No.	Position	Intensity
1	3119.3	95.3865	2	2960.2	66.9115	3	2915.84	76.8201	4	2853.17	72.5032
5	2696	95.9727	6	1779.01	55.2265	7	1753.94	43.3169	8	1671.02	56.8278
9	1571.7	83.2399	10	1514.81	49.4419	11	1470.46	72.373	12	1449.24	65.1423
13	1414.53	55.1509	14	1375.96	65.6364	15	1326.79	65.993	16	1302.68	76.4772
17	1270.86	71.8535	18	1224.58	49.7322	19	1192.76	55.3866	20	1140.69	69.9807
21	1116.58	53.554	22	1086.69	54.1634	23	1064.51	60.1814	24	968.09	84.7947
25	932.414	69.2845	26	873.596	70.9998	27	800.314	75.1892	28	746.317	71.433
29	658.571	83.6034	30	615.181	84.8542	31	574.683	93.8975	32	497.544	93.7699
33	459.939	90.8566									

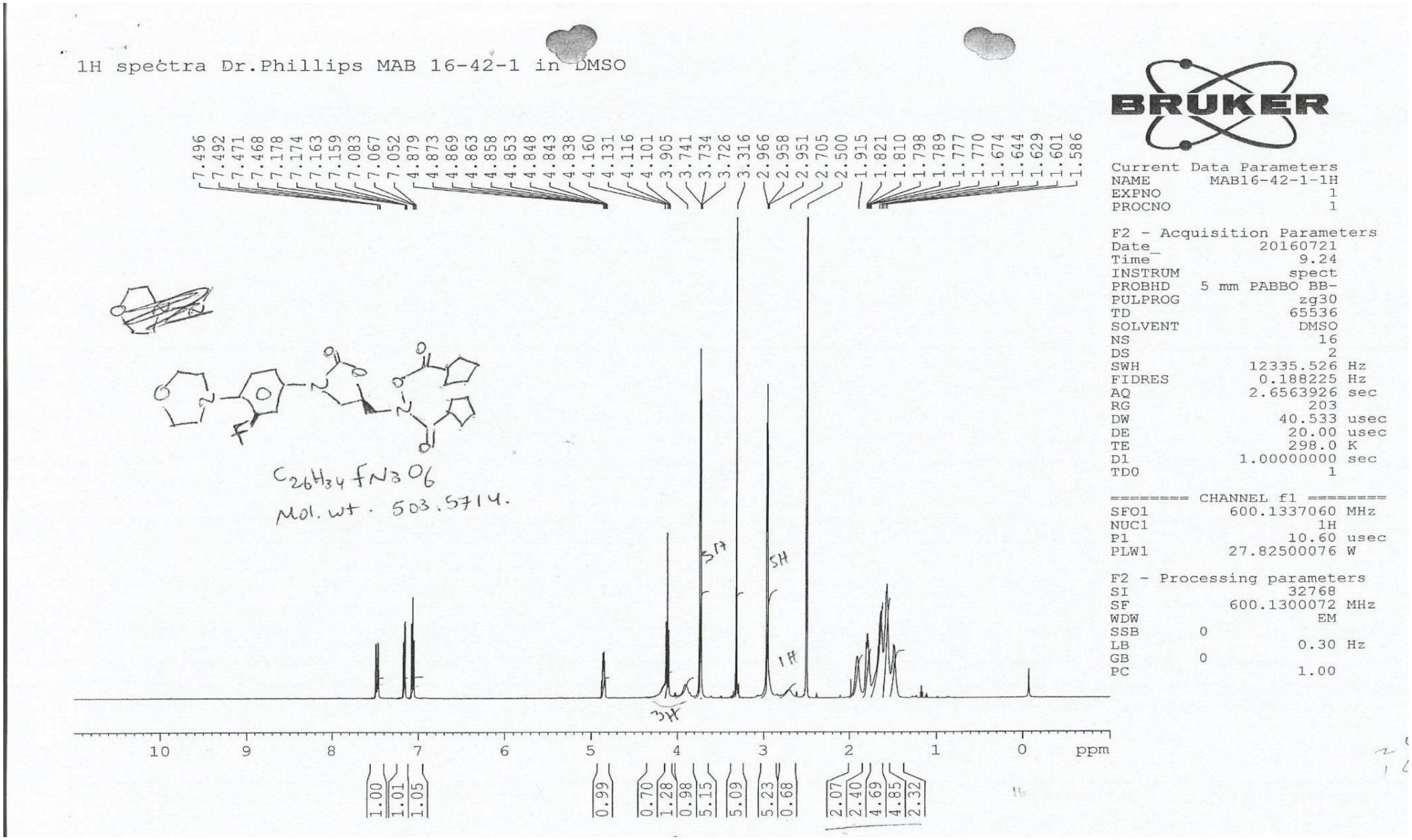
1H spectra Dr.Phillips MAB 16-42-1 in DMSO
BRUKER
7.496
7.492
7.471
7.468
7.178
7.174
7.163
7.159
7.083
7.067
7.052
4.879
4.873
4.869
4.863
4.858
4.853
4.848
4.843
4.838
4.160
4.131
4.116
4.101
3.905
3.741
3.734
3.726
3.316
2.966
2.958
2.951
2.705
2.500
1.915
1.821
1.810
1.798
1.789
1.777
1.770
1.674
1.644
1.629
1.601
1.586
Current Data Parameters
NAME MAB16-42-1-1H
EXPNO 1
PROCNO 1
F2 - Acquisition Parameters
Date_ 20160721
Time 9.24
INSTRUM spect
PROBHD 5 mm PABBO BB-
PULPROG zg30
TD 65536
SOLVENT DMSO
NS 16
DS 2
SWH 12335.526 Hz
FIDRES 0.188225 Hz
AQ 2.6563926 sec
RG 203
DW 40.533 usec
DE 20.00 usec
TE 298.0 K
D1 1.00000000 sec
TD0 1
======== CHANNEL f1 ========
SFO1 600.1337060 MHz
NUC1 1H
P1 10.60 usec
PLW1 27.82500076 W
F2 - Processing parameters
SI 32768
SF 600.1300072 MHz
WDW EM
SSB 0
LB 0.30 Hz
GB 0
PC 1.00
C26H34FN3O6
Mol. wt. 503.5714.
5H
5H
1H
3H
10
9
8
7
6
5
4
3
2
1
0
ppm
1.00
1.01
1.05
0.99
0.70
1.28
0.98
5.15
5.09
5.23
0.68
2.07
2.40
4.69
4.85
2.32

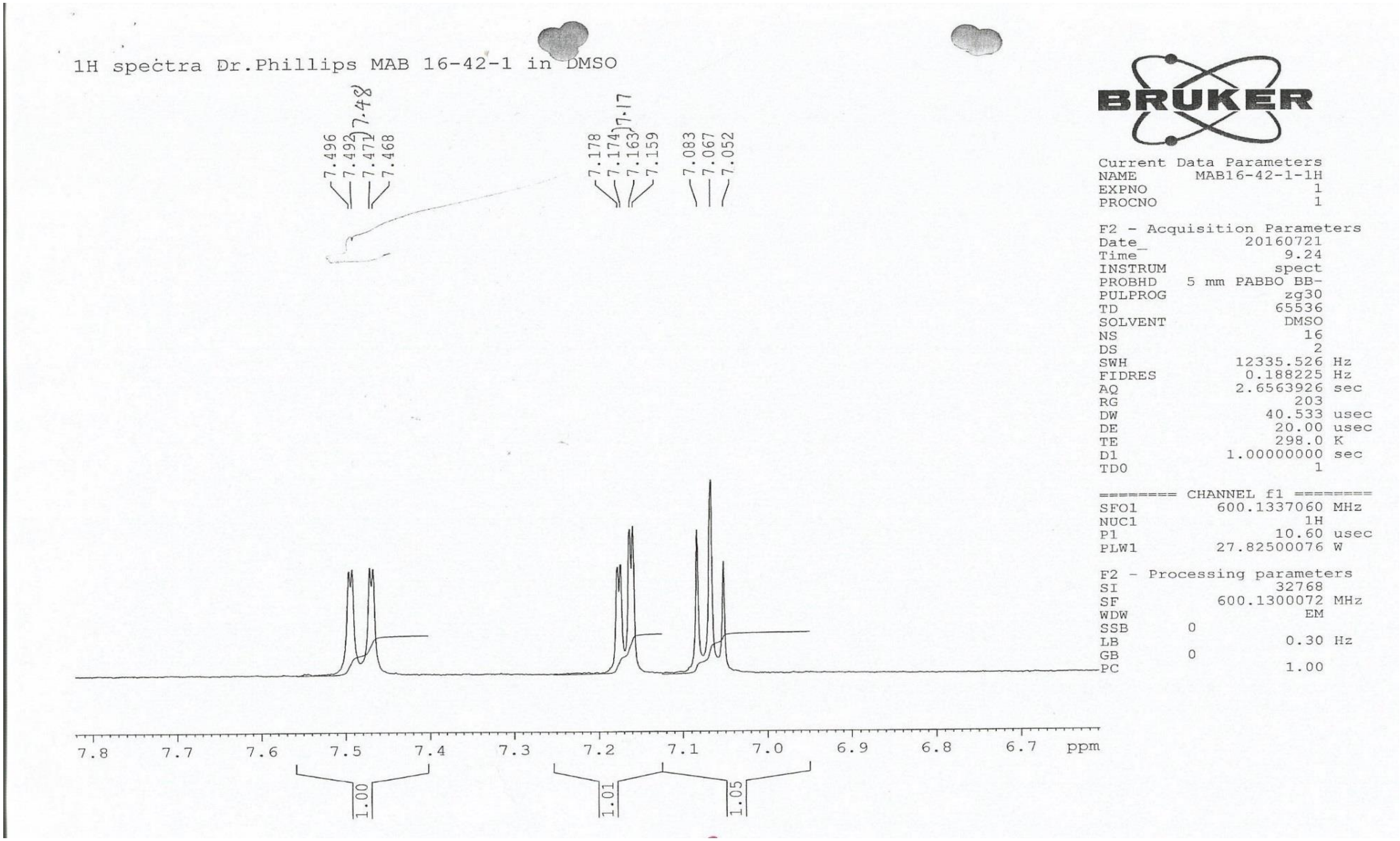
1H spectra Dr.Phillips MAB 16-42-1 in DMSO
7.496
7.492
7.471
7.468
7.48
7.178
7.174
7.163
7.159
7.17
7.083
7.067
7.052
BRUKER
Current Data Parameters
NAME MAB16-42-1-1H
EXPNO 1
PROCNO 1
F2 - Acquisition Parameters
Date_ 20160721
Time 9.24
INSTRUM spect
PROBHD 5 mm PABBO BB-
PULPROG zg30
TD 65536
SOLVENT DMSO
NS 16
DS 2
SWH 12335.526 Hz
FIDRES 0.188225 Hz
AQ 2.6563926 sec
RG 203
DW 40.533 usec
DE 20.00 usec
TE 298.0 K
D1 1.00000000 sec
TD0 1
======== CHANNEL f1 ========
SFO1 600.1337060 MHz
NUC1 1H
P1 10.60 usec
PLW1 27.82500076 W
F2 - Processing parameters
SI 32768
SF 600.1300072 MHz
WDW EM
SSB 0
LB 0.30 Hz
GB 0
PC 1.00
7.8 7.7 7.6 7.5 7.4 7.3 7.2 7.1 7.0 6.9 6.8 6.7 ppm
1.00
1.01
1.05

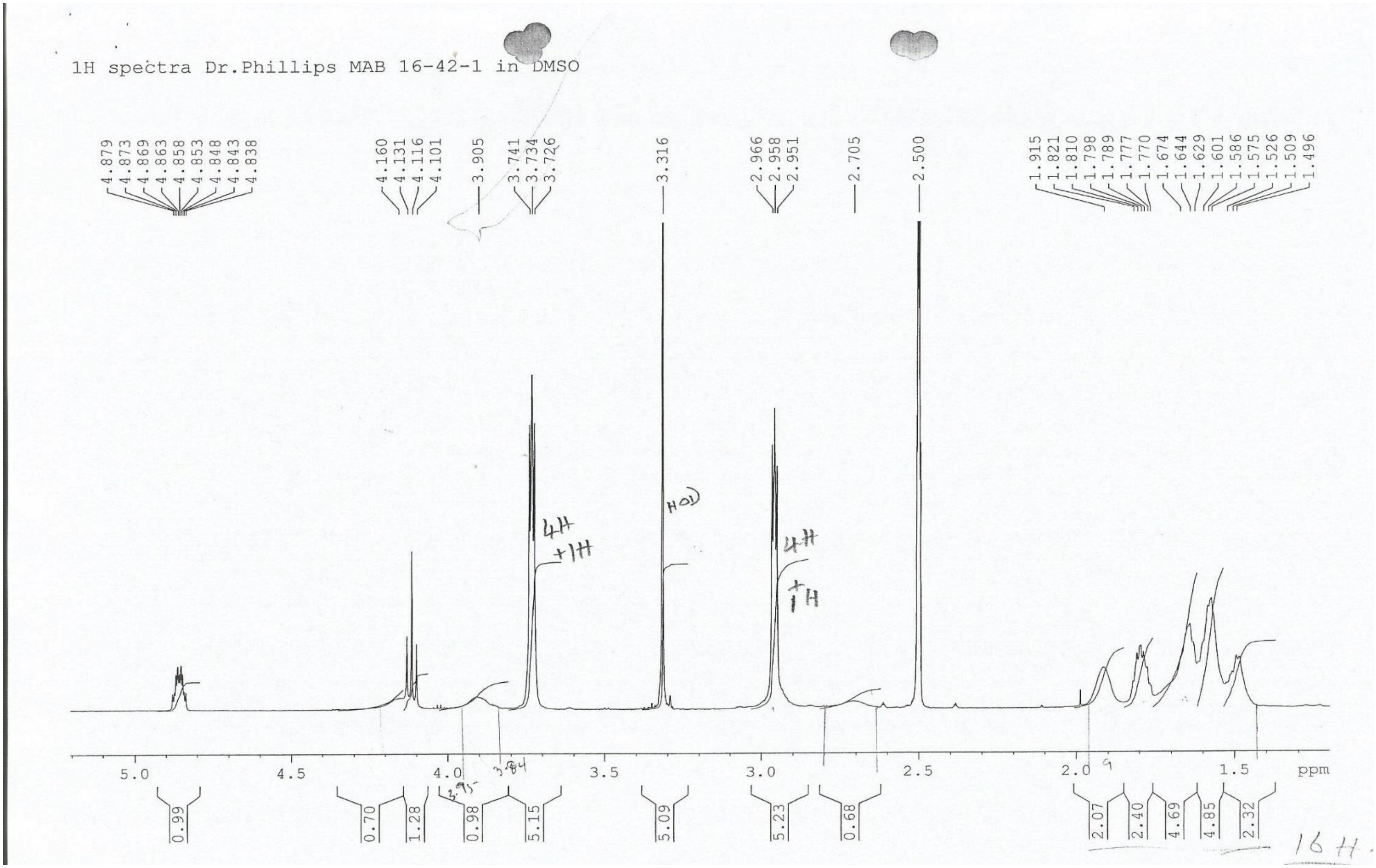

1H spectra Dr.Phillips MAB 16-42-1 in DMSO
4.879
4.873
4.869
4.863
4.858
4.853
4.848
4.843
4.838
4.160
4.131
4.116
4.101
3.905
3.741
3.734
3.726
3.316
2.966
2.958
2.951
2.705
2.500
1.915
1.821
1.810
1.798
1.789
1.777
1.770
1.674
1.644
1.629
1.601
1.586
1.575
1.526
1.509
1.496
5.0
4.5
4.0
3.5
3.0
2.5
2.0
1.5
ppm
0.99
0.70
1.28
0.98
5.15
5.09
5.23
0.68
2.07
2.40
4.69
4.85
2.32

MAB-16-58-1
$C_{20}H_{26}FN_3O_5$
Mol. wt = 407.4424 g/mol.

RESEARCH SECTOR PROJECTS UNIT
PROJECT – GS 02/10
Faculty of Science

INSTRUMENT NAME: LC- MS/MS (Xevo G2-S QToF)   INSTRUMENT PROJECT No: GS 02/10
ANALYST NAME: B GODWIN NOBLE CHANDAR   PH 244

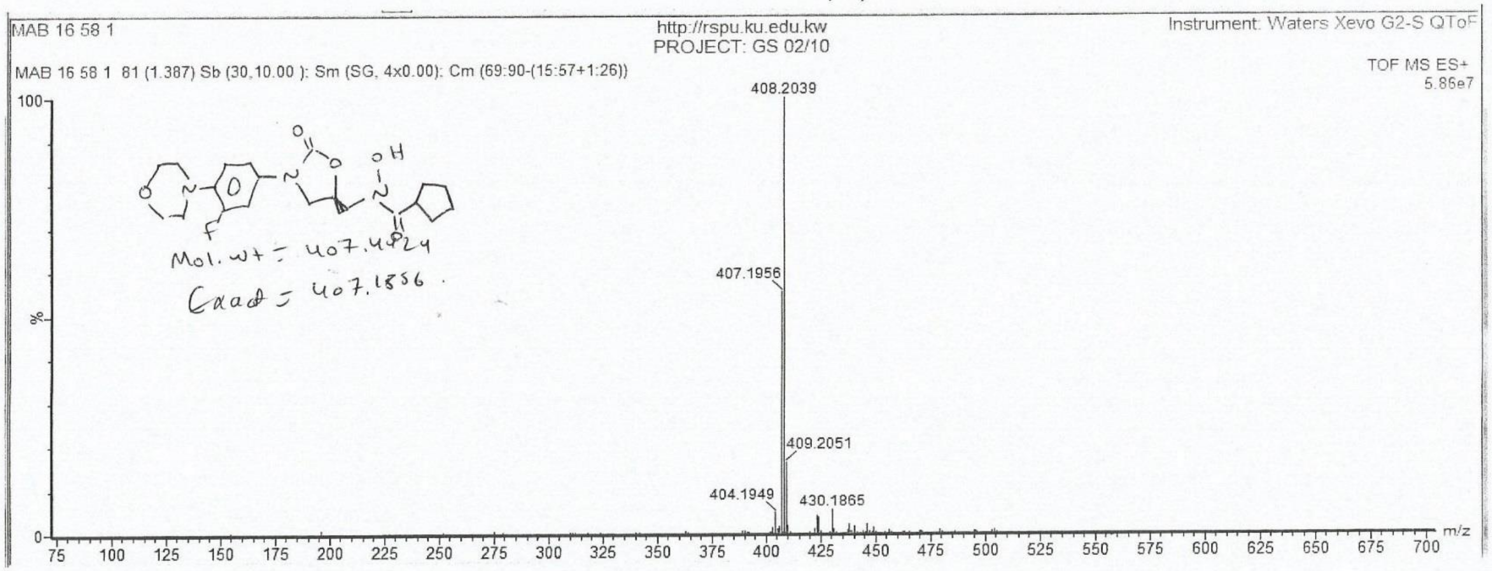

*Please acknowledge **RESEARCH SECTOR PROJECT NUMBER (GS 02/10) in PUBLICATIONS.***
* *Please visit our website* ***www.science.saf.kuniv.edu*** *for more information on RSPU facilities.*
Please collect your samples within one week after collecting the results.

Research Sector Projects Unit 01/05
Kuwait University

RSPU
Research Sector Projects Unit
KUWAIT UNIVERSITY

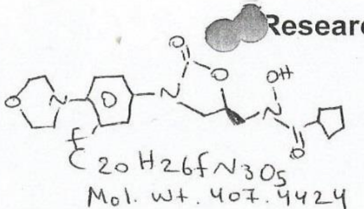

$C_{20}H_{26}FN_3O_5$
Mol. wt. 407.4424

FT- IR Spectral Data

PH244

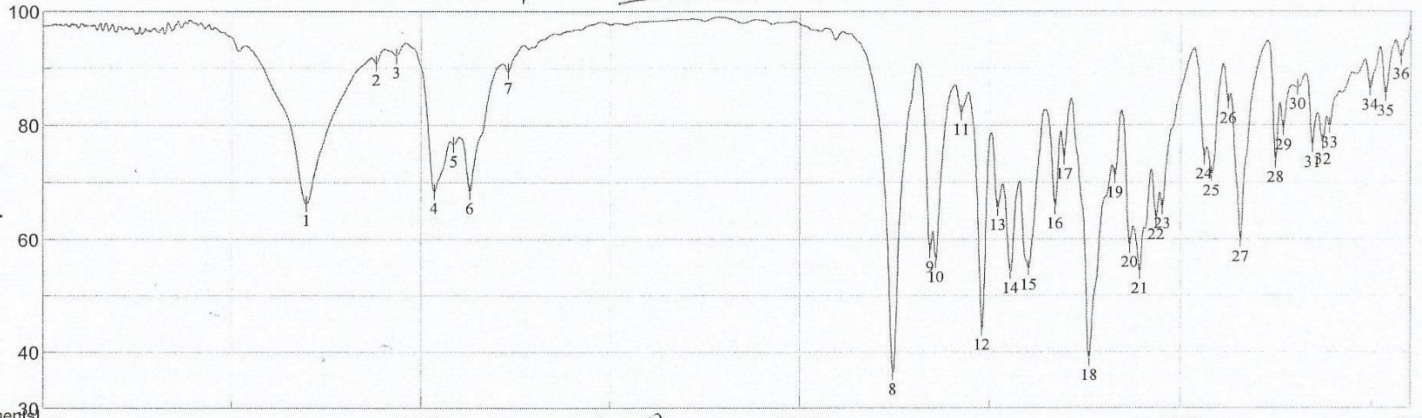

[Comments]

Sample name	MAB-16-58-1
Comment	PREPARED BY SHOBHA 0N 31.7.16
User	PROF. FAKHREIA
Division	RSPU GS01/05
Company	Kuwait University

[Measurement Information]

Model Name	FT/IR-6300typeA
Serial Number	A009861024
Light Source	Standard
Detector	TGS
Accumulation	Auto (31)
Resolution	4 cm-1
Zero Filling	On
Apodization	Cosine
Gain	Auto (2)
Aperture	Auto (7.1 mm)
Scanning Speed	Auto (2 mm/sec)
Filter	Auto (10000 Hz)

Result of Peak Picking

No.	Position	Intensity	No.	Position	Intensity	No.	Position	Intensity	No.	Position	Intensity
1	3301.54	66.1656	2	3118.33	90.7967	3	3065.3	92.1409	4	2964.05	68.2759
5	2912.95	76.5437	6	2869.56	68.3483	7	2769.28	89.4412	8	1752.98	36.3057
9	1655.59	57.9353	10	1640.16	56.1725	11	1574.59	82.1824	12	1519.63	44.1704
13	1478.17	65.4148	14	1444.42	54.1871	15	1397.17	54.8137	16	1327.75	65.7043
17	1304.61	74.4286	18	1239.04	38.7288	19	1171.54	71.1249	20	1132.97	58.812
21	1106.94	54.155	22	1063.55	63.752	23	1047.16	65.6562	24	938.199	74.4507
25	918.914	71.4743	26	875.524	84.1032	27	842.74	59.8415	28	752.102	73.8188
29	731.853	79.4768	30	694.248	86.5482	31	655.679	76.4855	32	628.68	77.0099
33	611.324	79.8532	34	506.223	86.4045	35	465.725	85.3807	36	426.191	91.8037

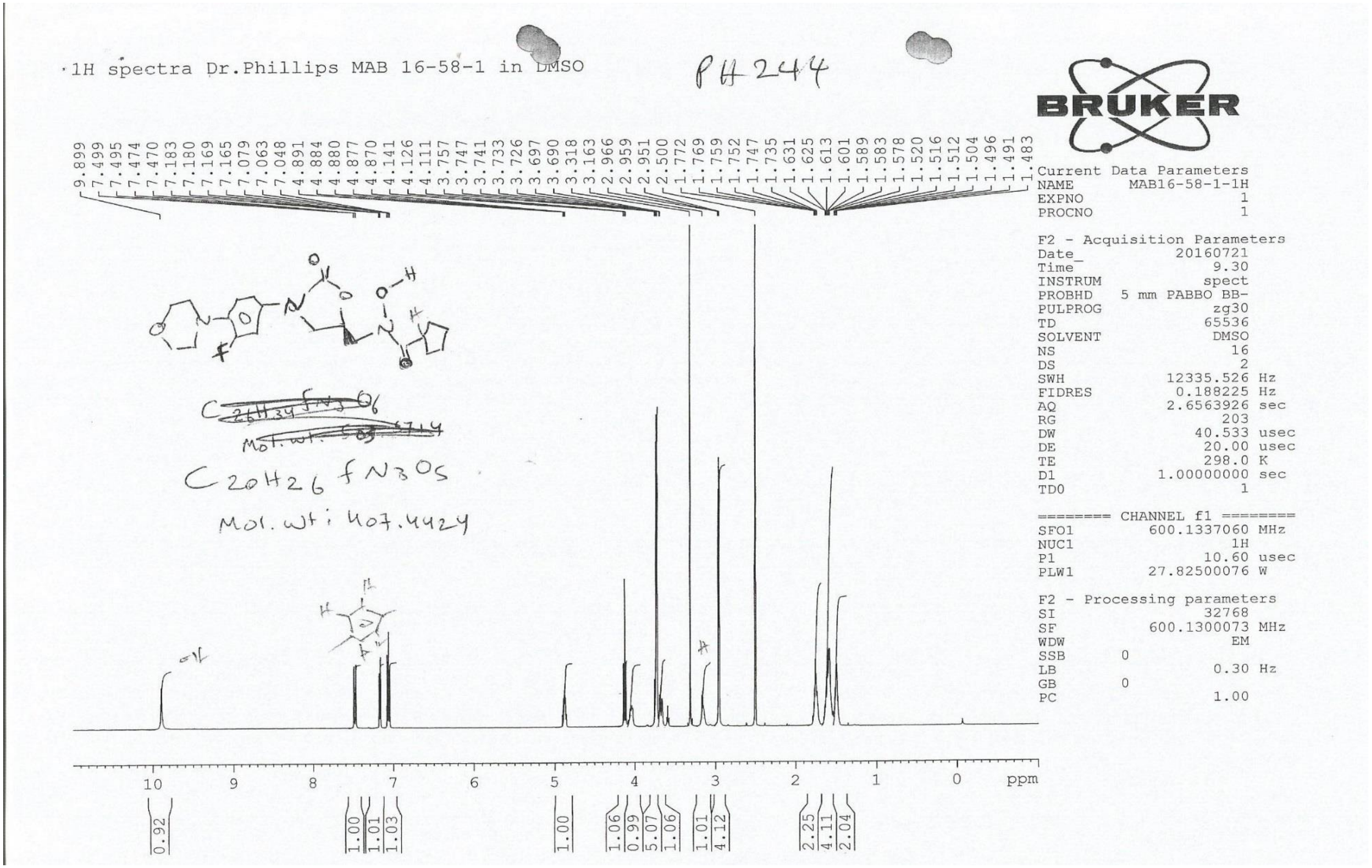
1H spectra Dr.Phillips MAB 16-58-1 in DMSO
PH244
BRUKER
9.899
7.499
7.495
7.474
7.470
7.183
7.180
7.169
7.165
7.079
7.063
7.048
4.891
4.884
4.880
4.877
4.870
4.141
4.126
4.111
3.757
3.747
3.741
3.733
3.726
3.697
3.690
3.318
3.163
2.966
2.959
2.951
2.500
1.772
1.769
1.759
1.752
1.747
1.735
1.631
1.625
1.613
1.601
1.589
1.583
1.578
1.520
1.516
1.512
1.504
1.496
1.491
1.483
C20H26FN3O5
Mol. wt: 407.4424
Current Data Parameters
NAME MAB16-58-1-1H
EXPNO 1
PROCNO 1
F2 - Acquisition Parameters
Date_ 20160721
Time 9.30
INSTRUM spect
PROBHD 5 mm PABBO BB-
PULPROG zg30
TD 65536
SOLVENT DMSO
NS 16
DS 2
SWH 12335.526 Hz
FIDRES 0.188225 Hz
AQ 2.6563926 sec
RG 203
DW 40.533 usec
DE 20.00 usec
TE 298.0 K
D1 1.00000000 sec
TD0 1
======== CHANNEL f1 ========
SFO1 600.1337060 MHz
NUC1 1H
P1 10.60 usec
PLW1 27.82500076 W
F2 - Processing parameters
SI 32768
SF 600.1300073 MHz
WDW EM
SSB 0
LB 0.30 Hz
GB 0
PC 1.00
10
9
8
7
6
5
4
3
2
1
0
ppm
0.92
1.00
1.01
1.03
1.00
1.06
0.99
5.07
1.06
1.01
4.12
2.25
4.11
2.04

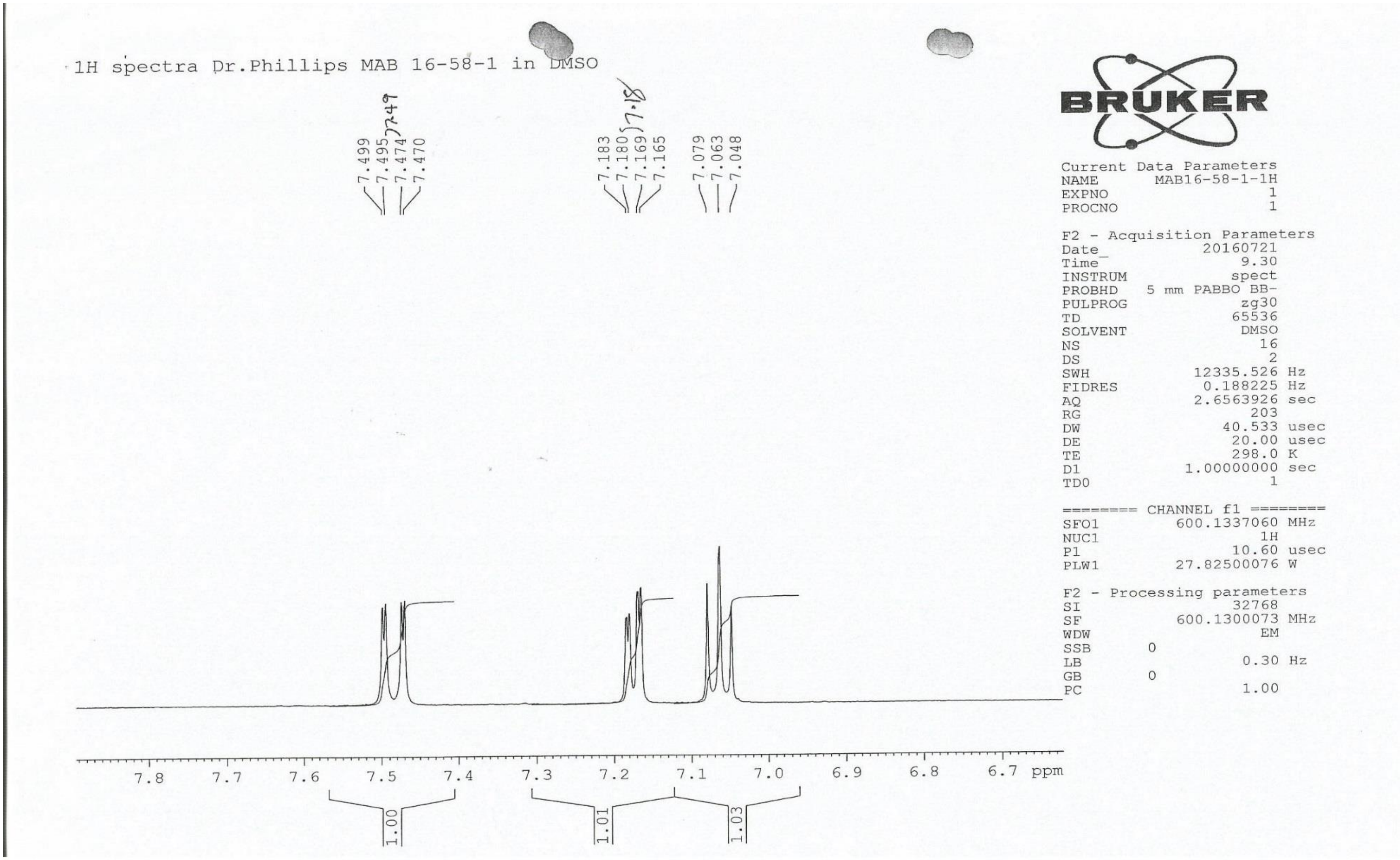
1H spectra Dr.Phillips MAB 16-58-1 in DMSO
7.499
7.495
7.474
7.470
7.183
7.180
7.169
7.165
7.079
7.063
7.048
BRUKER
Current Data Parameters
NAME MAB16-58-1-1H
EXPNO 1
PROCNO 1
F2 - Acquisition Parameters
Date_ 20160721
Time 9.30
INSTRUM spect
PROBHD 5 mm PABBO BB-
PULPROG zg30
TD 65536
SOLVENT DMSO
NS 16
DS 2
SWH 12335.526 Hz
FIDRES 0.188225 Hz
AQ 2.6563926 sec
RG 203
DW 40.533 usec
DE 20.00 usec
TE 298.0 K
D1 1.00000000 sec
TD0 1
======== CHANNEL f1 ========
SFO1 600.1337060 MHz
NUC1 1H
P1 10.60 usec
PLW1 27.82500076 W
F2 - Processing parameters
SI 32768
SF 600.1300073 MHz
WDW EM
SSB 0
LB 0.30 Hz
GB 0
PC 1.00
7.8 7.7 7.6 7.5 7.4 7.3 7.2 7.1 7.0 6.9 6.8 6.7 ppm
1.00
1.01
1.03

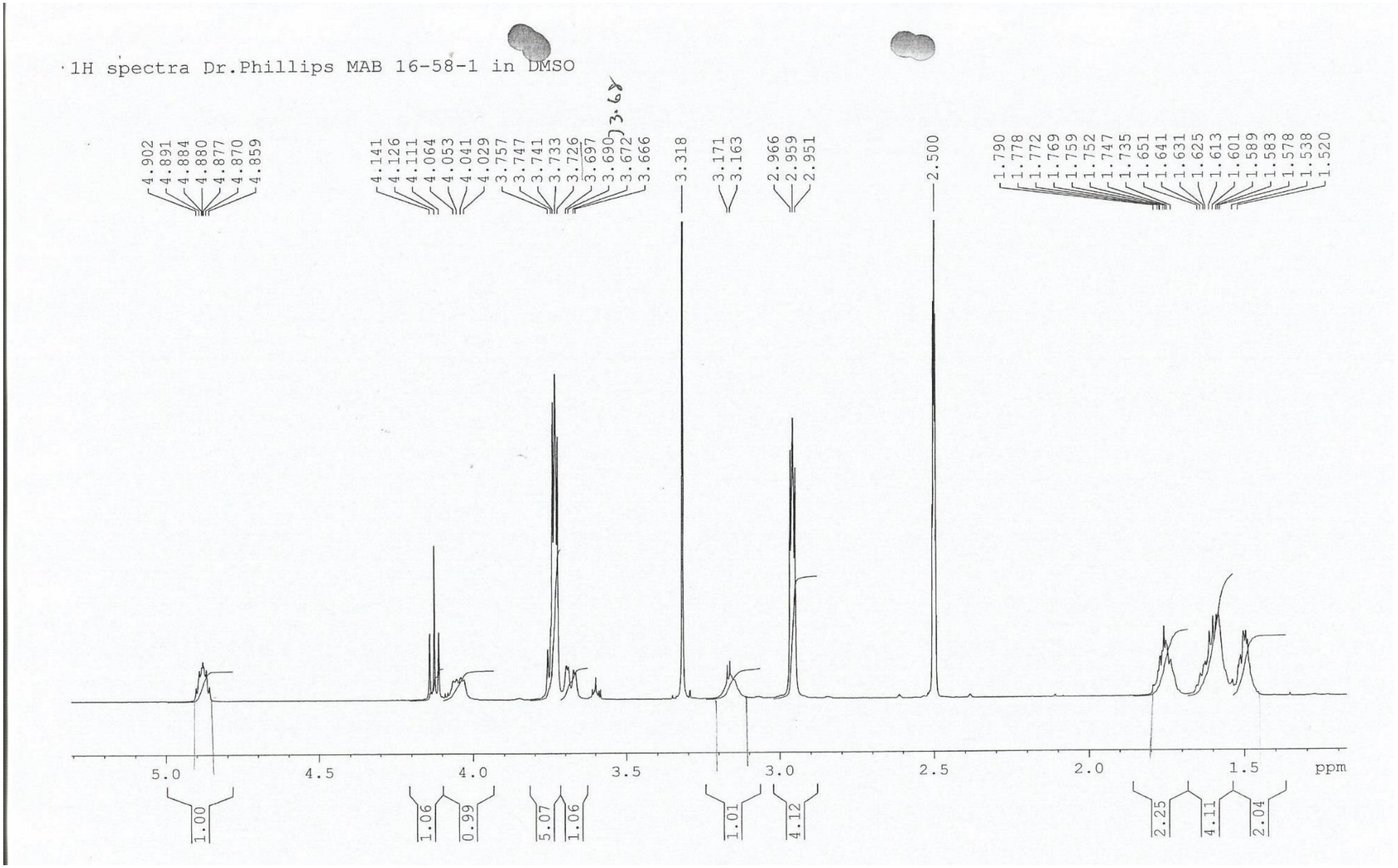
1H spectra Dr.Phillips MAB 16-58-1 in DMSO
3.68
4.902
4.891
4.884
4.880
4.877
4.870
4.859
4.141
4.126
4.111
4.064
4.053
4.041
4.029
3.757
3.747
3.741
3.733
3.726
3.697
3.690
3.672
3.666
3.318
3.171
3.163
2.966
2.959
2.951
2.500
1.790
1.778
1.772
1.769
1.759
1.752
1.747
1.735
1.651
1.641
1.631
1.625
1.613
1.601
1.589
1.583
1.578
1.538
1.520
5.0
4.5
4.0
3.5
3.0
2.5
2.0
1.5
ppm
1.00
1.06
0.99
5.07
1.06
1.01
4.12
2.25
4.11
2.04

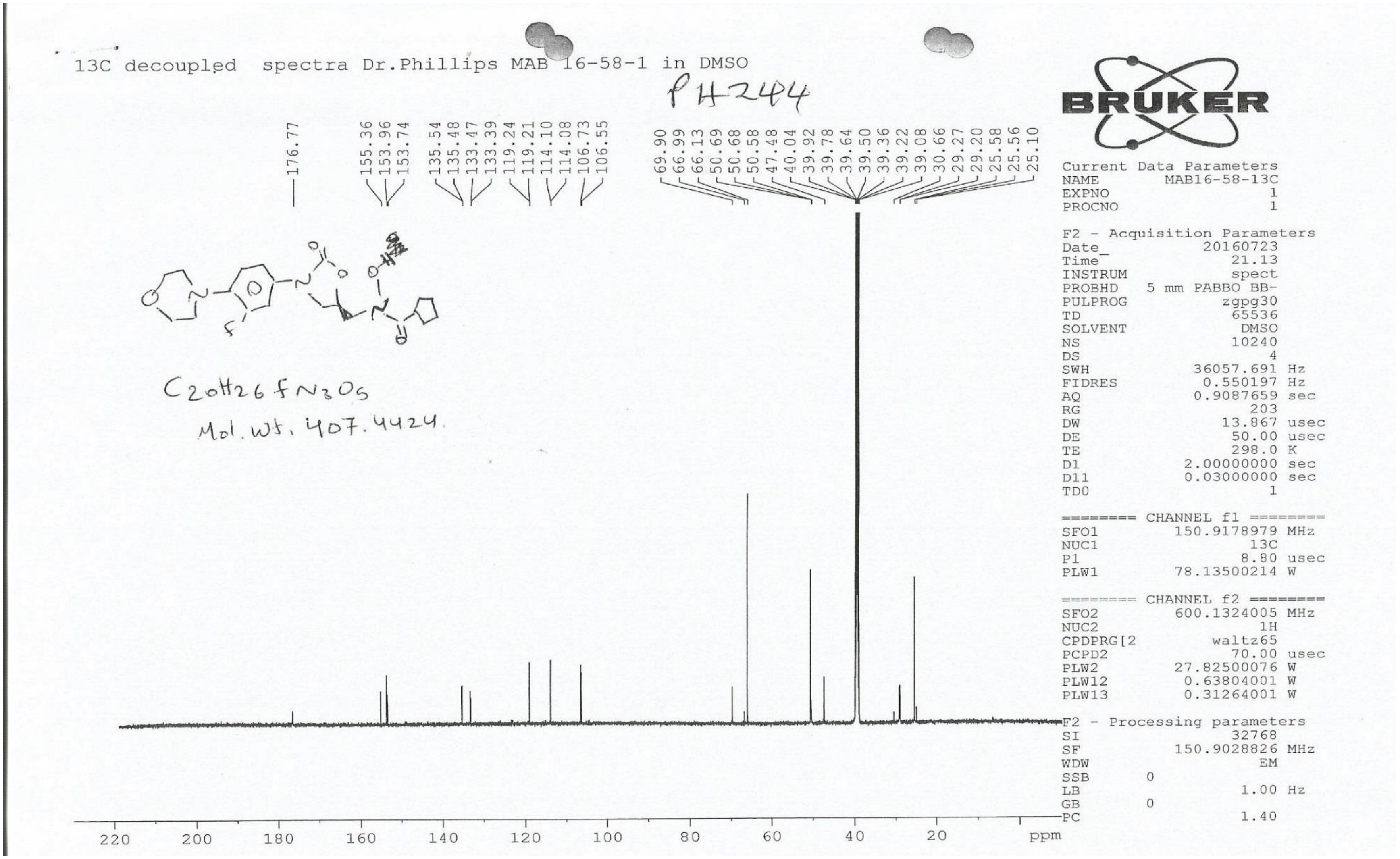
13C decoupled spectra Dr.Phillips MAB 16-58-1 in DMSO
PH244
176.77
155.36
153.96
153.74
135.54
135.48
133.47
133.39
119.24
119.21
114.10
114.08
106.73
106.55
69.90
66.99
66.13
50.69
50.68
50.58
47.48
40.04
39.92
39.78
39.64
39.50
39.36
39.22
39.08
30.66
29.27
29.20
25.58
25.56
25.10
C20H26FN3O5
Mol. Wt. 407.4424
BRUKER
Current Data Parameters
NAME MAB16-58-13C
EXPNO 1
PROCNO 1
F2 - Acquisition Parameters
Date_ 20160723
Time 21.13
INSTRUM spect
PROBHD 5 mm PABBO BB-
PULPROG zgpg30
TD 65536
SOLVENT DMSO
NS 10240
DS 4
SWH 36057.691 Hz
FIDRES 0.550197 Hz
AQ 0.9087659 sec
RG 203
DW 13.867 usec
DE 50.00 usec
TE 298.0 K
D1 2.00000000 sec
D11 0.03000000 sec
TD0 1
======== CHANNEL f1 ========
SFO1 150.9178979 MHz
NUC1 13C
P1 8.80 usec
PLW1 78.13500214 W
======== CHANNEL f2 ========
SFO2 600.1324005 MHz
NUC2 1H
CPDPRG[2 waltz65
PCPD2 70.00 usec
PLW2 27.82500076 W
PLW12 0.63804001 W
PLW13 0.31264001 W
F2 - Processing parameters
SI 32768
SF 150.9028826 MHz
WDW EM
SSB 0
LB 1.00 Hz
GB 0
PC 1.40
220 200 180 160 140 120 100 80 60 40 20 ppm

RESEARCH SECTOR PROJECTS UNIT
PROJECT – GS 02/10
Faculty of Science

INSTRUMENT NAME: LC- MS/MS (Xevo G2-S QToF) INSTRUMENT PROJECT No: GS 02/10

ANALYST NAME: B GODWIN NOBLE CHANDAR

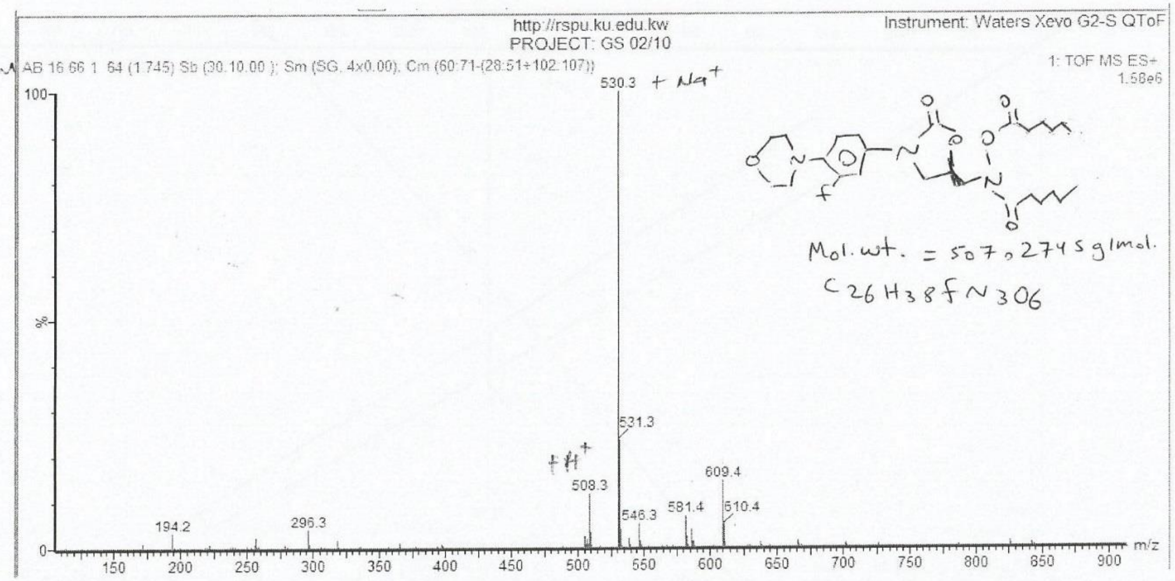

PH246

Please acknowledge RESEARCH SECTOR PROJECT NUMBER (GS 02/10) in PUBLICATIONS.

** Please visit our website* ***www.science.saf.kuniv.edu*** *for more information on RSPU facilities.*

Please collect your samples within one week after collecting the results.

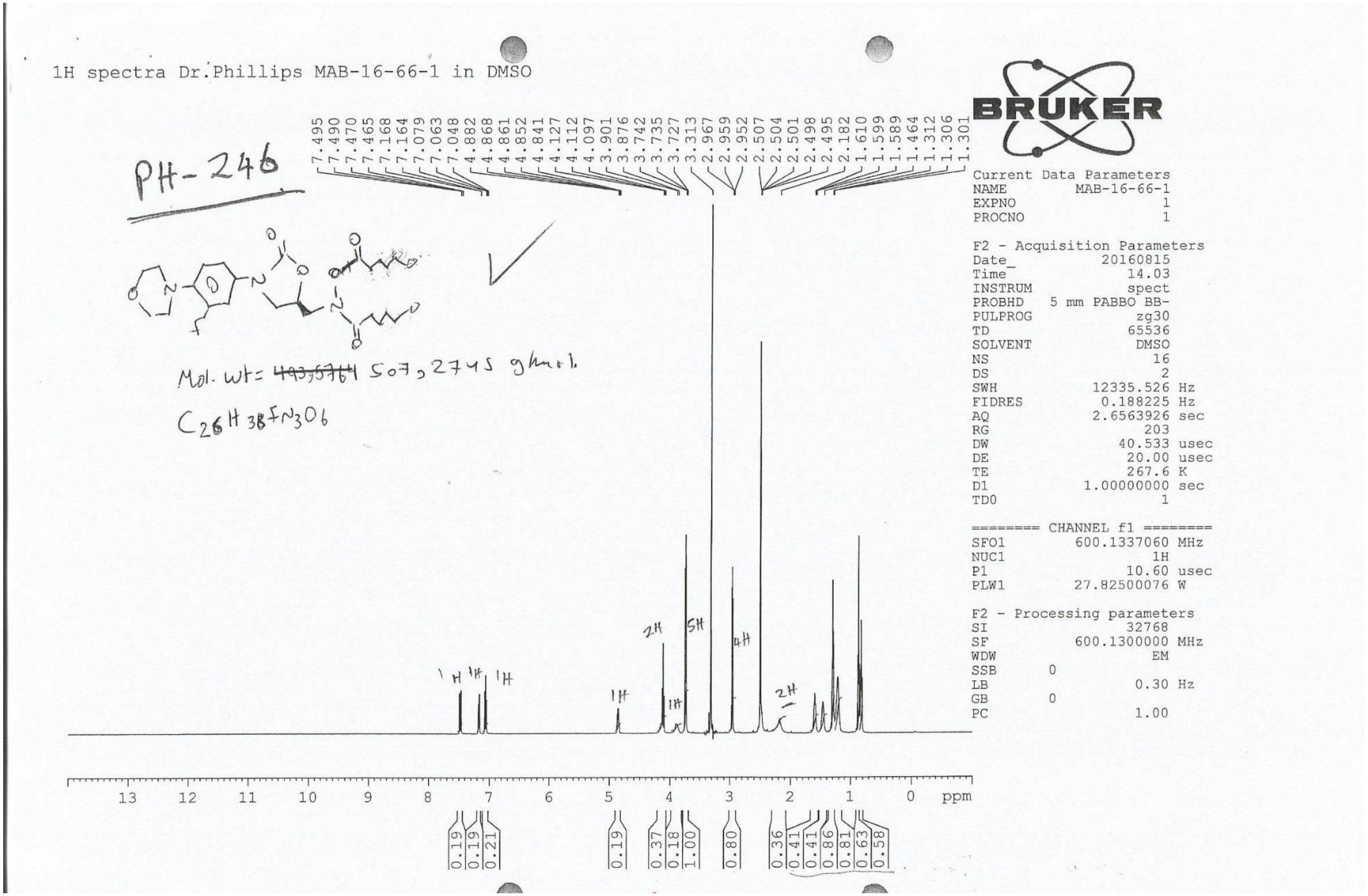
1H spectra Dr.Phillips MAB-16-66-1 in DMSO
PH-246
Mol. wt= 507,2745 g/mol
C26H38FN3O6
7.495 7.490 7.470 7.465 7.168 7.164 7.079 7.063 7.048 4.882 4.868 4.861 4.852 4.841 4.127 4.112 4.097 3.901 3.876 3.742 3.735 3.727 3.313 2.967 2.959 2.952 2.507 2.504 2.501 2.498 2.495 2.182 1.610 1.599 1.589 1.464 1.312 1.306 1.301
BRUKER
Current Data Parameters
NAME MAB-16-66-1
EXPNO 1
PROCNO 1
F2 - Acquisition Parameters
Date_ 20160815
Time 14.03
INSTRUM spect
PROBHD 5 mm PABBO BB-
PULPROG zg30
TD 65536
SOLVENT DMSO
NS 16
DS 2
SWH 12335.526 Hz
FIDRES 0.188225 Hz
AQ 2.6563926 sec
RG 203
DW 40.533 usec
DE 20.00 usec
TE 267.6 K
D1 1.00000000 sec
TD0 1
======== CHANNEL f1 ========
SFO1 600.1337060 MHz
NUC1 1H
P1 10.60 usec
PLW1 27.82500076 W
F2 - Processing parameters
SI 32768
SF 600.1300000 MHz
WDW EM
SSB 0
LB 0.30 Hz
GB 0
PC 1.00
13 12 11 10 9 8 7 6 5 4 3 2 1 0 ppm
0.19 0.19 0.21 0.19 0.37 0.18 1.00 0.80 0.36 0.41 0.41 0.86 0.81 0.63 0.58

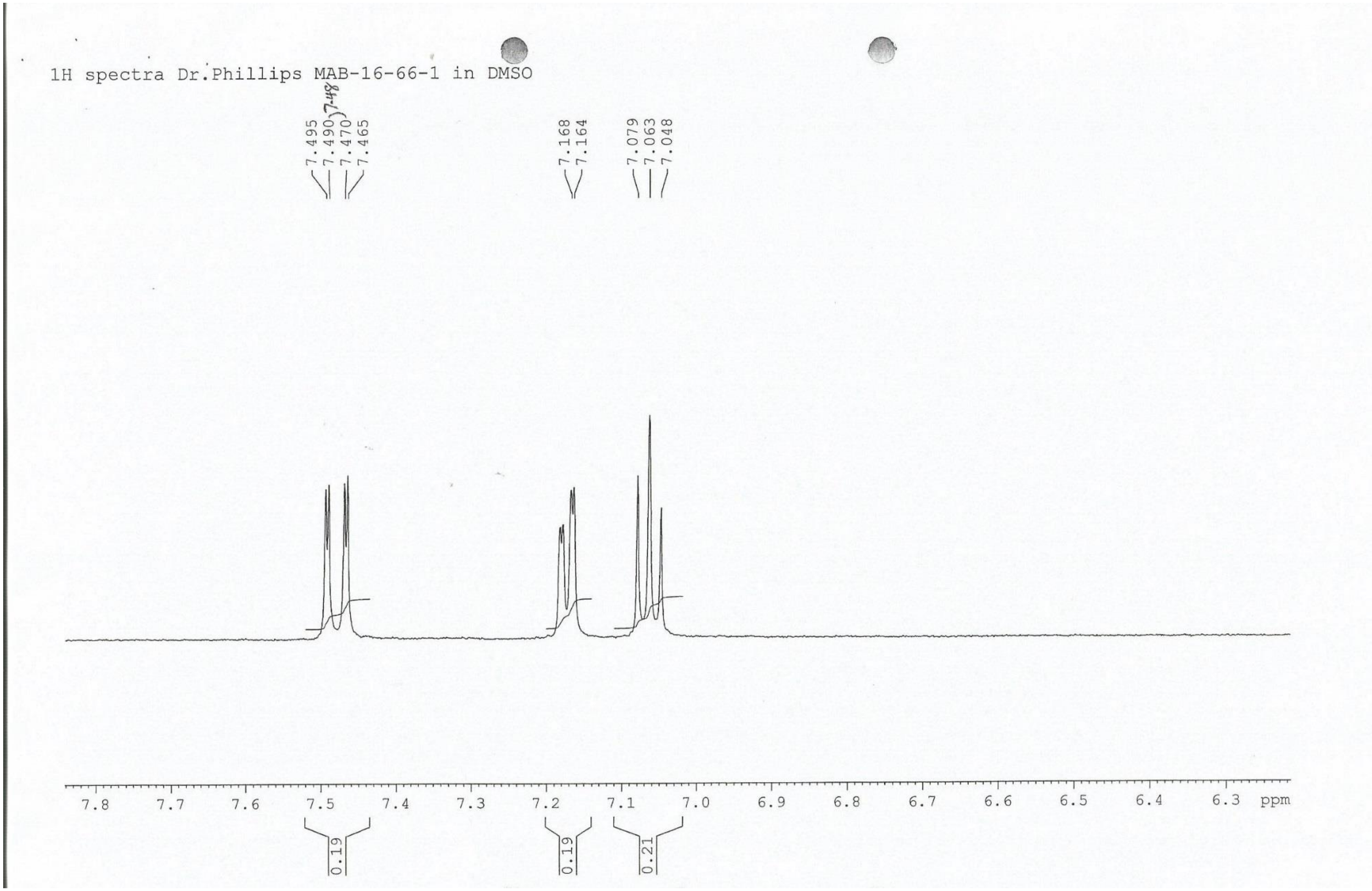
1H spectra Dr.Phillips MAB-16-66-1 in DMSO
7.495
7.490
7.470
7.465
7.48
7.168
7.164
7.079
7.063
7.048
7.8 7.7 7.6 7.5 7.4 7.3 7.2 7.1 7.0 6.9 6.8 6.7 6.6 6.5 6.4 6.3 ppm
0.19
0.19
0.21

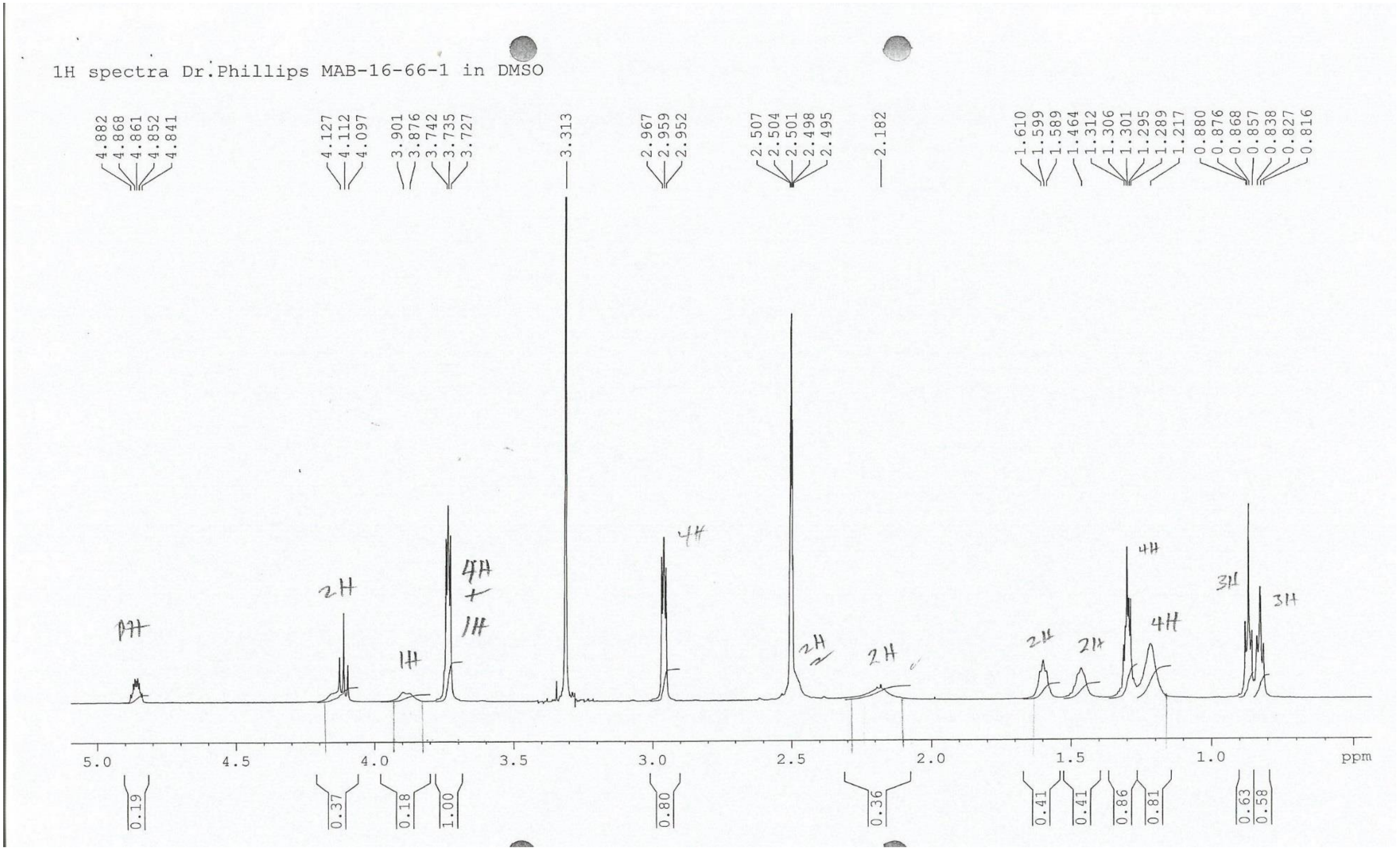
1H spectra Dr.Phillips MAB-16-66-1 in DMSO
4.882
4.868
4.861
4.852
4.841
4.127
4.112
4.097
3.901
3.876
3.742
3.735
3.727
3.313
2.967
2.959
2.952
2.507
2.504
2.501
2.498
2.495
2.182
1.610
1.599
1.589
1.464
1.312
1.306
1.301
1.295
1.289
1.217
0.880
0.876
0.868
0.857
0.838
0.827
0.816
ppm
0.19
0.37
0.18
1.00
0.80
0.36
0.41
0.41
0.86
0.81
0.63
0.58

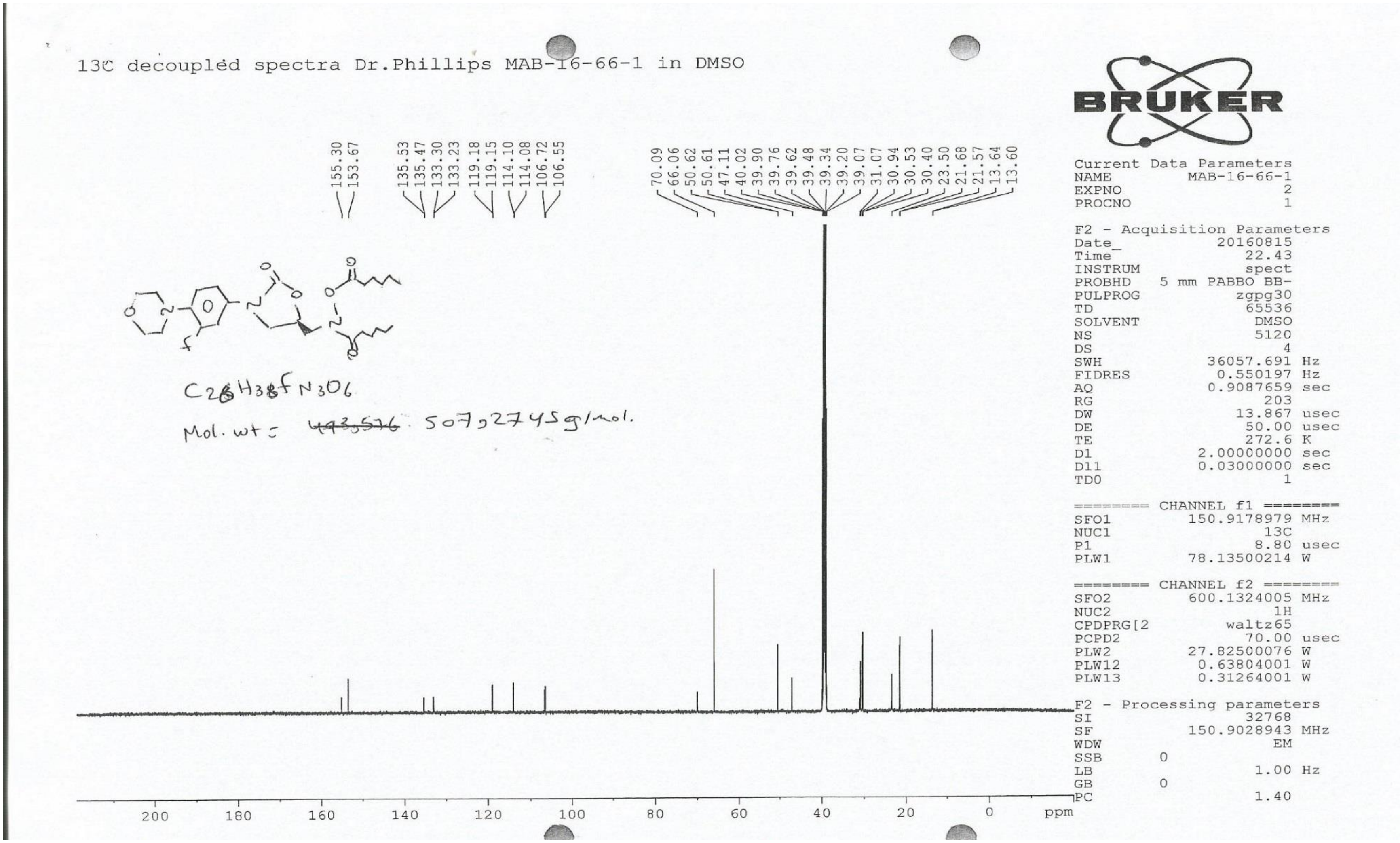
13C decoupled spectra Dr.Phillips MAB-16-66-1 in DMSO
155.30
153.67
135.53
135.47
133.30
133.23
119.18
119.15
114.10
114.08
106.72
106.55
70.09
66.06
50.62
50.61
47.11
40.02
39.90
39.76
39.62
39.48
39.34
39.20
39.07
31.07
30.94
30.53
30.40
23.50
21.68
21.57
13.64
13.60
BRUKER
Current Data Parameters
NAME MAB-16-66-1
EXPNO 2
PROCNO 1
F2 - Acquisition Parameters
Date_ 20160815
Time 22.43
INSTRUM spect
PROBHD 5 mm PABBO BB-
PULPROG zgpg30
TD 65536
SOLVENT DMSO
NS 5120
DS 4
SWH 36057.691 Hz
FIDRES 0.550197 Hz
AQ 0.9087659 sec
RG 203
DW 13.867 usec
DE 50.00 usec
TE 272.6 K
D1 2.00000000 sec
D11 0.03000000 sec
TD0 1
======== CHANNEL f1 ========
SFO1 150.9178979 MHz
NUC1 13C
P1 8.80 usec
PLW1 78.13500214 W
======== CHANNEL f2 ========
SFO2 600.1324005 MHz
NUC2 1H
CPDPRG[2 waltz65
PCPD2 70.00 usec
PLW2 27.82500076 W
PLW12 0.63804001 W
PLW13 0.31264001 W
F2 - Processing parameters
SI 32768
SF 150.9028943 MHz
WDW EM
SSB 0
LB 1.00 Hz
GB 0
PC 1.40
200 180 160 140 120 100 80 60 40 20 0 ppm

MAB-16-77 I

Mol. wt. = 409,4584 g/mol.

RESEARCH SECTOR PROJECTS UNIT
PROJECT – GS 02/10
Faculty of Science

INSTRUMENT NAME: LC- MS/MS (Xevo G2-S QToF) INSTRUMENT PROJECT No: GS 02/10

ANALYST NAME: B GODWIN NOBLE CHANDAR

PH 247

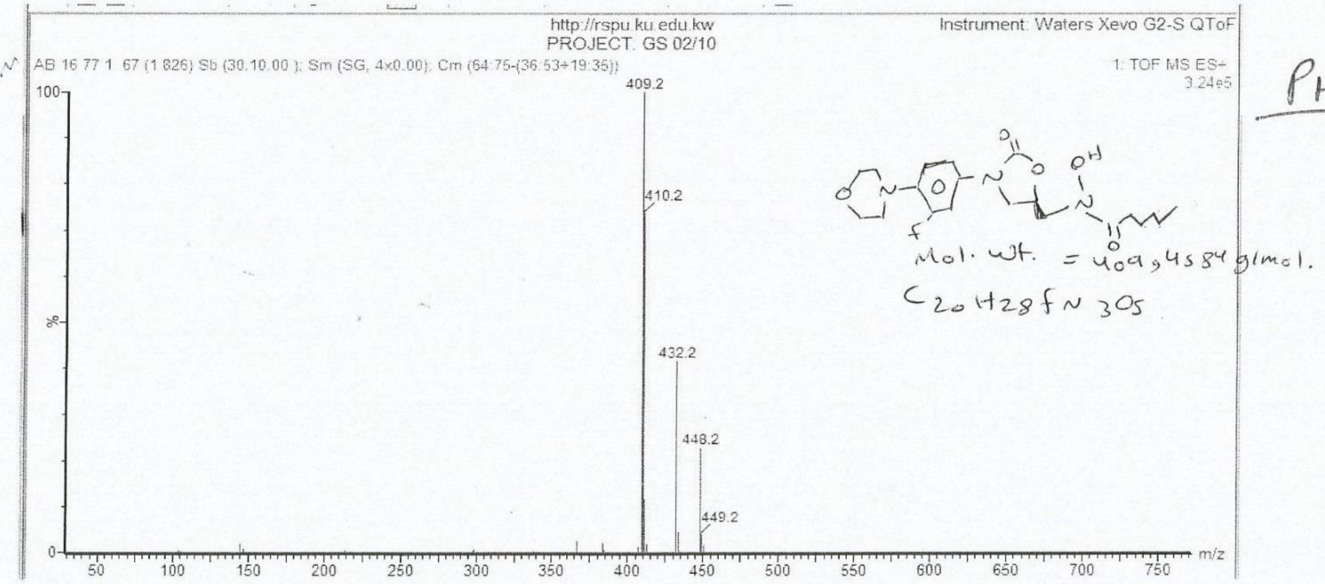

***Please acknowledge** RESEARCH SECTOR PROJECT **NUMBER (GS 02/10) in PUBLICATIONS.**

** Please visit our website* ***www.science.saf.kuniv.edu*** *for more information on RSPU facilities.*

****Please collect your samples within one week after collecting the results.***

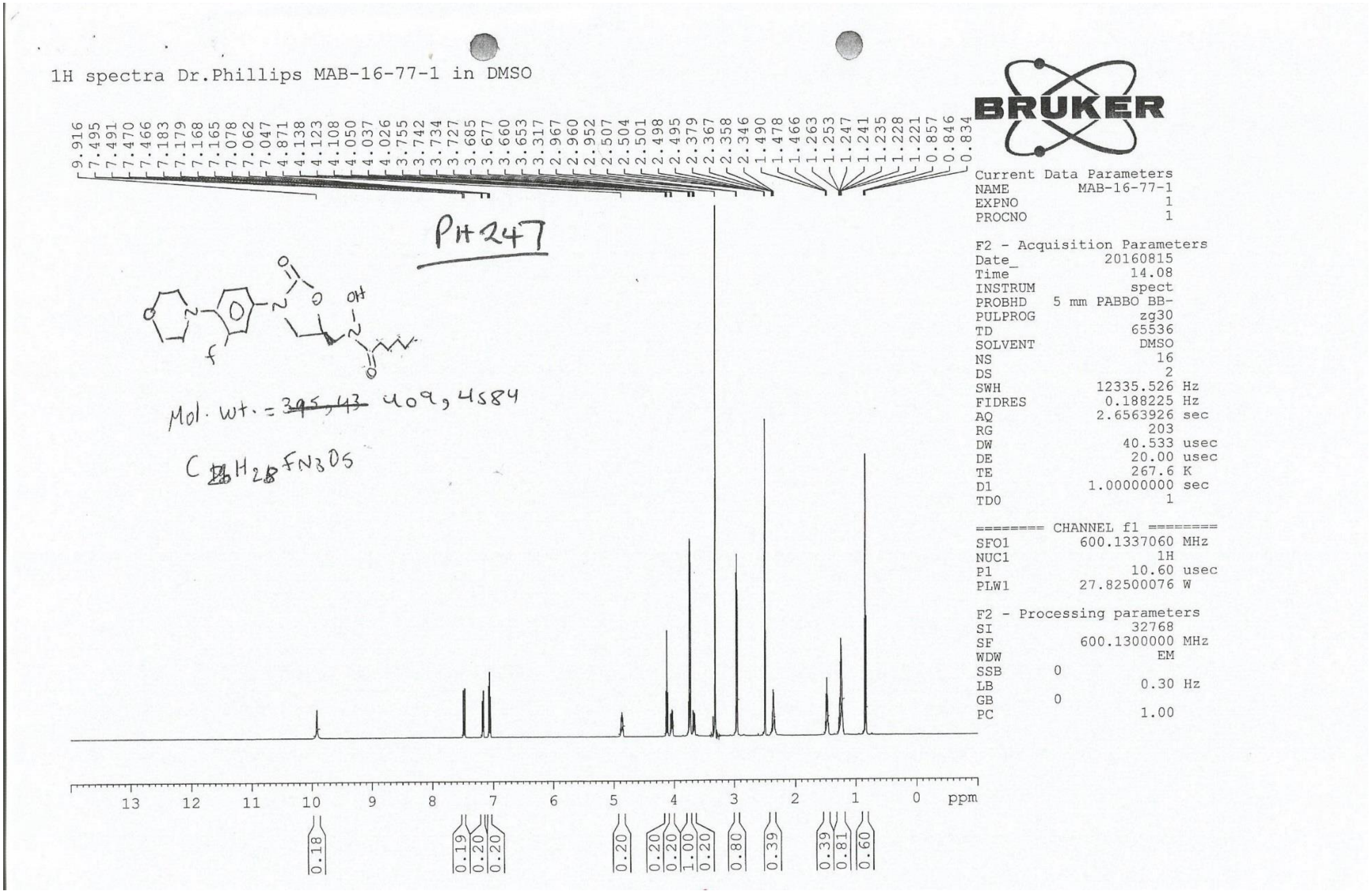
1H spectra Dr.Phillips MAB-16-77-1 in DMSO
9.916 7.495 7.491 7.470 7.466 7.183 7.179 7.168 7.165 7.078 7.062 7.047 4.871 4.138 4.123 4.108 4.050 4.037 4.026 3.755 3.742 3.734 3.727 3.685 3.677 3.660 3.653 3.317 2.967 2.960 2.952 2.507 2.504 2.501 2.498 2.495 2.379 2.367 2.358 2.346 1.490 1.478 1.466 1.263 1.253 1.247 1.241 1.235 1.228 1.221 0.857 0.846 0.834
BRUKER
PH247
Mol. wt. = 395,43 409,4584
C H28 FN3O5
13 12 11 10 9 8 7 6 5 4 3 2 1 0 ppm
0.18 0.19 0.20 0.20 0.20 0.20 0.20 1.00 0.20 0.80 0.39 0.39 0.81 0.60
Current Data Parameters
NAME MAB-16-77-1
EXPNO 1
PROCNO 1
F2 - Acquisition Parameters
Date_ 20160815
Time 14.08
INSTRUM spect
PROBHD 5 mm PABBO BB-
PULPROG zg30
TD 65536
SOLVENT DMSO
NS 16
DS 2
SWH 12335.526 Hz
FIDRES 0.188225 Hz
AQ 2.6563926 sec
RG 203
DW 40.533 usec
DE 20.00 usec
TE 267.6 K
D1 1.00000000 sec
TD0 1
======== CHANNEL f1 ========
SFO1 600.1337060 MHz
NUC1 1H
P1 10.60 usec
PLW1 27.82500076 W
F2 - Processing parameters
SI 32768
SF 600.1300000 MHz
WDW EM
SSB 0
LB 0.30 Hz
GB 0
PC 1.00

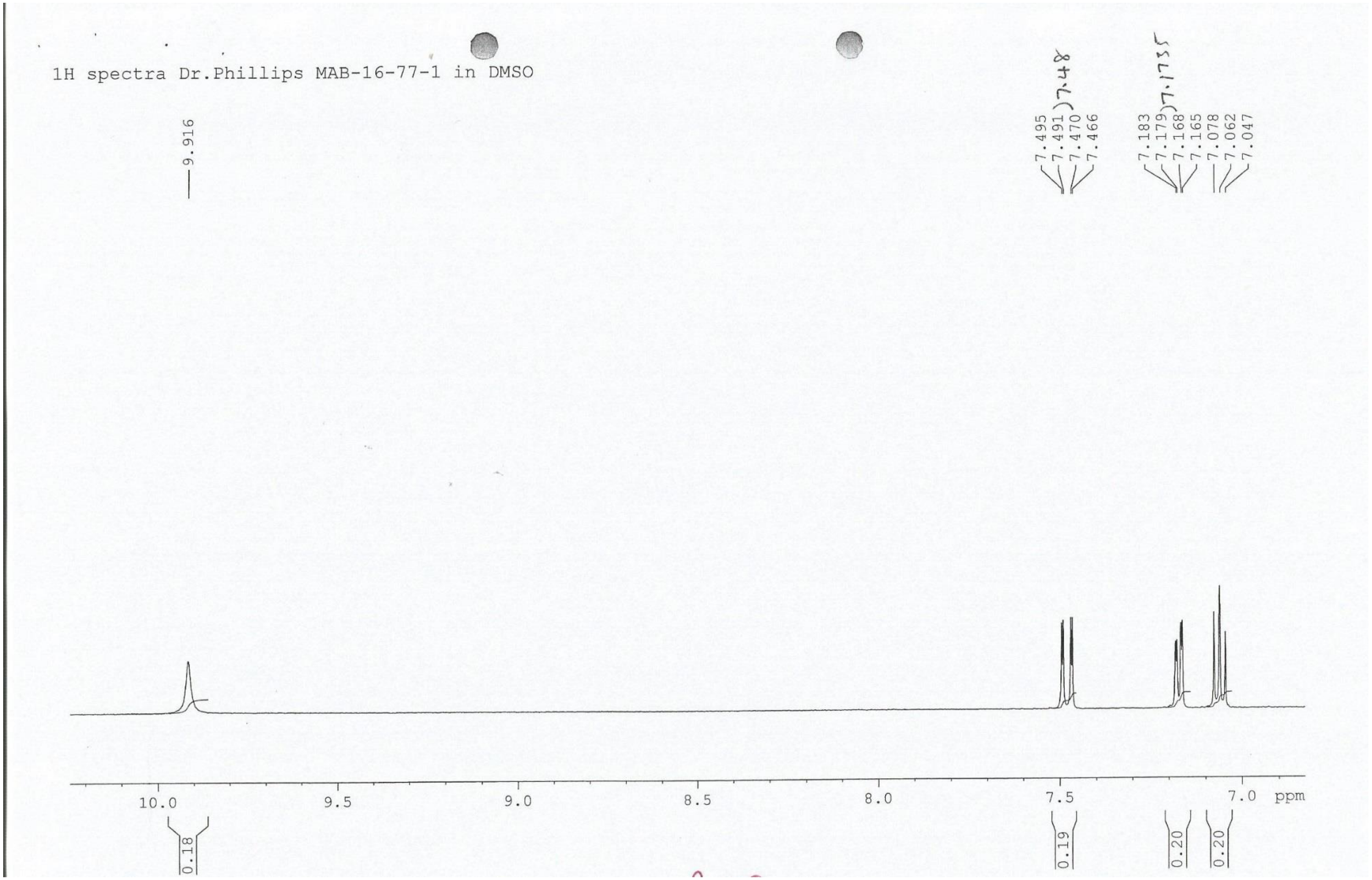
1H spectra Dr.Phillips MAB-16-77-1 in DMSO
9.916
7.495
7.491
7.470
7.466
7.48
7.183
7.179
7.168
7.165
7.1735
7.078
7.062
7.047
10.0
9.5
9.0
8.5
8.0
7.5
7.0
ppm
0.18
0.19
0.20
0.20

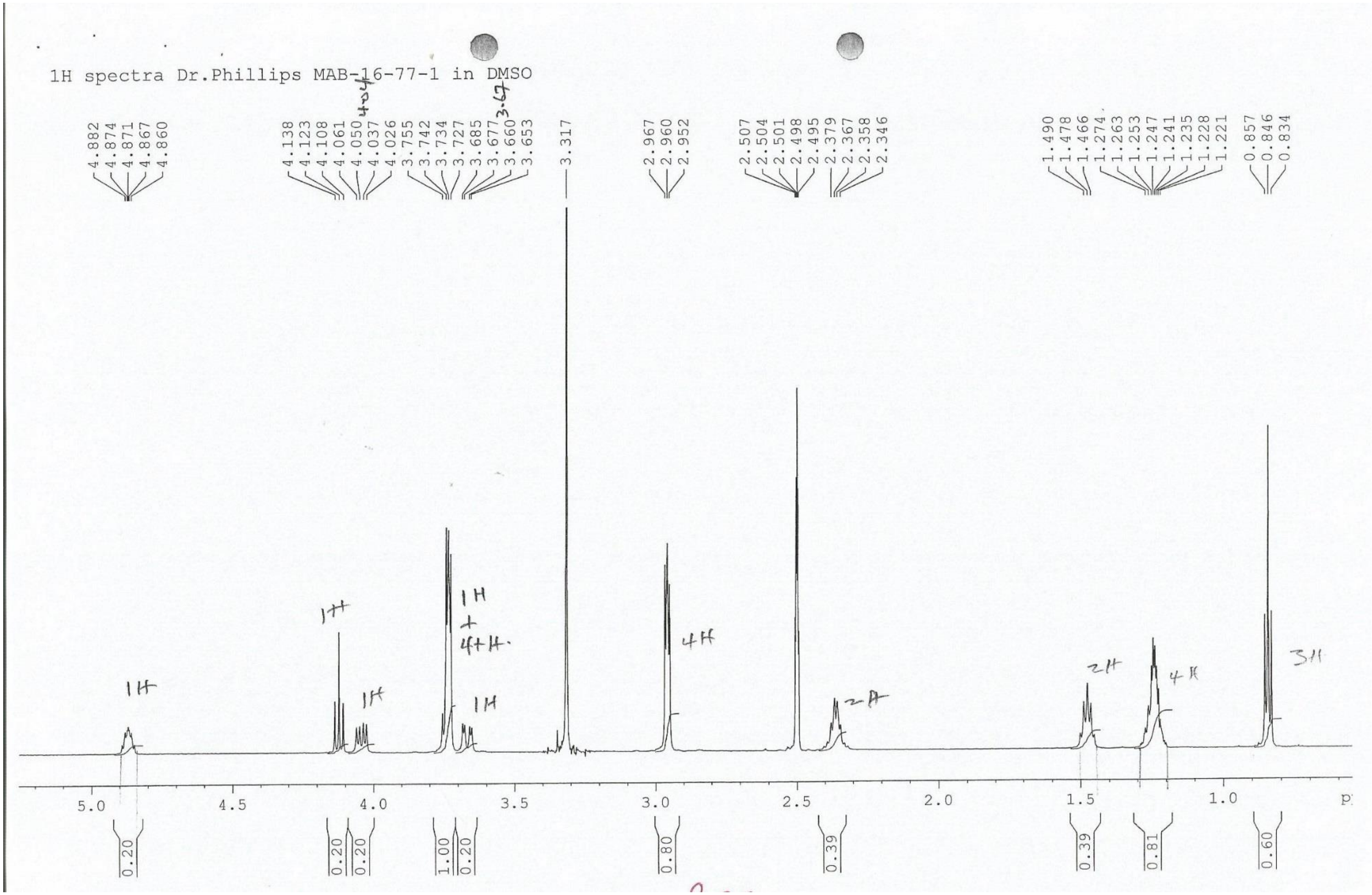
1H spectra Dr.Phillips MAB-16-77-1 in DMSO
4.882
4.874
4.871
4.867
4.860
4.138
4.123
4.108
4.061
4.050
4.037
4.026
3.755
3.742
3.734
3.727
3.685
3.677
3.660
3.653
3.317
2.967
2.960
2.952
2.507
2.504
2.501
2.498
2.495
2.379
2.367
2.358
2.346
1.490
1.478
1.466
1.274
1.263
1.253
1.247
1.241
1.235
1.228
1.221
0.857
0.846
0.834
5.0
4.5
4.0
3.5
3.0
2.5
2.0
1.5
1.0
0.20
0.20
0.20
1.00
0.20
0.80
0.39
0.39
0.81
0.60

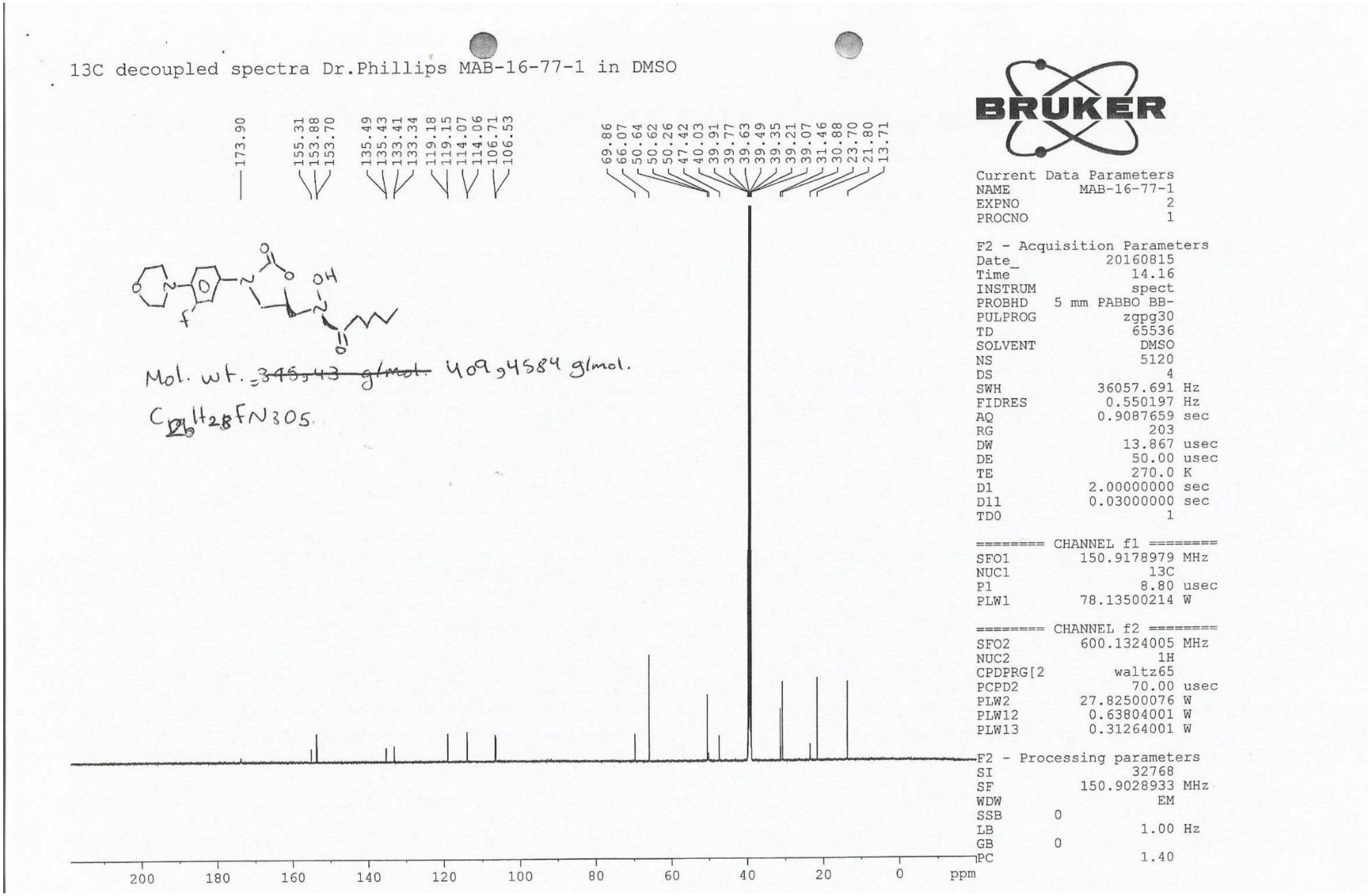
13C decoupled spectra Dr.Phillips MAB-16-77-1 in DMSO
173.90
155.31
153.88
153.70
135.49
135.43
133.41
133.34
119.18
119.15
114.07
114.06
106.71
106.53
69.86
66.07
50.64
50.62
50.26
47.42
40.03
39.91
39.77
39.63
39.49
39.35
39.21
39.07
31.46
30.88
23.70
21.80
13.71
Mol. wt. = 345,43 g/mol. 409,4584 g/mol.
BRUKER
Current Data Parameters
NAME MAB-16-77-1
EXPNO 2
PROCNO 1
F2 - Acquisition Parameters
Date_ 20160815
Time 14.16
INSTRUM spect
PROBHD 5 mm PABBO BB-
PULPROG zgpg30
TD 65536
SOLVENT DMSO
NS 5120
DS 4
SWH 36057.691 Hz
FIDRES 0.550197 Hz
AQ 0.9087659 sec
RG 203
DW 13.867 usec
DE 50.00 usec
TE 270.0 K
D1 2.00000000 sec
D11 0.03000000 sec
TD0 1
======== CHANNEL f1 ========
SFO1 150.9178979 MHz
NUC1 13C
P1 8.80 usec
PLW1 78.13500214 W
======== CHANNEL f2 ========
SFO2 600.1324005 MHz
NUC2 1H
CPDPRG[2 waltz65
PCPD2 70.00 usec
PLW2 27.82500076 W
PLW12 0.63804001 W
PLW13 0.31264001 W
F2 - Processing parameters
SI 32768
SF 150.9028933 MHz
WDW EM
SSB 0
LB 1.00 Hz
GB 0
PC 1.40
200
180
160
140
120
100
80
60
40
20
0
ppm

Research Sector Projects Unit GS 01/05
Kuwait University

RSPU
Research Sector Projects Unit
KUWAIT UNIVERSITY

FT- IR Spectral Data

PH247

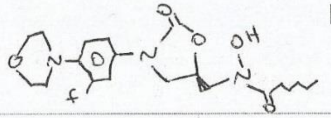

Mol. wt. = ~~395.43 g/mol.~~ 409.4584 g/mol.
$C_{20}H_{28}FN_3O_5$

PH247

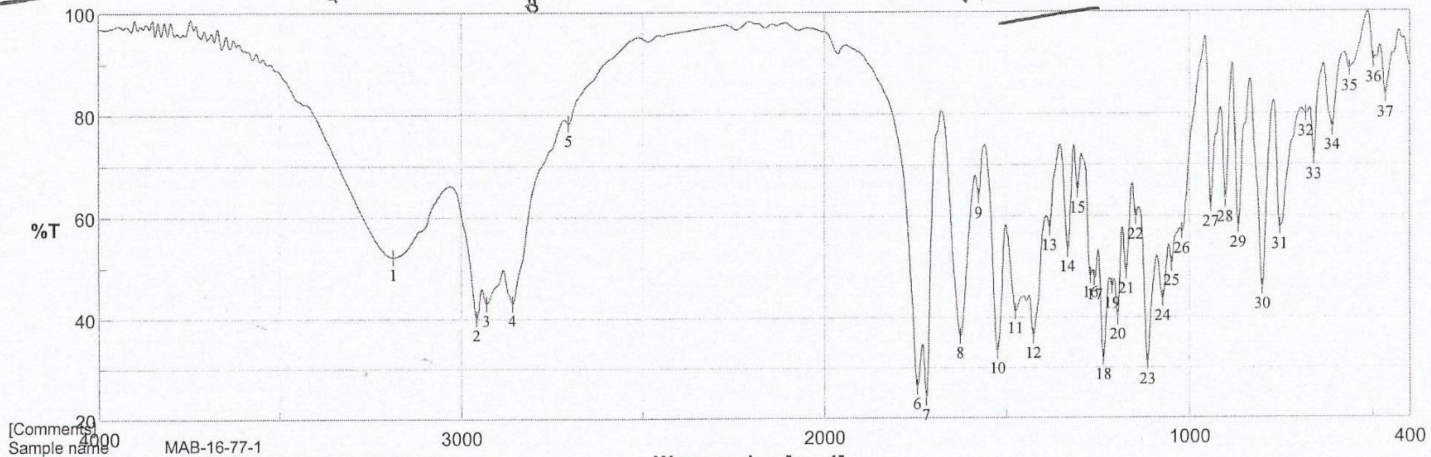

[Comments]

Sample name	MAB-16-77-1
Comment	PREPARED BY SHOBHA ON 17.8.16
User	PROF. O. A. PHILIPS
Division	RSPU GS01/05
Company	Kuwait University

[Measurement Information]

Model Name	FT/IR-6300typeA
Serial Number	A009861024
Light Source	Standard
Detector	TGS
Accumulation	Auto (41)
Resolution	4 cm-1
Zero Filling	On
Apodization	Cosine
Gain	Auto (4)
Aperture	Auto (7.1 mm)
Scanning Speed	Auto (2 mm/sec)
Filter	Auto (10000 Hz)

Result of Peak Picking

No.	Position	Intensity	No.	Position	Intensity	No.	Position	Intensity	No.	Position	Intensity
1	3186.79	52.1333	2	2957.3	39.9688	3	2930.31	42.9479	4	2857.99	42.9332
5	2703.71	78.2979	6	1744.3	26.0963	7	1719.23	24.0076	8	1625.7	36.1802
9	1575.56	63.8464	10	1524.45	33.1941	11	1475.28	41.1714	12	1426.1	36.2126
13	1380.78	57.6209	14	1331.61	53.3913	15	1305.57	64.8318	16	1269.9	48.2415
17	1258.32	47.7379	18	1234.22	31.9266	19	1211.08	46.155	20	1195.65	39.9185
21	1172.51	49.0343	22	1145.51	59.733	23	1113.69	30.9521	24	1073.19	43.5593
25	1048.12	50.6022	26	1019.19	56.8875	27	941.092	62.173	28	901.558	62.9701
29	865.882	58.0166	30	801.278	45.8973	31	753.066	57.8122	32	682.677	79.8512
33	660.5	71.3811	34	610.36	77.1251	35	565.041	88.6694	36	499.473	90.2805
37	466.689	83.4458									

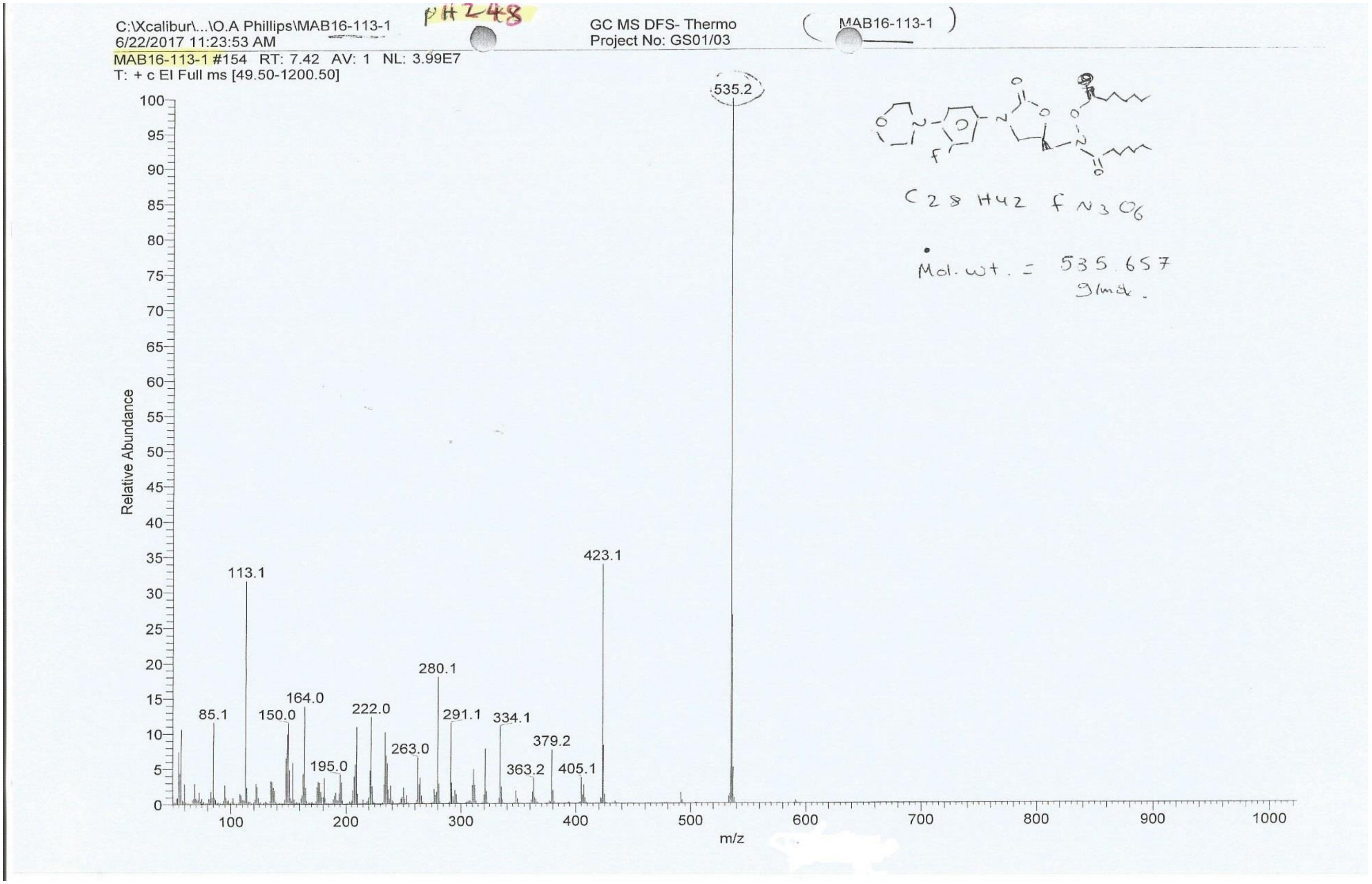
C:\Xcalibur\...\O.A Phillips\MAB16-113-1
6/22/2017 11:23:53 AM
PH248
GC MS DFS- Thermo
Project No: GS01/03
MAB16-113-1
MAB16-113-1 #154 RT: 7.42 AV: 1 NL: 3.99E7
T: + c EI Full ms [49.50-1200.50]
Relative Abundance
m/z
535.2
423.1
113.1
280.1
164.0
85.1
150.0
222.0
291.1
334.1
379.2
263.0
195.0
363.2
405.1
C28 H42 F N3 O6
Mol. wt. = 535.657 g/mole.

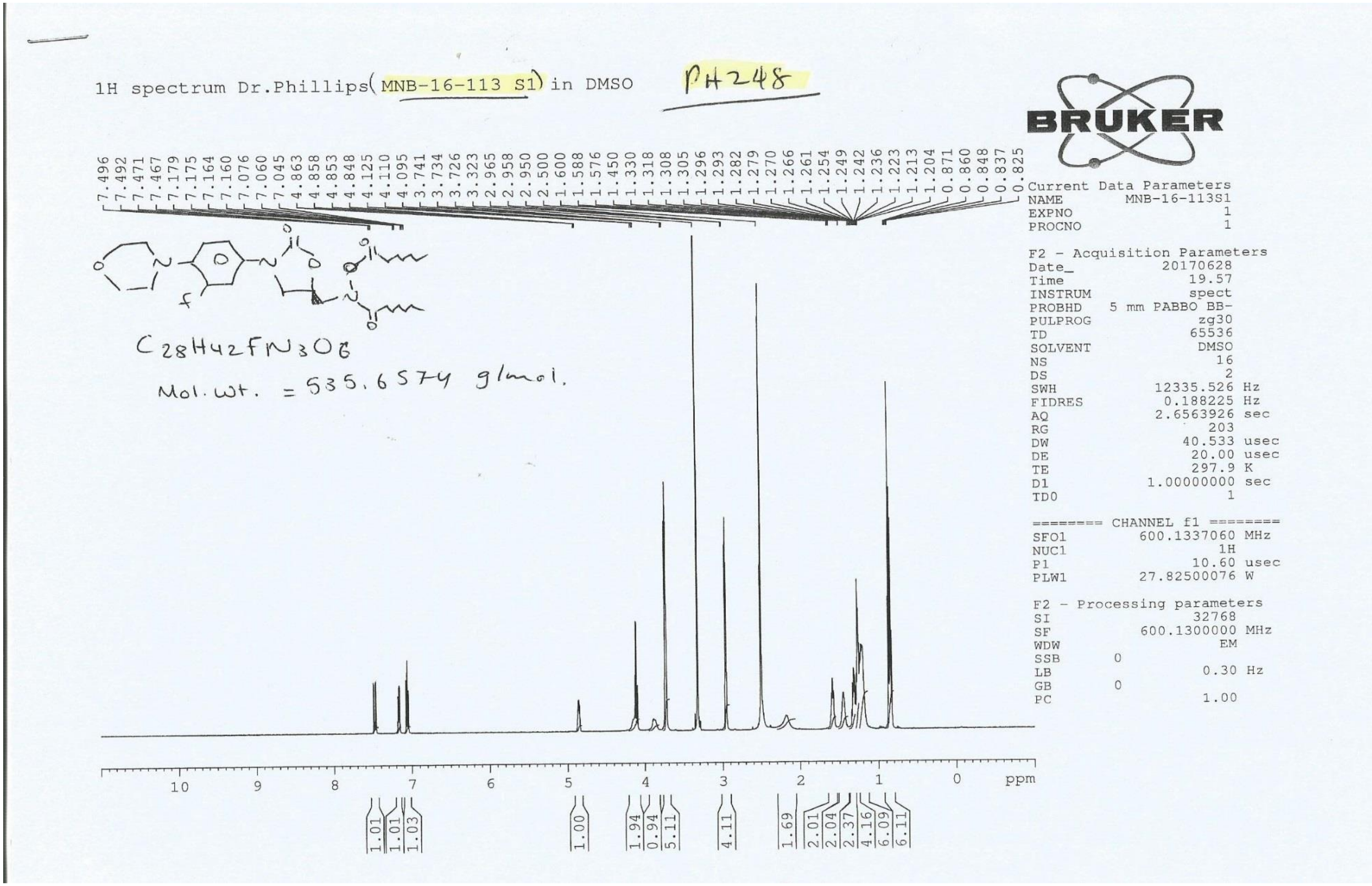

1H spectrum Dr.Phillips(MNB-16-113 S1) in DMSO
PH248
BRUKER
7.496 7.492 7.471 7.467 7.179 7.175 7.164 7.160 7.076 7.060 7.045 4.863 4.858 4.853 4.848 4.125 4.110 4.095 3.741 3.734 3.726 3.323 2.965 2.958 2.950 2.500 1.600 1.588 1.576 1.450 1.330 1.318 1.308 1.305 1.296 1.293 1.282 1.279 1.270 1.266 1.261 1.254 1.249 1.242 1.236 1.223 1.213 1.204 0.871 0.860 0.848 0.837 0.825
C28H42FN3O6
Mol. wt. = 535.6574 g/mol.
Current Data Parameters
NAME MNB-16-113S1
EXPNO 1
PROCNO 1
F2 - Acquisition Parameters
Date_ 20170628
Time 19.57
INSTRUM spect
PROBHD 5 mm PABBO BB-
PULPROG zg30
TD 65536
SOLVENT DMSO
NS 16
DS 2
SWH 12335.526 Hz
FIDRES 0.188225 Hz
AQ 2.6563926 sec
RG 203
DW 40.533 usec
DE 20.00 usec
TE 297.9 K
D1 1.00000000 sec
TD0 1
======== CHANNEL f1 ========
SFO1 600.1337060 MHz
NUC1 1H
P1 10.60 usec
PLW1 27.82500076 W
F2 - Processing parameters
SI 32768
SF 600.1300000 MHz
WDW EM
SSB 0
LB 0.30 Hz
GB 0
PC 1.00
10 9 8 7 6 5 4 3 2 1 0 ppm
1.01 1.01 1.03 1.00 1.94 0.94 5.11 4.11 1.69 2.01 2.04 2.37 4.16 6.09 6.11

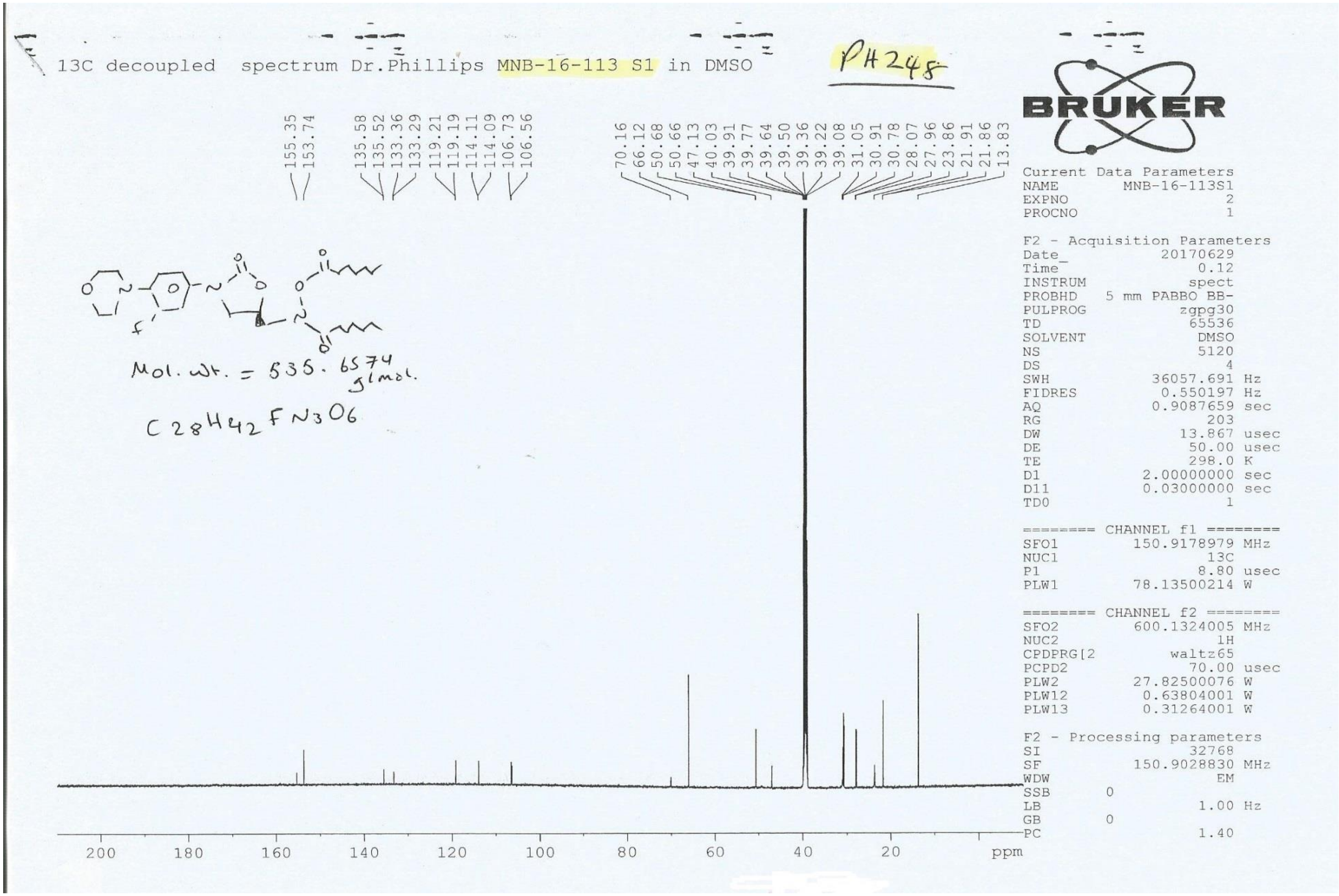

13C decoupled spectrum Dr.Phillips MNB-16-113 S1 in DMSO
PH248
BRUKER
155.35
153.74
135.58
135.52
133.36
133.29
119.21
119.19
114.11
114.09
106.73
106.56
70.16
66.12
50.68
50.66
47.13
40.03
39.91
39.77
39.64
39.50
39.36
39.22
39.08
31.05
30.91
30.78
28.07
27.96
23.86
21.91
21.86
13.83
Mol. wt. = 535.6574 g/mol.
C28H42FN3O6
Current Data Parameters
NAME MNB-16-113S1
EXPNO 2
PROCNO 1
F2 - Acquisition Parameters
Date_ 20170629
Time 0.12
INSTRUM spect
PROBHD 5 mm PABBO BB-
PULPROG zgpg30
TD 65536
SOLVENT DMSO
NS 5120
DS 4
SWH 36057.691 Hz
FIDRES 0.550197 Hz
AQ 0.9087659 sec
RG 203
DW 13.867 usec
DE 50.00 usec
TE 298.0 K
D1 2.00000000 sec
D11 0.03000000 sec
TD0 1
======== CHANNEL f1 ========
SFO1 150.9178979 MHz
NUC1 13C
P1 8.80 usec
PLW1 78.13500214 W
======== CHANNEL f2 ========
SFO2 600.1324005 MHz
NUC2 1H
CPDPRG[2 waltz65
PCPD2 70.00 usec
PLW2 27.82500076 W
PLW12 0.63804001 W
PLW13 0.31264001 W
F2 - Processing parameters
SI 32768
SF 150.9028830 MHz
WDW EM
SSB 0
LB 1.00 Hz
GB 0
PC 1.40
200
180
160
140
120
100
80
60
40
20
ppm

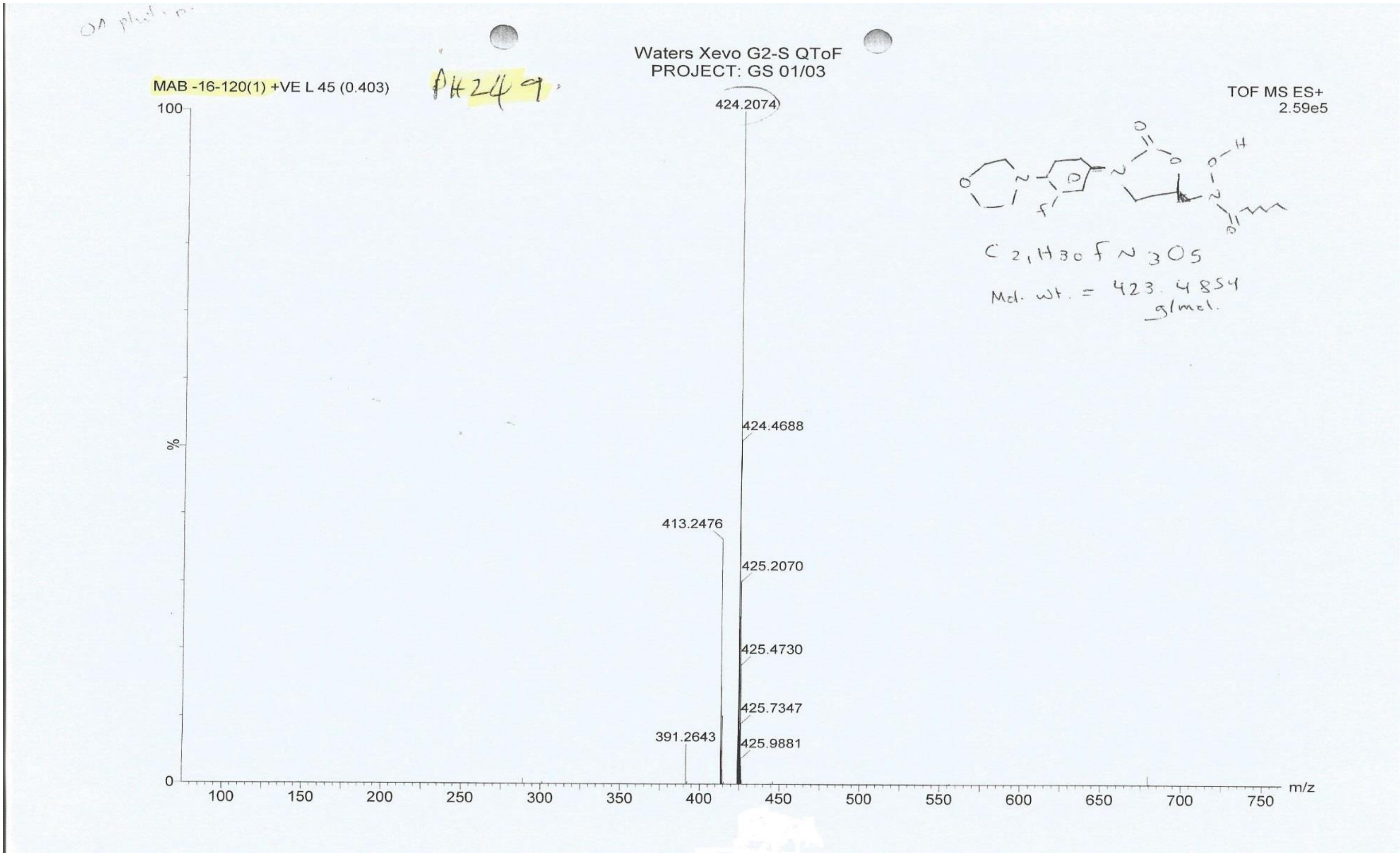
Waters Xevo G2-S QToF
PROJECT: GS 01/03
MAB -16-120(1) +VE L 45 (0.403)
PH249
TOF MS ES+
2.59e5
424.2074
424.4688
413.2476
425.2070
425.4730
425.7347
391.2643
425.9881
100
%
0
100
150
200
250
300
350
400
450
500
550
600
650
700
750
m/z
C21H30FN3O5
Mol. wt. = 423.4854 g/mol.

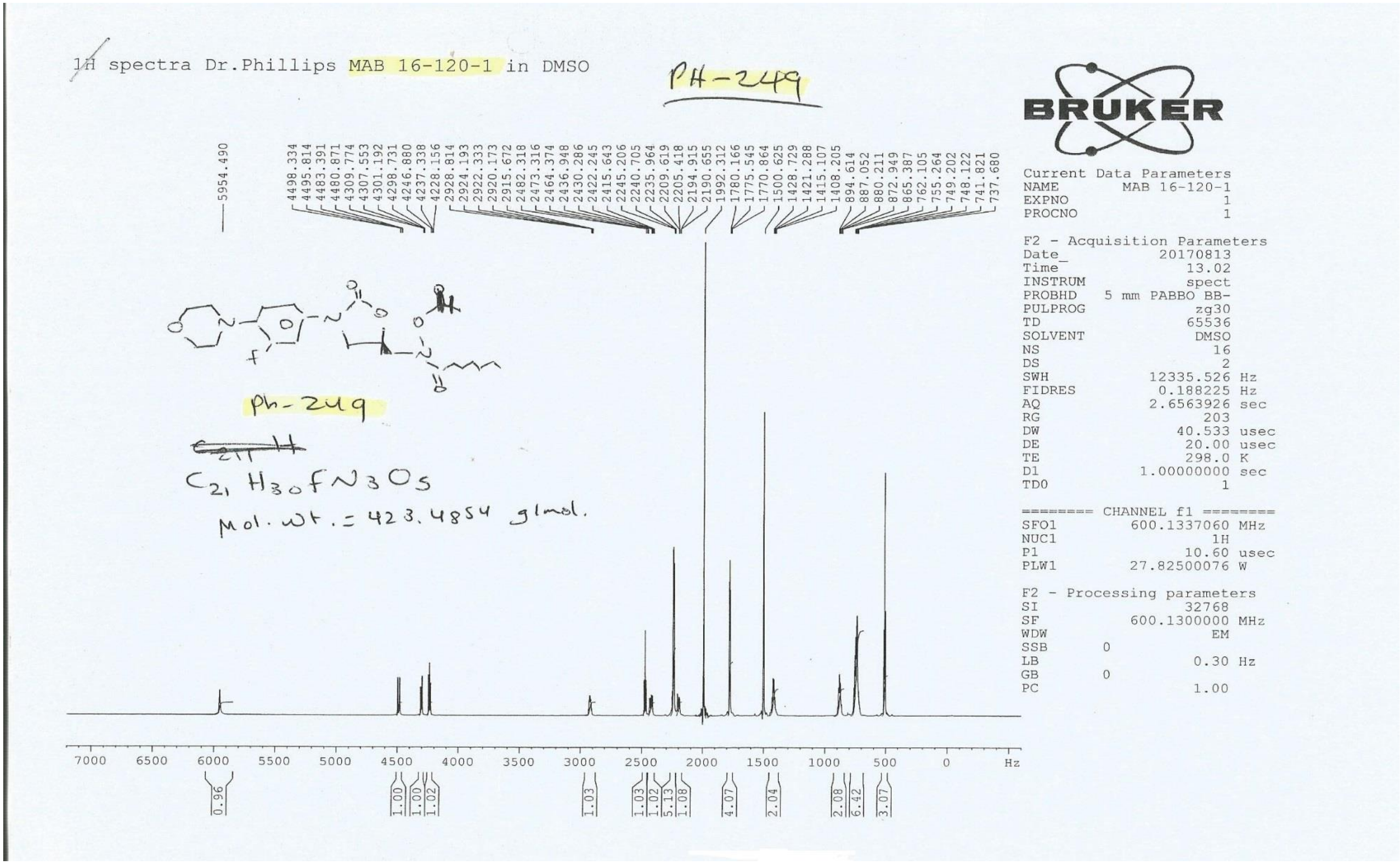
1H spectra Dr.Phillips MAB 16-120-1 in DMSO
PH-249
BRUKER
5954.490
4498.334
4495.814
4483.391
4480.871
4309.774
4307.553
4301.192
4298.731
4246.880
4237.338
4228.156
2928.814
2924.193
2922.333
2920.173
2915.672
2482.318
2473.316
2464.374
2436.948
2430.286
2422.245
2415.643
2245.206
2240.705
2235.964
2209.619
2205.418
2194.915
2190.655
1992.312
1780.166
1775.545
1770.864
1500.625
1428.729
1421.288
1415.107
1408.205
894.614
887.052
880.211
872.949
865.387
762.105
755.264
749.202
748.122
741.821
737.680
Ph-249
C21 H30 F N3 O5
Mol. Wt. = 423.4854 g/mol.
Current Data Parameters
NAME MAB 16-120-1
EXPNO 1
PROCNO 1
F2 - Acquisition Parameters
Date_ 20170813
Time 13.02
INSTRUM spect
PROBHD 5 mm PABBO BB-
PULPROG zg30
TD 65536
SOLVENT DMSO
NS 16
DS 2
SWH 12335.526 Hz
FIDRES 0.188225 Hz
AQ 2.6563926 sec
RG 203
DW 40.533 usec
DE 20.00 usec
TE 298.0 K
D1 1.00000000 sec
TD0 1
======== CHANNEL f1 ========
SFO1 600.1337060 MHz
NUC1 1H
P1 10.60 usec
PLW1 27.82500076 W
F2 - Processing parameters
SI 32768
SF 600.1300000 MHz
WDW EM
SSB 0
LB 0.30 Hz
GB 0
PC 1.00
7000 6500 6000 5500 5000 4500 4000 3500 3000 2500 2000 1500 1000 500 0 Hz
0.96
1.00
1.00
1.02
1.03
1.03
1.02
5.13
1.08
4.07
2.04
2.08
6.42
3.07

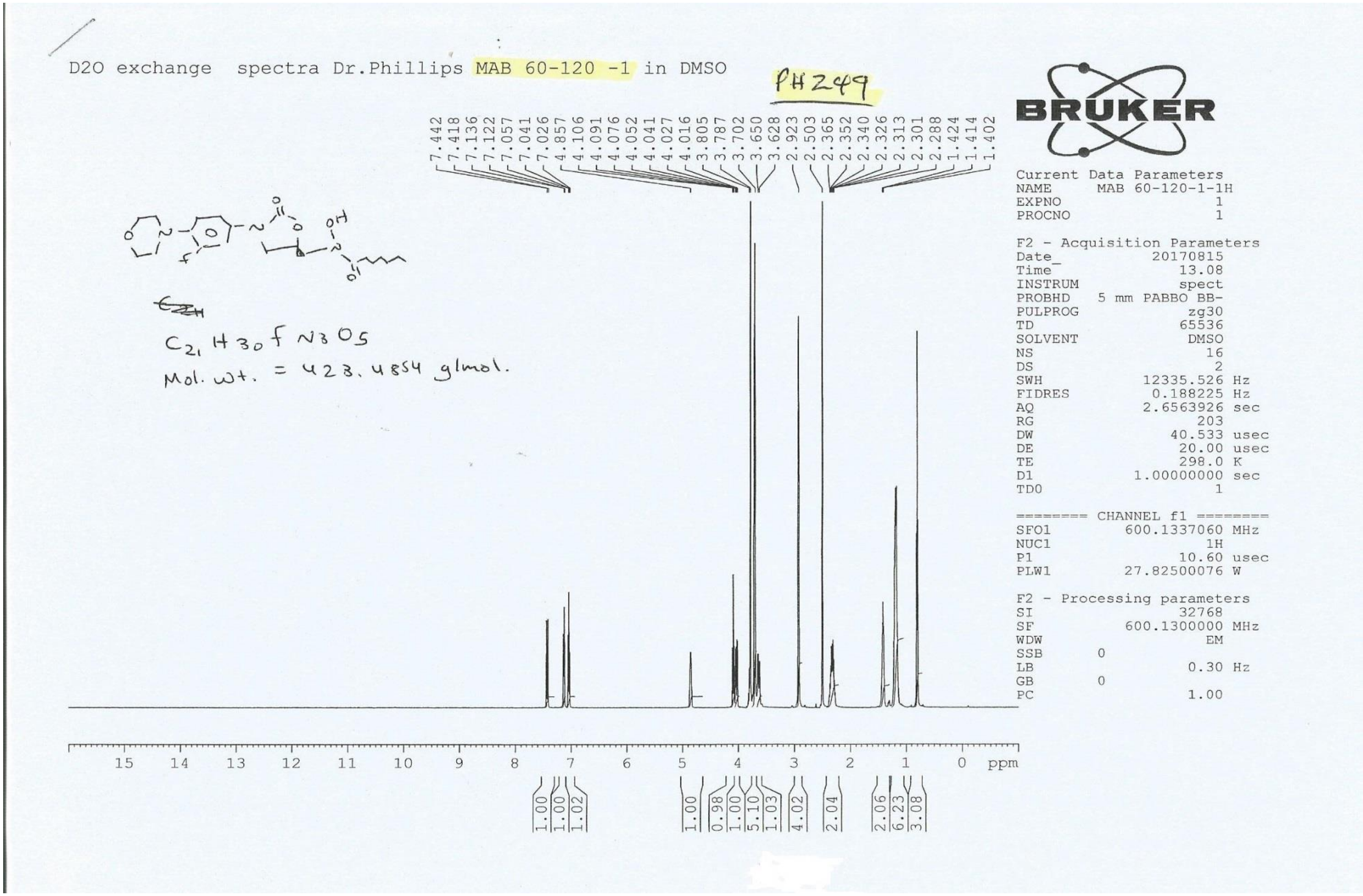
D2O exchange spectra Dr.Phillips MAB 60-120 -1 in DMSO
PH249
7.442
7.418
7.136
7.122
7.057
7.041
7.026
4.857
4.106
4.091
4.076
4.052
4.041
4.027
4.016
3.805
3.787
3.702
3.650
3.628
2.923
2.503
2.365
2.352
2.340
2.326
2.313
2.301
2.288
1.424
1.414
1.402
BRUKER
Current Data Parameters
NAME MAB 60-120-1-1H
EXPNO 1
PROCNO 1
F2 - Acquisition Parameters
Date_ 20170815
Time 13.08
INSTRUM spect
PROBHD 5 mm PABBO BB-
PULPROG zg30
TD 65536
SOLVENT DMSO
NS 16
DS 2
SWH 12335.526 Hz
FIDRES 0.188225 Hz
AQ 2.6563926 sec
RG 203
DW 40.533 usec
DE 20.00 usec
TE 298.0 K
D1 1.00000000 sec
TD0 1
======== CHANNEL f1 ========
SFO1 600.1337060 MHz
NUC1 1H
P1 10.60 usec
PLW1 27.82500076 W
F2 - Processing parameters
SI 32768
SF 600.1300000 MHz
WDW EM
SSB 0
LB 0.30 Hz
GB 0
PC 1.00
C21 H30 F N3 O5
Mol. wt. = 423.4854 g/mol.
15 14 13 12 11 10 9 8 7 6 5 4 3 2 1 0 ppm
1.00
1.00
1.02
1.00
0.98
1.00
5.10
1.03
4.02
2.04
2.06
6.23
3.08

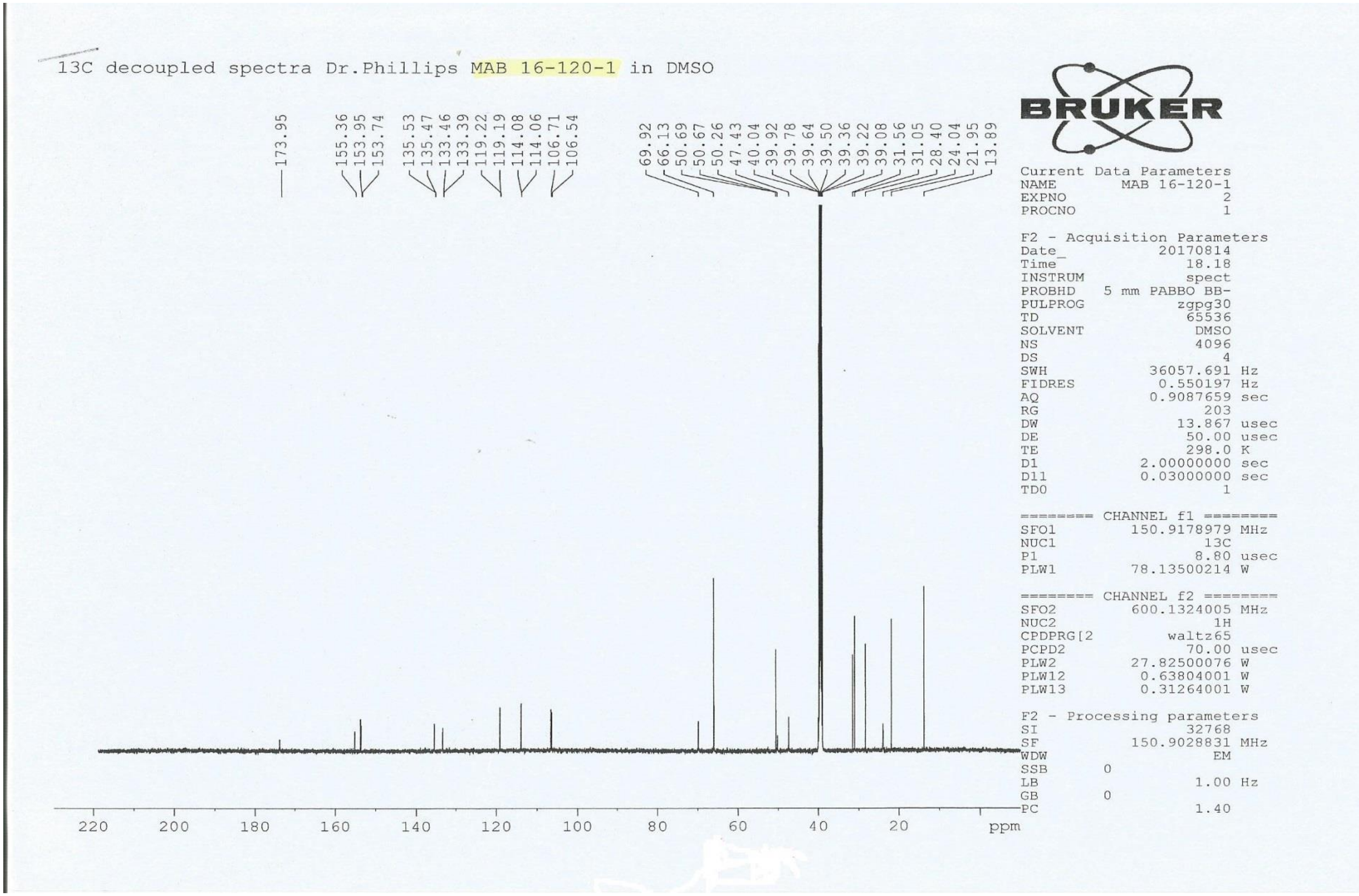
13C decoupled spectra Dr.Phillips MAB 16-120-1 in DMSO
173.95
155.36
153.95
153.74
135.53
135.47
133.46
133.39
119.22
119.19
114.08
114.06
106.71
106.54
69.92
66.13
50.69
50.67
50.26
47.43
40.04
39.92
39.78
39.64
39.50
39.36
39.22
39.08
31.56
31.05
28.40
24.04
21.95
13.89
BRUKER
Current Data Parameters
NAME MAB 16-120-1
EXPNO 2
PROCNO 1
F2 - Acquisition Parameters
Date_ 20170814
Time 18.18
INSTRUM spect
PROBHD 5 mm PABBO BB-
PULPROG zgpg30
TD 65536
SOLVENT DMSO
NS 4096
DS 4
SWH 36057.691 Hz
FIDRES 0.550197 Hz
AQ 0.9087659 sec
RG 203
DW 13.867 usec
DE 50.00 usec
TE 298.0 K
D1 2.00000000 sec
D11 0.03000000 sec
TD0 1
======== CHANNEL f1 ========
SFO1 150.9178979 MHz
NUC1 13C
P1 8.80 usec
PLW1 78.13500214 W
======== CHANNEL f2 ========
SFO2 600.1324005 MHz
NUC2 1H
CPDPRG[2 waltz65
PCPD2 70.00 usec
PLW2 27.82500076 W
PLW12 0.63804001 W
PLW13 0.31264001 W
F2 - Processing parameters
SI 32768
SF 150.9028831 MHz
WDW EM
SSB 0
LB 1.00 Hz
GB 0
PC 1.40
220 200 180 160 140 120 100 80 60 40 20 ppm

Research Sector Projects U[illegible] GS 01/05
Kuwait University

Pit 249

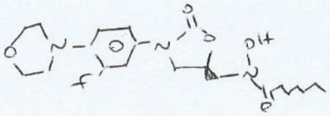

FT- IR Spectral Data

$C_{21}H_{30}FN_3O_5$

Mol. wt. = 423.4854 g/mol.

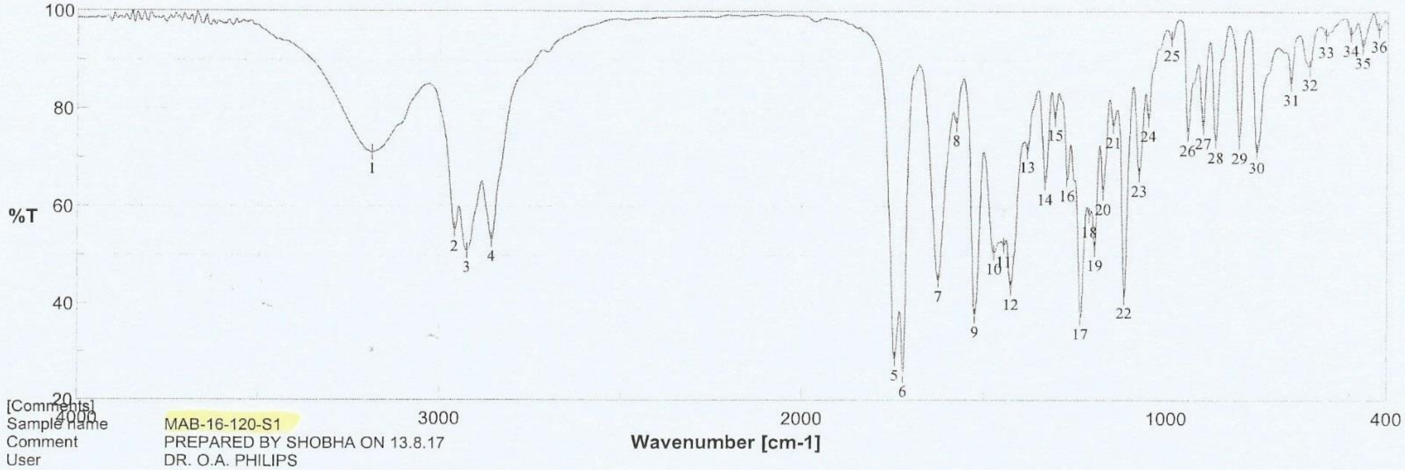

[Comments]

Sample name	MAB-16-120-S1
Comment	PREPARED BY SHOBHA ON 13.8.17
User	DR. O.A. PHILIPS
Division	RSPU GS01/05
Company	Kuwait University

[Measurement Information]

Model Name	FT/IR-6300typeA
Serial Number	A009861024
Light Source	Standard
Detector	TGS
Accumulation	Auto (53)
Resolution	4 cm-1
Zero Filling	On
Apodization	Cosine
Gain	Auto (4)
Aperture	Auto (7.1 mm)
Scanning Speed	Auto (2 mm/sec)
Filter	Auto (10000 Hz)

Result of Peak Picking

No.	Position	Intensity	No.	Position	Intensity	No.	Position	Intensity	No.	Position	Intensity
1	3184.86	71.0032	2	2957.3	55.1152	3	2923.56	50.8758	4	2856.06	53.0675
5	1743.33	28.4491	6	1720.19	25.1805	7	1624.73	44.8624	8	1575.56	76.7535
9	1524.45	37.523	10	1472.38	50.5741	11	1445.39	52.0609	12	1426.1	43.1865
13	1380.78	71.3225	14	1331.61	64.7881	15	1305.57	78.0044	16	1271.82	65.496
17	1235.18	37.0884	18	1211.08	58.1236	19	1196.61	51.3345	20	1173.47	62.8009
21	1146.47	76.3731	22	1114.65	41.4938	23	1074.16	66.5285	24	1049.09	77.7669
25	987.375	94.3652	26	942.056	74.8755	27	900.594	76.318	28	865.882	73.3948
29	801.278	73.2559	30	753.066	71.3295	31	661.464	85.2694	32	611.324	88.422
33	566.969	95.1517	34	500.437	95.414	35	467.653	92.9364	36	424.263	96.3575

RESEARCH SECTOR PROJECTS UNIT
PROJECT – GS 01/03
Faculty of Science

RSPU Research Sector Projects Unit KUWAIT UNIVERSITY

INSTRUMENT NAME: LC- MS/MS (Xevo G2-S QToF)   INSTRUMENT PROJECT No: GS 01/03

ANALYST NAME: DANGLY ANN JACOB

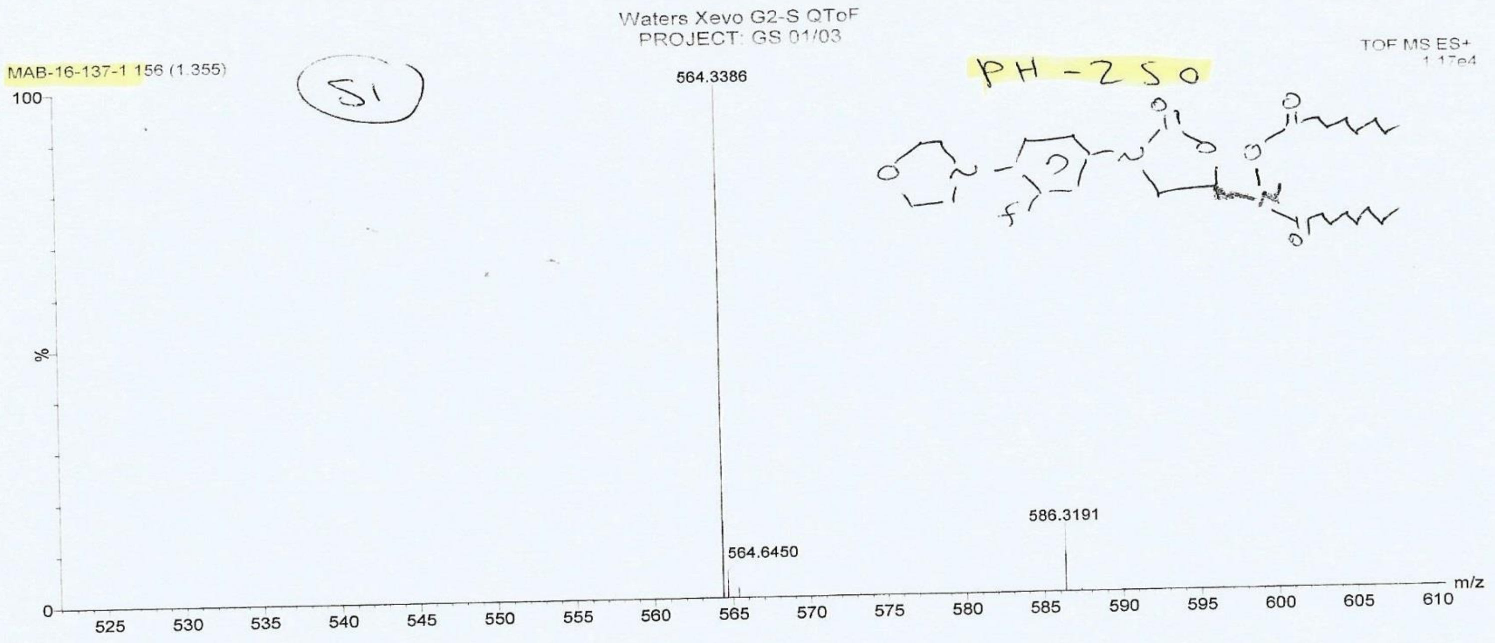

***Please acknowledge RESEARCH SECTOR PROJECT NUMBER (GS 01/03) in PUBLICATIONS.**

*** Please visit our website www.science.saf.kuniv.edu for more information on RSPU facilities.**

***Please collect your samples within one week after collecting the results.**

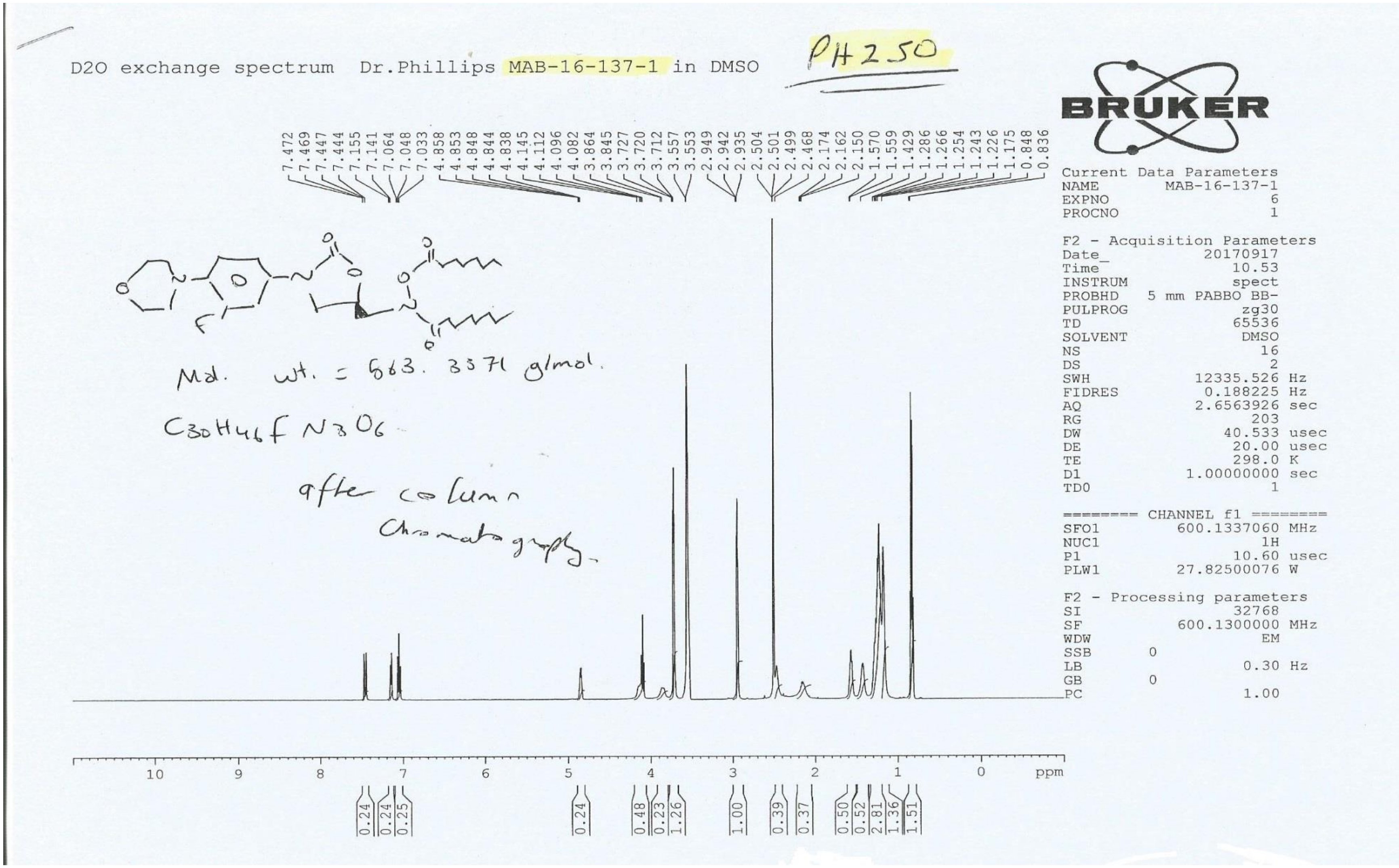
D2O exchange spectrum Dr.Phillips MAB-16-137-1 in DMSO
PH250
BRUKER
7.472 7.469 7.447 7.444 7.155 7.141 7.064 7.048 7.033 4.858 4.853 4.848 4.844 4.838 4.145 4.112 4.096 4.082 3.864 3.845 3.727 3.720 3.712 3.557 3.553 2.949 2.942 2.935 2.504 2.501 2.499 2.468 2.174 2.162 2.150 1.570 1.559 1.429 1.286 1.266 1.254 1.243 1.226 1.175 0.848 0.836
Mol. wt. = 563.3371 g/mol.
C30H46F N3O6
after column chromatography
Current Data Parameters
NAME MAB-16-137-1
EXPNO 6
PROCNO 1
F2 - Acquisition Parameters
Date_ 20170917
Time 10.53
INSTRUM spect
PROBHD 5 mm PABBO BB-
PULPROG zg30
TD 65536
SOLVENT DMSO
NS 16
DS 2
SWH 12335.526 Hz
FIDRES 0.188225 Hz
AQ 2.6563926 sec
RG 203
DW 40.533 usec
DE 20.00 usec
TE 298.0 K
D1 1.00000000 sec
TD0 1
======== CHANNEL f1 ========
SFO1 600.1337060 MHz
NUC1 1H
P1 10.60 usec
PLW1 27.82500076 W
F2 - Processing parameters
SI 32768
SF 600.1300000 MHz
WDW EM
SSB 0
LB 0.30 Hz
GB 0
PC 1.00
10 9 8 7 6 5 4 3 2 1 0 ppm
0.24 0.24 0.25 0.24 0.48 0.23 1.26 1.00 0.39 0.37 0.50 0.52 2.81 1.36 1.51

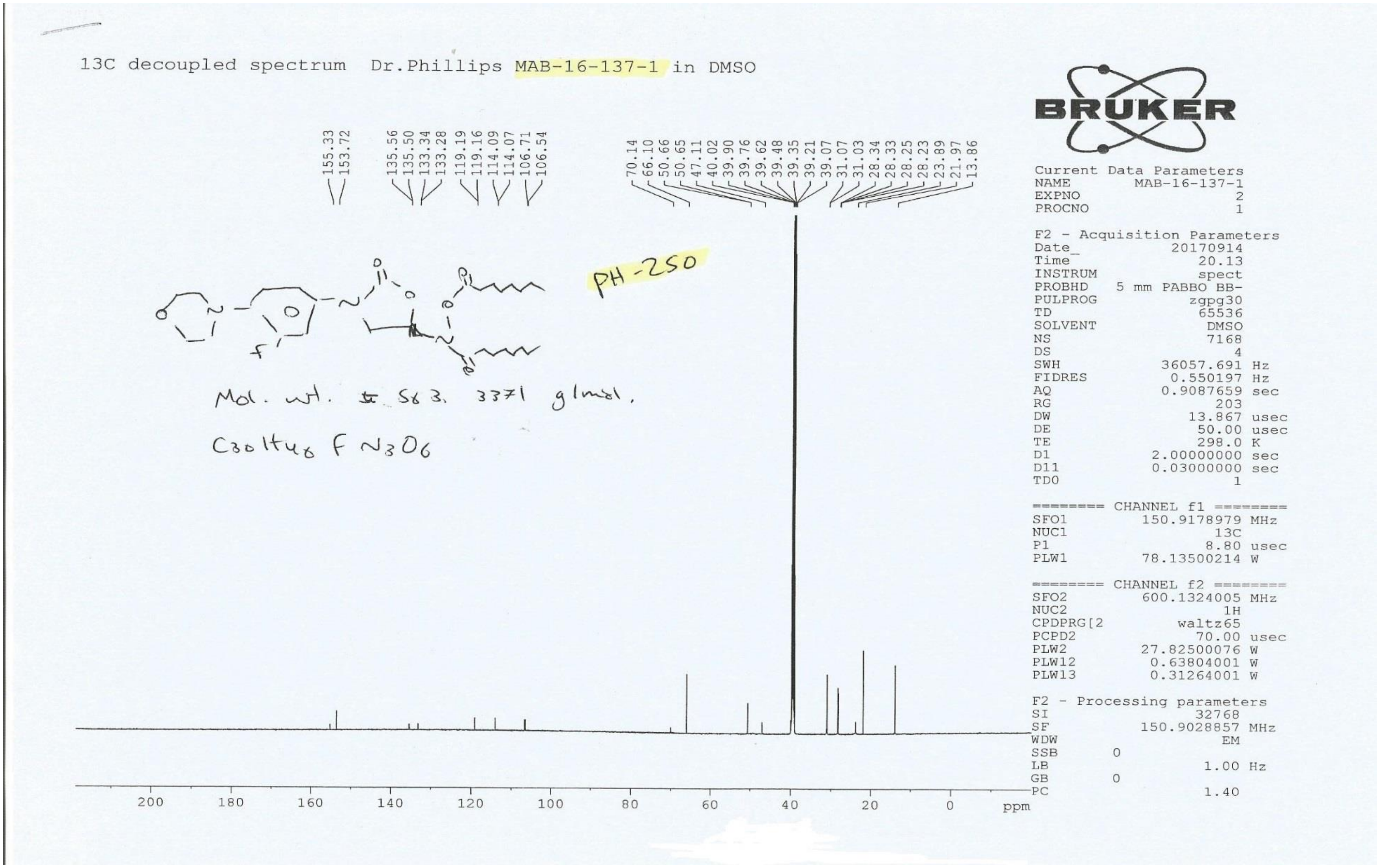
13C decoupled spectrum Dr.Phillips MAB-16-137-1 in DMSO
155.33
153.72
135.56
135.50
133.34
133.28
119.19
119.16
114.09
114.07
106.71
106.54
70.14
66.10
50.66
50.65
47.11
40.02
39.90
39.76
39.62
39.48
39.35
39.21
39.07
31.07
31.03
28.34
28.33
28.25
28.23
23.89
21.97
13.86
PH-250
Mol. wt. 563.3371 g/mol.
C30H48 F N3O6
BRUKER
Current Data Parameters
NAME MAB-16-137-1
EXPNO 2
PROCNO 1
F2 - Acquisition Parameters
Date_ 20170914
Time 20.13
INSTRUM spect
PROBHD 5 mm PABBO BB-
PULPROG zgpg30
TD 65536
SOLVENT DMSO
NS 7168
DS 4
SWH 36057.691 Hz
FIDRES 0.550197 Hz
AQ 0.9087659 sec
RG 203
DW 13.867 usec
DE 50.00 usec
TE 298.0 K
D1 2.00000000 sec
D11 0.03000000 sec
TD0 1
======== CHANNEL f1 ========
SFO1 150.9178979 MHz
NUC1 13C
P1 8.80 usec
PLW1 78.13500214 W
======== CHANNEL f2 ========
SFO2 600.1324005 MHz
NUC2 1H
CPDPRG[2 waltz65
PCPD2 70.00 usec
PLW2 27.82500076 W
PLW12 0.63804001 W
PLW13 0.31264001 W
F2 - Processing parameters
SI 32768
SF 150.9028857 MHz
WDW EM
SSB 0
LB 1.00 Hz
GB 0
PC 1.40
200
180
160
140
120
100
80
60
40
20
0
ppm

Research Sector Projects Unit GS 01/05
Kuwait University

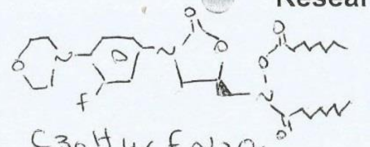

FT- IR Spectral Data

PH 25[illegible]

RSPU
Research Sector Projects Unit
KUWAIT UNIVERSITY

$C_{30}H_{46}FN_3O_6$
Mol. Wt. = 563.7114 g/mol.

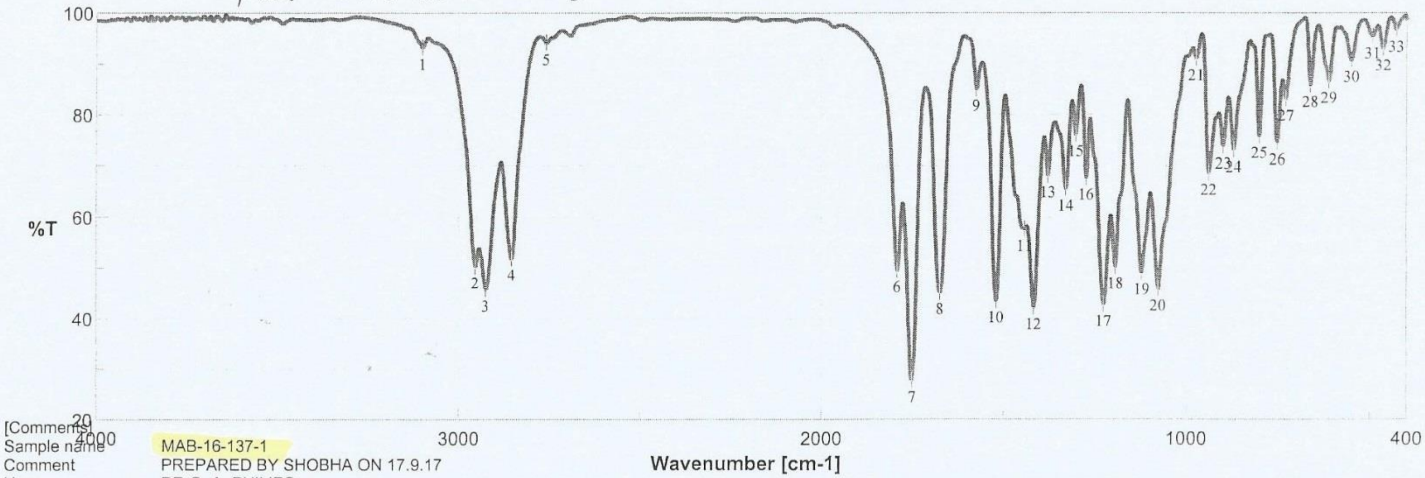

[Comments]

Sample name	MAB-16-137-1
Comment	PREPARED BY SHOBHA ON 17.9.17
User	DR.O. A. PHILIPS
Division	RSPU GS01/05
Company	Kuwait University

[Measurement Information]

Model Name	FT/IR-6300typeA
Serial Number	A009861024
Light Source	Standard
Detector	TGS
Accumulation	Auto (106)
Resolution	4 cm-1
Zero Filling	On
Apodization	Cosine
Gain	Auto (8)
Aperture	Auto (7.1 mm)
Scanning Speed	Auto (2 mm/sec)
Filter	Auto (10000 Hz)

Result of Peak Picking

No.	Position	Intensity	No.	Position	Intensity	No.	Position	Intensity	No.	Position	Intensity
1	3100.01	93.2111	2	2953.45	50.3185	3	2924.52	45.7631	4	2853.17	51.7983
5	2758.67	94.1325	6	1789.62	49.4951	7	1750.08	27.774	8	1672.95	45.1047
9	1573.63	85.0131	10	1519.63	43.536	11	1441.53	57.678	12	1416.46	42.1453
13	1376.93	68.1378	14	1327.75	65.5575	15	1300.75	76.0745	16	1271.82	67.6741
17	1224.58	42.693	18	1192.76	50.1646	19	1120.44	49.0599	20	1075.12	45.8001
21	971.947	91.1054	22	937.235	68.6784	23	897.701	73.7957	24	868.774	73.1181
25	799.35	75.7968	26	751.138	74.5249	27	727.996	83.1362	28	660.5	85.7831
29	610.36	86.5896	30	549.613	90.7758	31	492.723	95.4134	32	463.796	93.0075
33	429.084	96.7008									

RESEARCH SECTOR PROJECTS UNIT
PROJECT – GS 01/03
Faculty of Science

RSPU
Research Sector Projects Unit
KUWAIT UNIVERSITY

INSTRUMENT NAME: LC- MS/MS (Xevo G2-S QToF)
INSTRUMENT PROJECT No: GS 01/03
ANALYST NAME: DANGLY ANN JACOB

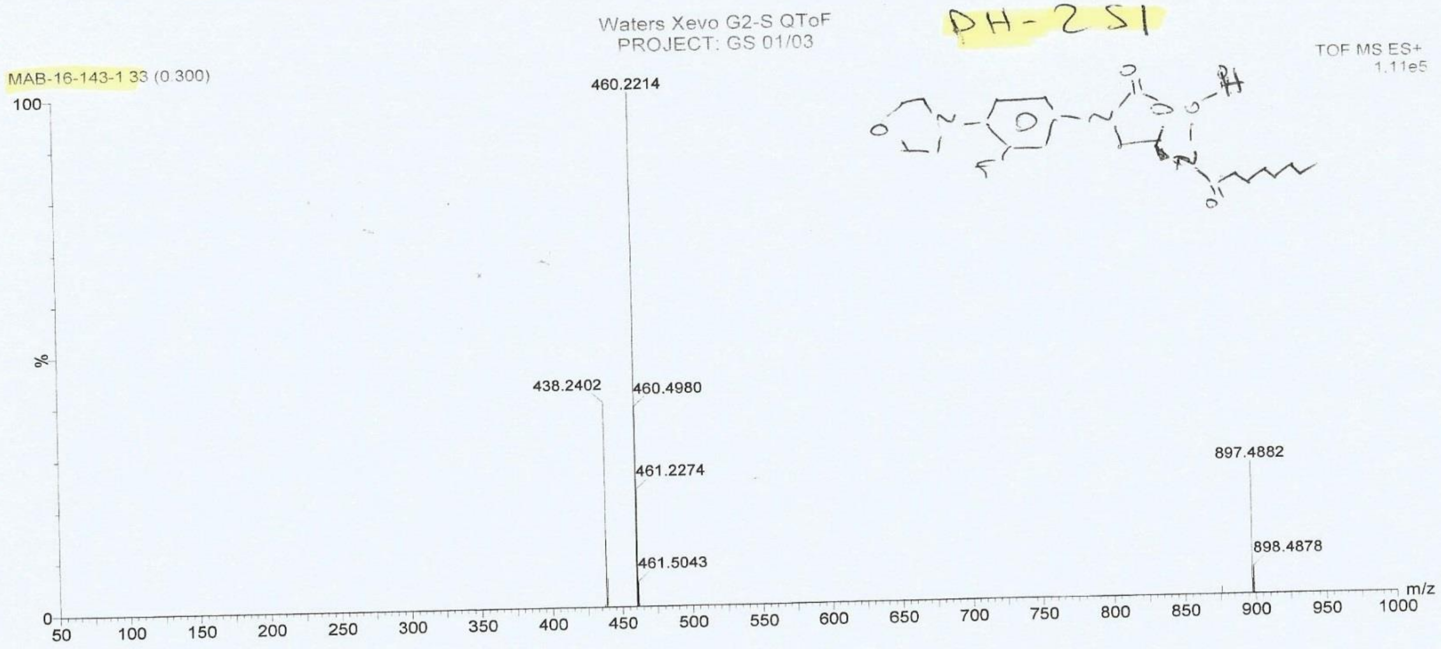

***Please acknowledge RESEARCH SECTOR PROJECT NUMBER (GS 01/03) in PUBLICATIONS.**
* *Please visit our website* ***www.science.saf.kuniv.edu*** *for more information on RSPU facilities.*
***Please collect your samples within one week after collecting the results.**

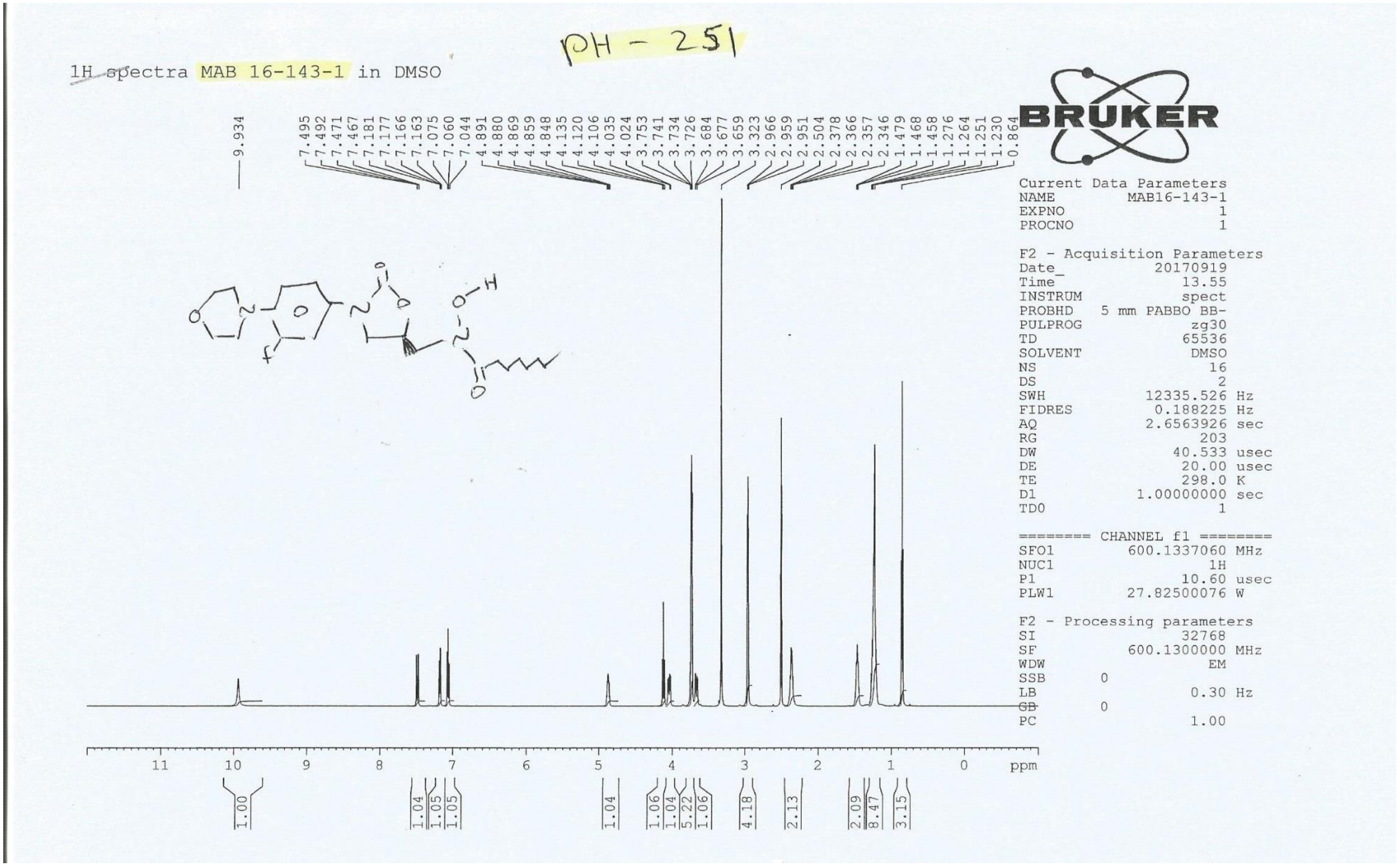
PH - 251
1H spectra MAB 16-143-1 in DMSO
9.934
7.495 7.492 7.471 7.467 7.181 7.177 7.166 7.163 7.075 7.060 7.044 4.891 4.880 4.869 4.859 4.848 4.135 4.120 4.106 4.035 4.024 3.753 3.741 3.734 3.726 3.684 3.677 3.659 3.323 2.966 2.959 2.951 2.504 2.378 2.366 2.357 2.346 1.479 1.468 1.458 1.276 1.264 1.251 1.230 0.864
BRUKER
Current Data Parameters
NAME MAB16-143-1
EXPNO 1
PROCNO 1
F2 - Acquisition Parameters
Date_ 20170919
Time_ 13.55
INSTRUM spect
PROBHD 5 mm PABBO BB-
PULPROG zg30
TD 65536
SOLVENT DMSO
NS 16
DS 2
SWH 12335.526 Hz
FIDRES 0.188225 Hz
AQ 2.6563926 sec
RG 203
DW 40.533 usec
DE 20.00 usec
TE 298.0 K
D1 1.00000000 sec
TD0 1
======== CHANNEL f1 ========
SFO1 600.1337060 MHz
NUC1 1H
P1 10.60 usec
PLW1 27.82500076 W
F2 - Processing parameters
SI 32768
SF 600.1300000 MHz
WDW EM
SSB 0
LB 0.30 Hz
GB 0
PC 1.00
11 10 9 8 7 6 5 4 3 2 1 0 ppm
1.00 1.04 1.05 1.05 1.04 1.06 1.04 5.22 1.06 4.18 2.13 2.09 8.47 3.15

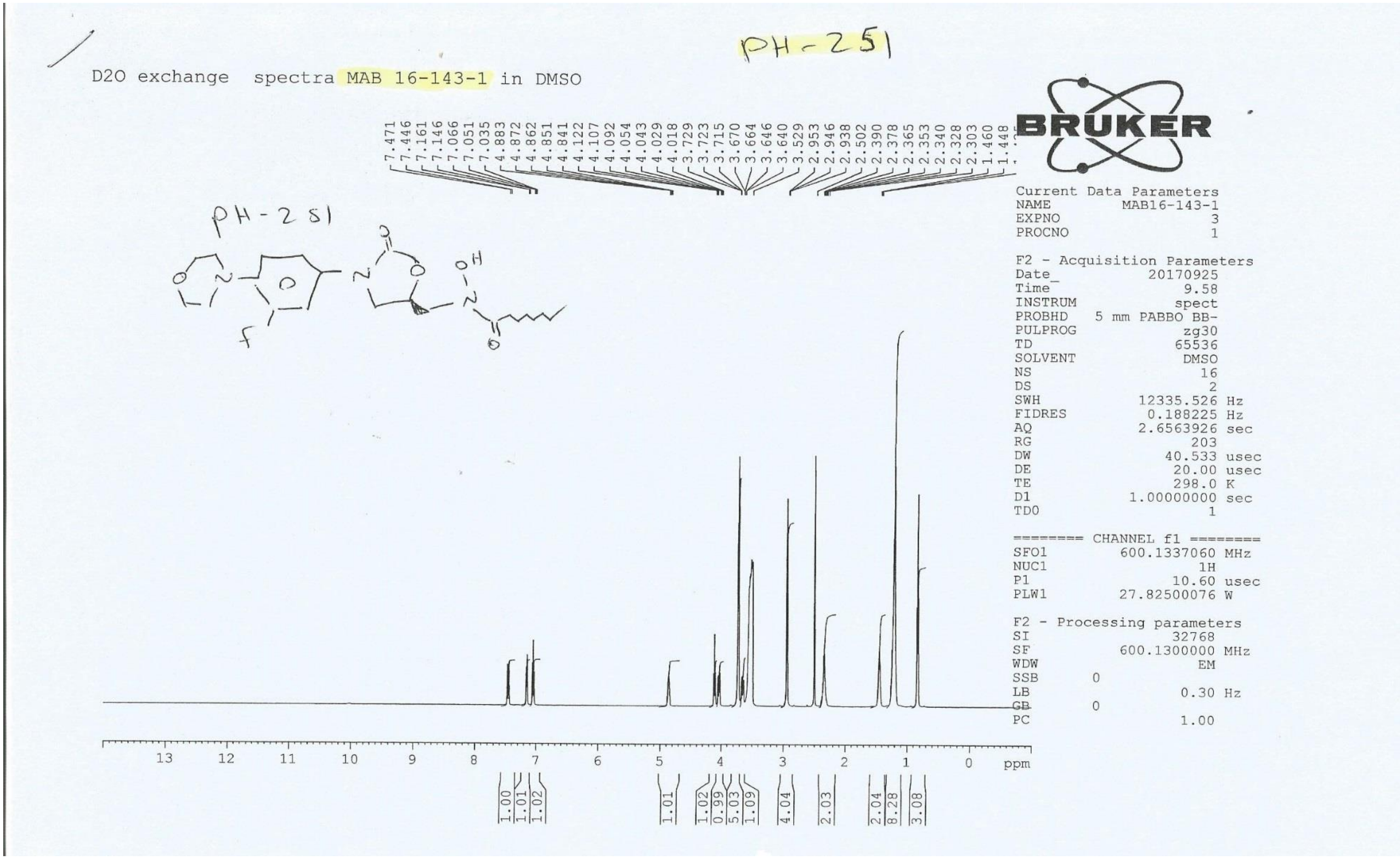
PH-251
D2O exchange spectra MAB 16-143-1 in DMSO
PH-251
7.471
7.446
7.161
7.146
7.066
7.051
7.035
4.883
4.872
4.862
4.851
4.841
4.122
4.107
4.092
4.054
4.043
4.029
4.018
3.729
3.723
3.715
3.670
3.664
3.646
3.640
3.529
2.953
2.946
2.938
2.502
2.390
2.378
2.365
2.353
2.340
2.328
2.303
1.460
1.448
BRUKER
Current Data Parameters
NAME MAB16-143-1
EXPNO 3
PROCNO 1
F2 - Acquisition Parameters
Date_ 20170925
Time 9.58
INSTRUM spect
PROBHD 5 mm PABBO BB-
PULPROG zg30
TD 65536
SOLVENT DMSO
NS 16
DS 2
SWH 12335.526 Hz
FIDRES 0.188225 Hz
AQ 2.6563926 sec
RG 203
DW 40.533 usec
DE 20.00 usec
TE 298.0 K
D1 1.00000000 sec
TD0 1
======== CHANNEL f1 ========
SFO1 600.1337060 MHz
NUC1 1H
P1 10.60 usec
PLW1 27.82500076 W
F2 - Processing parameters
SI 32768
SF 600.1300000 MHz
WDW EM
SSB 0
LB 0.30 Hz
GB 0
PC 1.00
13 12 11 10 9 8 7 6 5 4 3 2 1 0 ppm
1.00
1.01
1.02
1.01
1.02
0.99
5.03
1.09
4.04
2.03
2.04
8.28
3.08

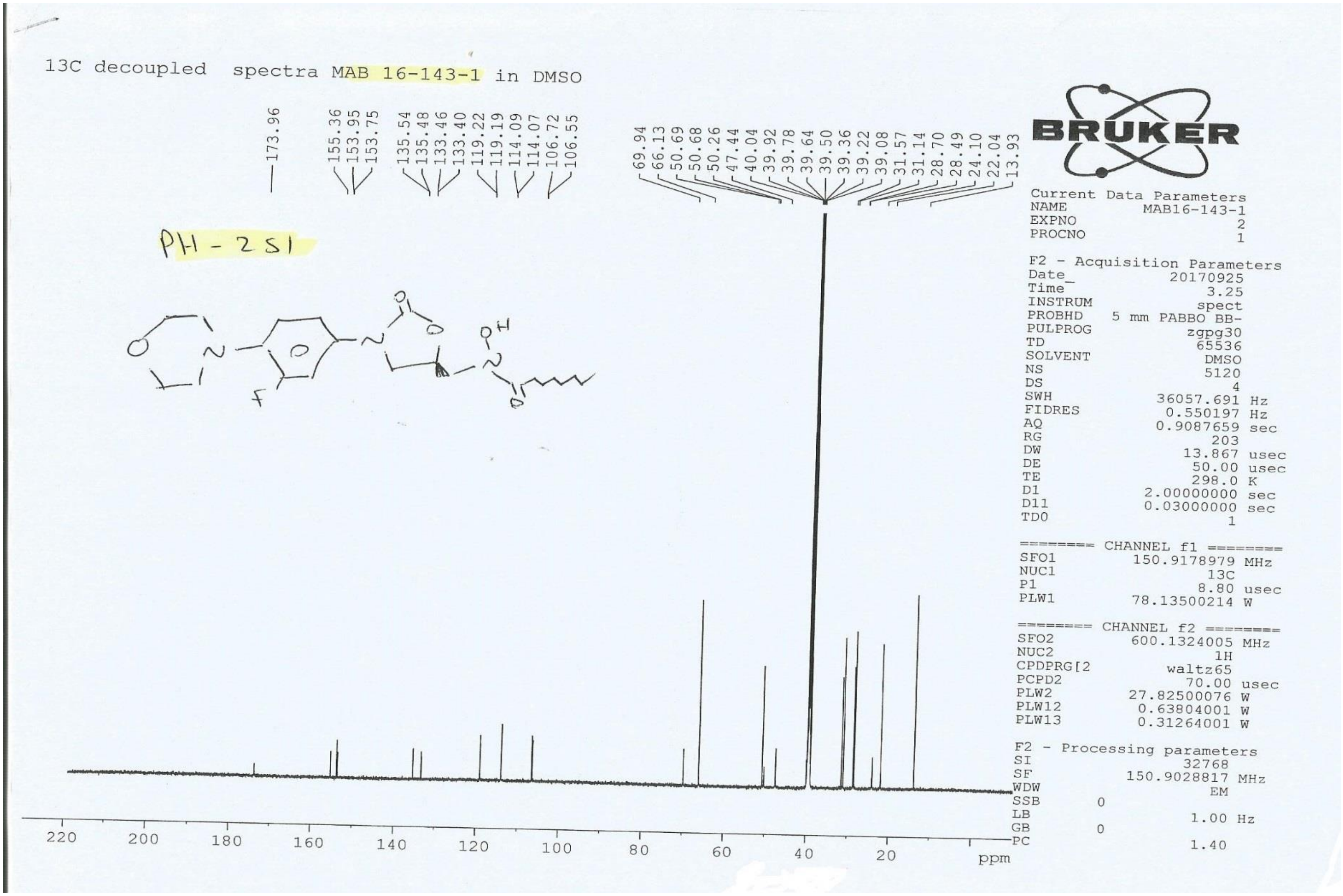
13C decoupled spectra MAB 16-143-1 in DMSO
173.96
155.36
153.95
153.75
135.54
135.48
133.46
133.40
119.22
119.19
114.09
114.07
106.72
106.55
69.94
66.13
50.69
50.68
50.26
47.44
40.04
39.92
39.78
39.64
39.50
39.36
39.22
39.08
31.57
31.14
28.70
28.49
24.10
22.04
13.93
PH - 251
BRUKER
Current Data Parameters
NAME MAB16-143-1
EXPNO 2
PROCNO 1
F2 - Acquisition Parameters
Date_ 20170925
Time 3.25
INSTRUM spect
PROBHD 5 mm PABBO BB-
PULPROG zgpg30
TD 65536
SOLVENT DMSO
NS 5120
DS 4
SWH 36057.691 Hz
FIDRES 0.550197 Hz
AQ 0.9087659 sec
RG 203
DW 13.867 usec
DE 50.00 usec
TE 298.0 K
D1 2.00000000 sec
D11 0.03000000 sec
TD0 1
======== CHANNEL f1 ========
SFO1 150.9178979 MHz
NUC1 13C
P1 8.80 usec
PLW1 78.13500214 W
======== CHANNEL f2 ========
SFO2 600.1324005 MHz
NUC2 1H
CPDPRG[2 waltz65
PCPD2 70.00 usec
PLW2 27.82500076 W
PLW12 0.63804001 W
PLW13 0.31264001 W
F2 - Processing parameters
SI 32768
SF 150.9028817 MHz
WDW EM
SSB 0
LB 1.00 Hz
GB 0
PC 1.40
220 200 180 160 140 120 100 80 60 40 20 ppm

Research Sector Projects Unit- GS 01/05
Kuwait University

RSPU
Research Sector Projects Unit
KUWAIT UNIVERSITY

FT- IR Spectral Data

PH - 251

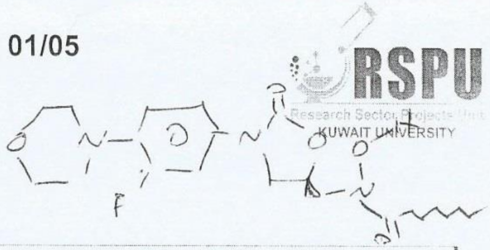

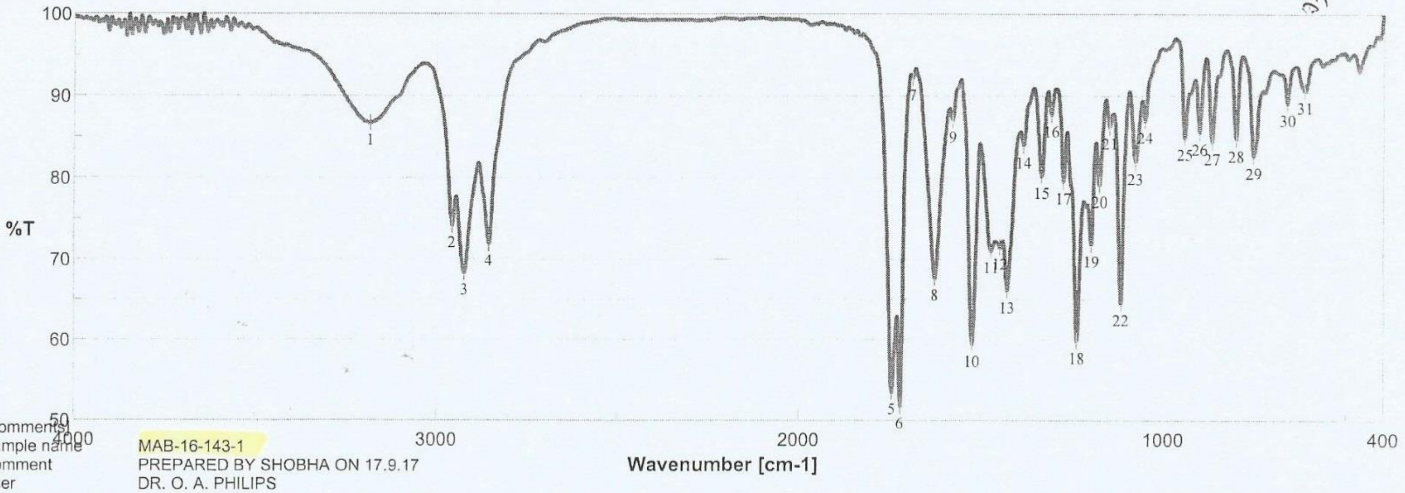

[Comments]
Sample name MAB-16-143-1
Comment PREPARED BY SHOBHA ON 17.9.17
User DR. O. A. PHILIPS
Division RSPU GS01/05
Company Kuwait University

[Measurement Information]
Model Name FT/IR-6300typeA
Serial Number A009861024

Light Source Standard
Detector TGS
Accumulation Auto (50)
Resolution 4 cm-1
Zero Filling On
Apodization Cosine
Gain Auto (4)
Aperture Auto (7.1 mm)
Scanning Speed Auto (2 mm/sec)
Filter Auto (10000 Hz)

Result of Peak Picking

No.	Position	Intensity	No.	Position	Intensity	No.	Position	Intensity	No.	Position	Intensity
1	3183.9	86.6468	2	2956.34	74.1712	3	2922.59	68.2431	4	2854.13	71.9215
5	1743.33	53.5034	6	1720.19	51.7359	7	1687.41	92.1574	8	1625.7	67.6091
9	1575.56	87.0263	10	1523.49	59.3854	11	1471.42	71.0669	12	1446.35	71.4623
13	1427.07	66.0333	14	1380.78	83.8413	15	1331.61	80.1582	16	1305.57	87.6741
17	1271.82	79.484	18	1235.18	59.7641	19	1196.61	71.7915	20	1173.47	79.0443
21	1146.47	86.1378	22	1114.65	64.4374	23	1075.12	81.8439	24	1049.09	86.8978
25	941.092	84.8033	26	900.594	85.4115	27	865.882	84.2929	28	801.278	84.5928
29	753.066	82.5346	30	661.464	89.0266	31	613.252	90.5222			
